# Volatile Additives Engineering: A Trustworthy Morphology Control Strategy in Non‐Fullerene Organic Solar Cells

**DOI:** 10.1002/advs.77805

**Published:** 2026-09-16

**Authors:** Jianqiang Qin, Zhiqin Lai, Jiang‐Ling Shi, Zheng Xiao, Yuwei Duan, Zhiping Hu, Juan Du, Qiang Peng

**Affiliations:** ^1^ College of Materials and Chemistry & Chemical Engineering Chengdu University of Technology Chengdu P. R. China; ^2^ School of Physics and Optoelectronic Engineering Hangzhou Institute for Advanced Study University of Chinese Academy of Sciences Hangzhou P. R. China; ^3^ School of Chemical Engineering and State Key Laboratory of Advanced Polymer Materials Sichuan University Chengdu P. R. China

**Keywords:** active layer, film‐forming kinetics, morphology control, organic solar cells, power conversion efficiency, volatile additives

## Abstract

Over the past few years, the efficiency of organic solar cells (OSCs) has significantly increased from about 15% to 21%. The morphology of the photoactive layer plays a critical role in performance improvement of OSCs. Therefore, numerous methods have been developed to optimize the morphology of the photoactive layer and unlock the full potential of the devices. Among them, volatile solid and solvent additives have attracted considerable attention due to their robust enhancement of efficiency and stability, coupled with a simple operational process. Here, the classification and development history of the volatile solid and solvent additives are comprehensively summarized, and the underlying mechanisms of morphology optimization are discussed. Additionally, a summary of volatile additive‐induced improvements in interfacial layer performance is provided. Finally, the challenges and prospects for volatile additives are presented.

## Graphical abstract

In this review, the classification and development history of the volatile solid and solvent additives are comprehensively summarized, the underlying mechanisms of morphology optimization are discussed, and a summary of volatile additive‐induced improvements in interfacial layer performance is provided. Finally, the challenges and prospects for volatile additives are presented.

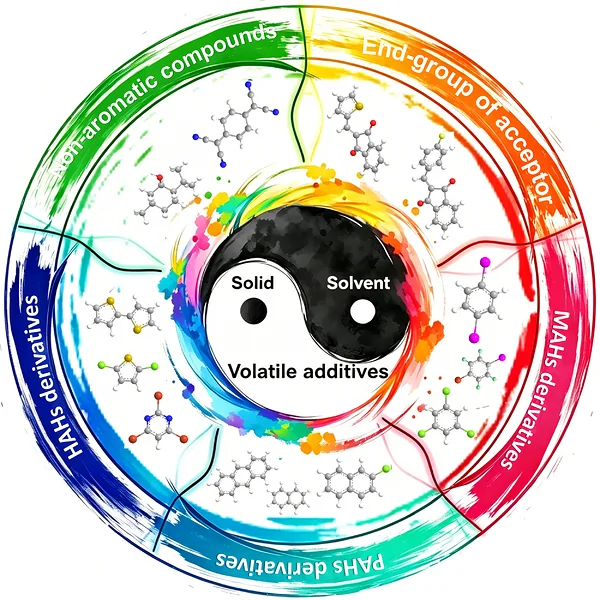

## 1 Introduction

Organic solar cell (OSC) is a highly promising type of photovoltaic devices that can directly convert sunlight into electricity. In recent years, with the continuous development of narrow‐bandgap non‐fullerene acceptors (NFAs), such as ITIC and Y6, the power conversion efficiency (PCE) of state‐of‐the‐art OSCs has exceeded 21% [1, 2, 3, 4, 5, 6, 7]. Typically, a high‐performance OSC consists of electrodes, interfacial modification layers and the bulk heterojunction (BHJ) active layer, in which the donor (D) and acceptor (A) materials are intermixed to form a bicontinuous interpenetrating network structure (Figure 1) [8, 9]. Apart from high‐performance donor and acceptor materials, the morphology of active layer also plays a critical role for improving photovoltaic performance of OSCs. Specifically, the morphology of the active layer can critically govern exciton dissociation, charge transport, extraction and recombination processes, thereby directly determining the photovoltaic performance [10]. Hence, it is very important to obtain an ideal morphology of the active layer with favorable molecular aggregation and packing, appropriate nanophase separation and domain size, as well as suitable vertical phase distribution.

**FIGURE 1:**
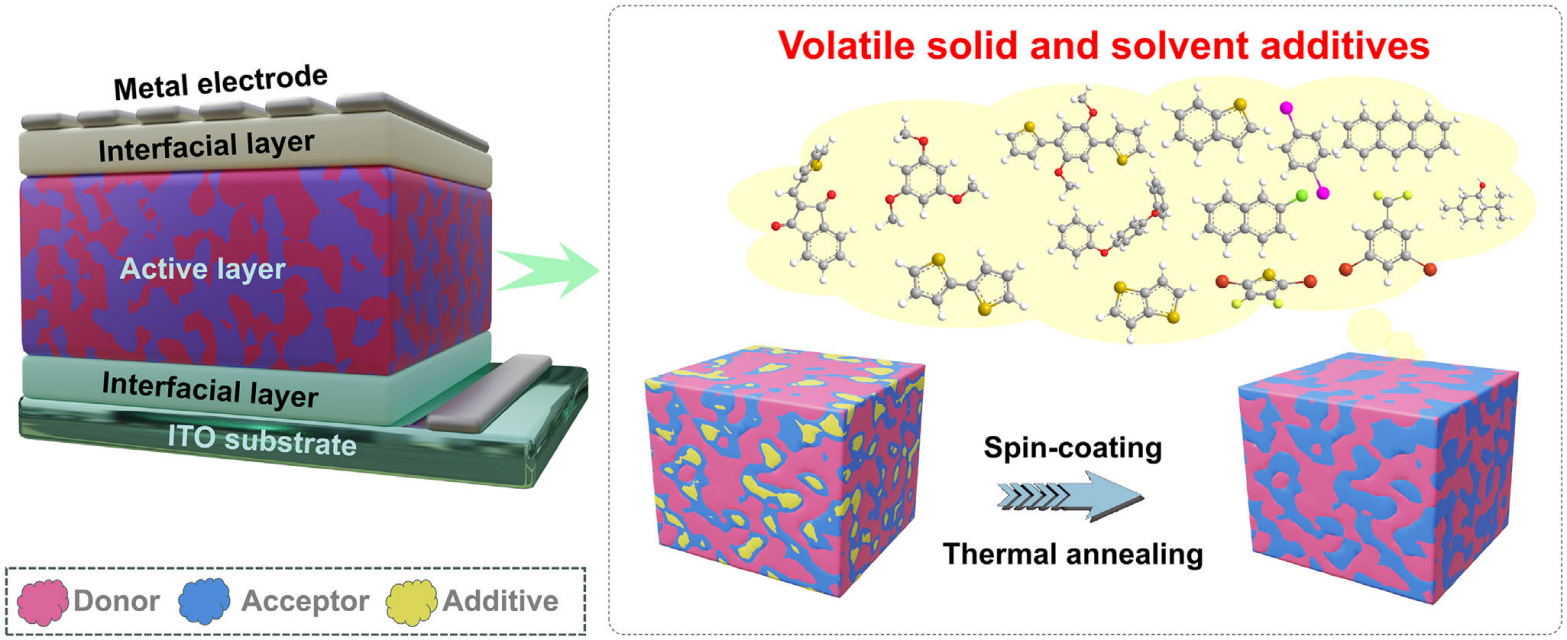
The schematic diagram of OSC device structure and the application of volatile additives in the active layer.

Numerous efforts have been made to optimize the morphology of active layer, including solvent vapor annealing [11], thermal annealing [12], additive engineering [13], and sequential deposition (SD or layer‐by‐layer (LbL)) method [14, 15], *etc*. The studies show that the additive strategy is a simple but effective approach for morphology control. According to the physical properties of additives, they can be classified into traditional high‐boiling‐point solvent additives, non‐volatile solid additives, as well as volatile solid and solvent additives. Initially, Heeger and co‐workers successively used alkanedithiols and 1,8‐di(R)octanes as solvent additives to regulate the morphology of active layer in the fullerene‐based OSCs. Meanwhile, they proposed two criteria for the solvent additives: (i) selective solubility of the fullerene acceptors; (ii) possessing a higher boiling point compared to the host solvent [16, 17]. Many high‐boiling‐point solvent additives thus have been developed in the fullerene‐based OSCs and subsequently employed successfully in the prevailing non‐fullerene systems [18]. Although highly effective, the high‐boiling‐point additives‐treated devices exhibit poor reproducibility owing to the stringent content control of additives (or the tedious optimization of additive ratios). Furthermore, the residues of additives would severely impact the morphology of active blend film, leading to the poor device stability [19, 20]. Subsequently, the advent of volatile additives has addressed well the aforementioned issues and emerged rapidly as a research hotspot in this field of OSCs.

Herein, we primarily focus on the emerging volatile solid and solvent additives in OSCs. First, the development of volatile solid and solvent additives as well as their impact on device performance are comprehensively reviewed. Second, the working mechanisms of volatile additives are discussed in detail. Lastly, the opportunities and challenges confronting volatile additives are also outlined. Our goal is to inspire researchers to develop novel and highly efficient volatile additives, thereby achieving efficiency breakthroughs and advancing the industrialization process of OSCs in the future.

## 2 Application of Volatile Additives in the Photoactive Layer

The additive strategy is a simple yet effective method that involves pre‐mixing additive into the active layer solution. However, unlike high‐boiling‐point solvent additives and non‐volatile solid additives, volatile additives can be effectively removed during the film‐casting process or the subsequent post‐annealing treatment at an appropriate temperature (Figure 1). The volatility of additives is determined by comparing the presence or absence of their characteristic signals, derived from optical images, thermogravimetric analysis (TGA) and Fourier transform infrared spectroscopy (FT‐IR) [21, 22, 23, 24], or X‐ray photoelectron spectroscopy (XPS) [25, 26, 27], UV‐visible spectroscopy [28, 29, 30], thin‐layer chromatography (TLC) [31], high‐resolution mass spectrometry (HRMS) and nuclear magnetic resonance (NMR) [32], before and after volatilization. It is noteworthy that the combined use of multiple analytical methods can effectively analyze the volatility of additives.

### 2.1 The Categorization and Development of Volatile Additives

In recent years, a large number of volatile additives have been developed and successfully applied to various OSC systems (Figures 2 and 3) due to its significant advantages in efficiency enhancement, stability and reproducibility. According to their structures, volatile additives can be classified into five types: (i) End‐groups of the non‐fullerene acceptor; (ii) Monocyclic aromatic hydrocarbons (MAHs) derivatives; (iii) Polycyclic aromatic hydrocarbons (PAHs) derivatives; (iv) Heterocyclic aromatic hydrocarbons (HAHs) derivatives; and (v) non‐aromatic compounds. In the following sections, we will discuss each of them in detail.

**FIGURE 2:**
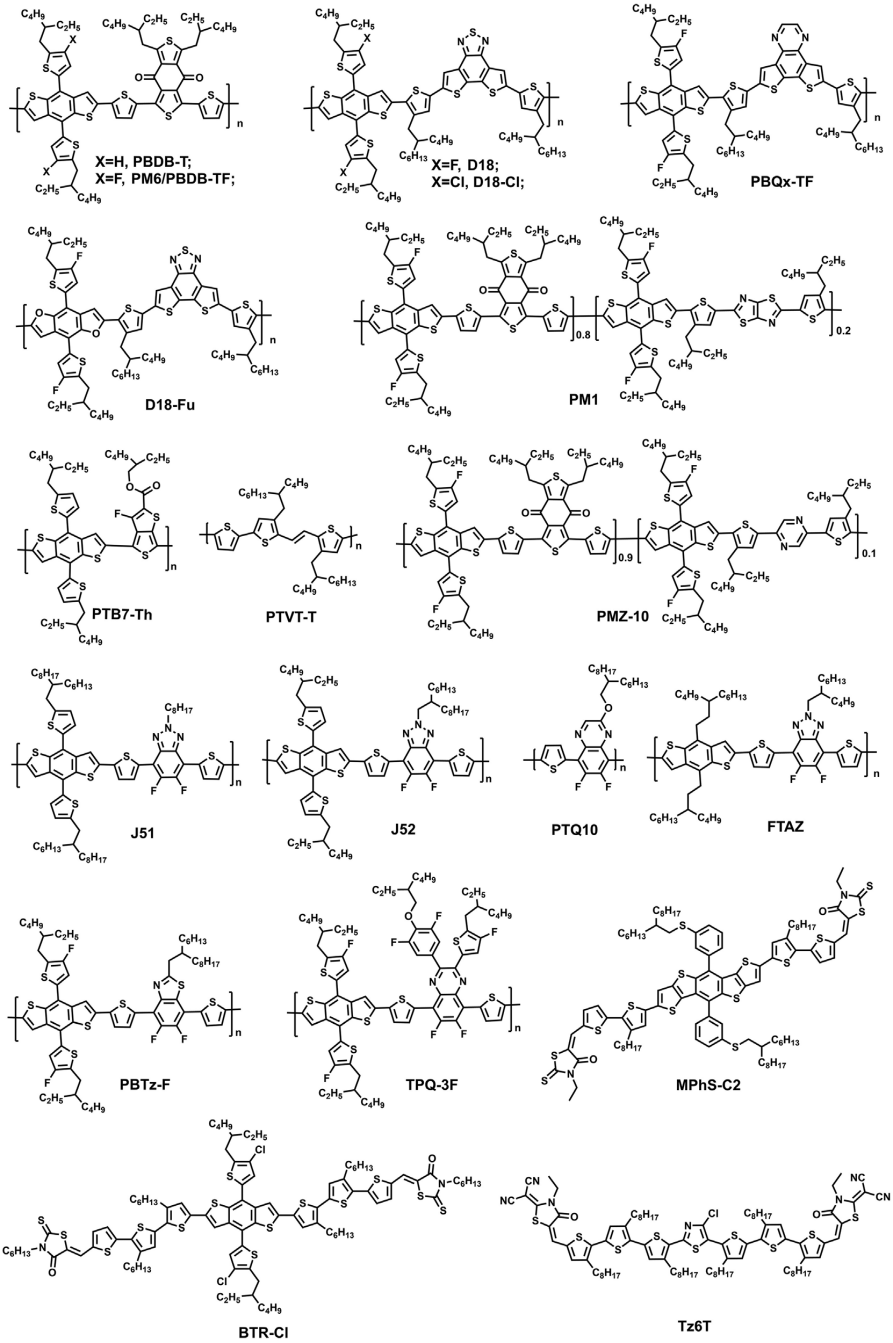
Chemical structures of donors mentioned in section 2.1.

**FIGURE 3:**
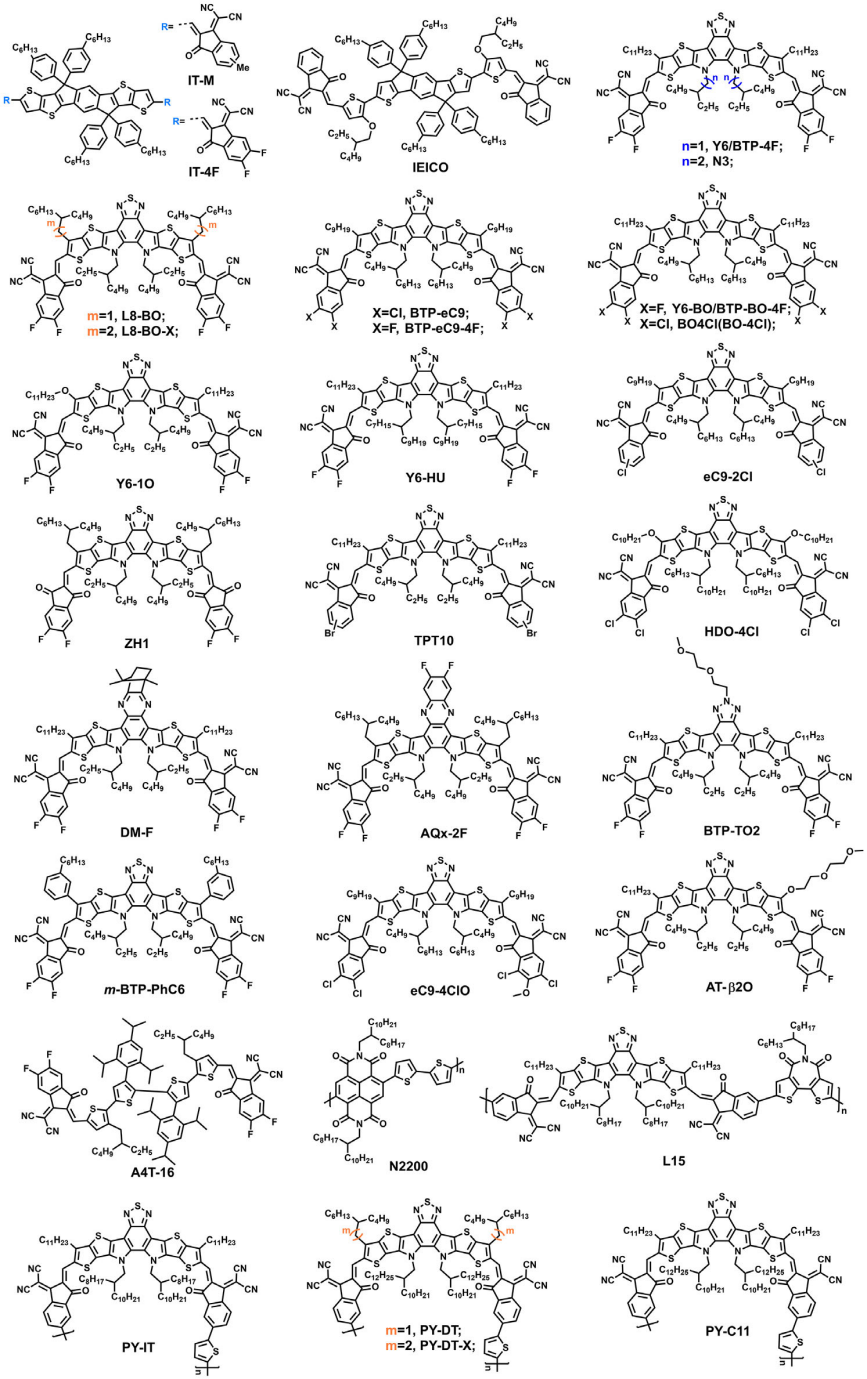
Chemical structures of acceptors mentioned in section 2.1.

#### 2.1.1 End‐Groups of the Non‐Fullerene Acceptor

It is worth noting that Hou's group has pioneered the use of volatile solid additives based on the end‐groups of the non‐fullerene acceptors. In 2018, Yu et al. designed and synthesized a series of solid additives (SA‐1 to SA‐8) with structures similar to those of the non‐fullerene acceptor IT‐4F (Figure 4) [33]. As described in Figure 5a, after removing additive SA‐1, the acceptor IT‐4F exhibits more ordered and condensed molecular stacking, leading to enhanced charge transport. Ultimately, the efficiency of the PBDB‐TF:IT‐4F‐based device has been significantly improved from 12.2% to 13.8% after the volatile solid additive SA‐1 treatment. Moreover, the SA‐1‐treated device maintains good performance when the SA‐1 content varies over a wide range (8‐25 wt.%) and exhibits superior stability compared to the 1,8‐diiodooctane (DIO)‐treated device. This work demonstrates that volatile solid additives represent a promising morphology control strategy, and has initiated a research upsurge in the field of OSCs. Subsequently, Yu et al. further studied the effects of the volatile additives (SA‐4 and SA‐7) with different volatility characteristics on the morphology of the active layer and the performance of OSCs [20]. Studies indicate that both SA‐4 and SA‐7 with similar structures can regulate the molecular arrangement of the non‐fullerene acceptor IT‐4F. However, after the thermal annealing treatment, SA‐4 can volatilize completely, while SA‐7 remains in the active layer due to its low volatility. As a result, the PBDB‐TCl:IT‐4F‐based device exhibits an enhanced PCE from 12.1% to 13.5% after SA‐4 treatment, whereas the SA‐7‐processed device show deteriorated photovoltaic performance. This finding shows that the volatility of additives is a key factor in the design or selection of additives. Moreover, they also employed additive SA‐4 in P3HT:ZY‐4Cl‐based OSCs [34]. As expected, the SA4‐processed devices afford a higher PCE of 10.24% compared to the DIO‐treated counterpart (6.26%).

**FIGURE 4:**
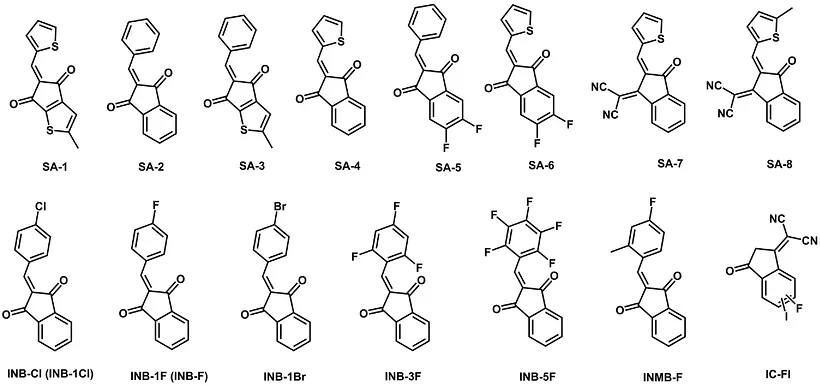
Chemical structures of volatile additives based on end‐groups of the non‐fullerene acceptors.

**FIGURE 5:**
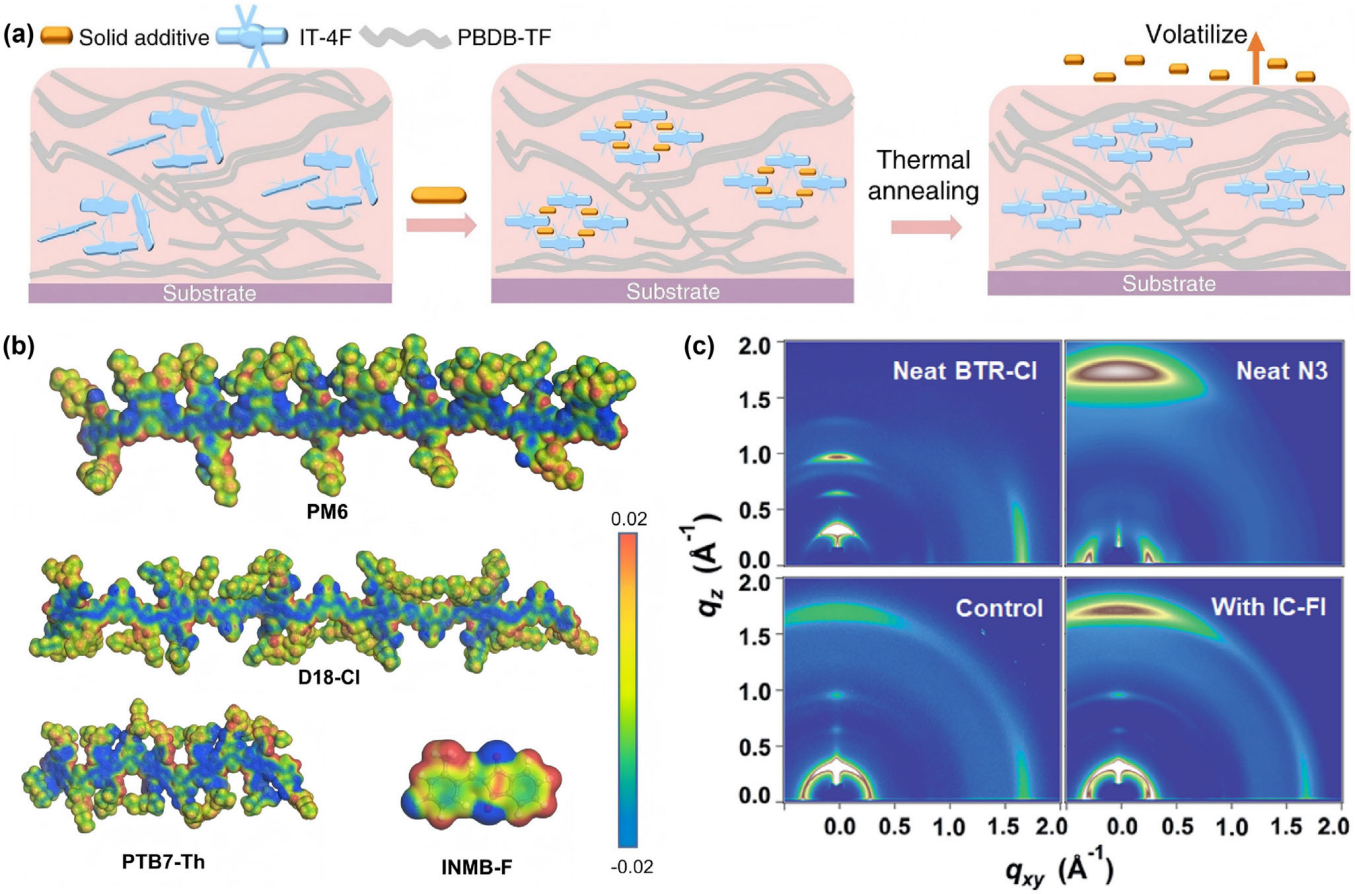
(a) Schematic diagram of the working mechanism of solid additives. Reproduced with permission [33]. Copyright 2018, Springer Nature. (b) Electrostatic potential (ESP) distribution of INMB‐F, PM6, D18‐Cl and PTB7‐Th. Reproduced with permission [35]. Copyright 2023, Springer Nature. (c) 2D grazing‐incidence wide‐angle X‐ray scattering (GIWAXS) patterns of the neat films BTR‐Cl and N3, and blend films BTR‐Cl:N3 and BTR‐Cl:N3(IC‐FI). Reproduced with permission [36]. Copyright 2021, Royal Society of Chemistry.

In addition, Wang's group has done significant work on this category of additives [28, 35, 37, 38, 39]. In 2020, Cai et al. developed three novel solid additives (i.e., INB‐1F, INB‐3F, and INB‐5F) with different degrees of fluorination to control the morphology in non‐fullerene OSCs [28]. The results demonstrate that these additives display higher volatility when the number of fluorine atoms increases and can evaporate during moderate thermal annealing. After incorporating these additives, the PBDB‐T‐2F:BTP‐4F and PBDB‐T‐2F:IT‐4F‐based devices show improved PCE from 15.2% to 16.5% and from 12.2% to 13.4%, respectively. Following this, Zhang et al. reported two volatile solid additives INB‐F (INB‐1F) and INB‐Cl to replace the commonly used solvent additive DIO in PM6:Y6‐BO (or Y7‐BO) OSCs [37]. Notably, both additives of INB‐F and INB‐Cl lead to remarkable enhancements in both efficiency and operational stability of the related devices. Specifically, the INB‐F and INB‐Cl‐treated PM6:Y6‐BO binary OSCs afford high PCEs of 16.7% and 16.3%, respectively, which are higher than that of the untreated devices (15.1%). Moreover, the INB‐F and INB‐Cl‐treated devices display superior stability (*T*_80_ = 523 and 120 h, respectively) compared to both the control devices (*T*_80_ = 66.2 h) and the DIO‐processed devices (*T*_80_ = 6.6 h). In 2023, Gan et al. applied the conjugated molecule INMB‐F to tune the intermolecular stacking of benzo[1,2‐*b*:4,5‐*b*']dithiophene (BDT)‐based polymer donors, such as PM6, D18‐Cl, and PTB7‐Th, via electrostatic force (dipole‐dipole interaction, Figure 5b), leading to improved π‐π stacking and reduced stacking distance [35]. As listed in Table 1, the PM6/L8‐BO binary devices fabricated via LbL method show enhanced short‐circuit current density (*J*_sc_) and fill factor (FF), yielding a high PCE of 19.4% (certified 18.96%). In 2024, Gan et al. further introduced the solid additive INMB‐F and solvent additive 1‐chloronaphthalene (1‐CN) into the polymer acceptor PY‐IT to fine‐tune the morphology in all‐polymer solar cells (all‐PSCs) [38]. Finally, the LbL deposited PM6/PY‐IT devices with dual‐additive treatment deliver a high PCE of 19.1%. Thereafter, Chen et al. employed a series of solid additives (i.e., INB‐5F, INB‐3F, INB‐1F, INB‐1Cl (INB‐Cl) and INB‐1Br) with varied electrostatic potential distribution to study the interaction mechanism between solid additives and donor [39]. While the dione unit of additives tends to adsorb onto the PM6 backbone, the reduced electrostatic potential of the halogen‐substituted benzene shifts the interaction site of benzene from the alkyl side chain toward the conjugated backbone of donor PM6, leading to ordered molecular stacking. As a result, a high PCE of 19.4% has been obtained in the LbL deposited PM6/L8‐BO OSCs.

**TABLE 1:** Summary of photovoltaic parameters for devices processed with volatile additives based on end‐groups of the non‐fullerene acceptor.

Active layer	Additive	*V*_oc_ [V]	*J*_sc_ [mA cm^−2^]	FF [%]	PCE [%]	Refs.
PBDB‐TF:IT‐4F	w/o	0.89	19.0	72	12.2	[33]
	SA‐1	0.87	20.6	77	13.8	
PBDB‐TCl:IT‐4F	w/o	0.907	19.0	70.3	12.1	[20]
	SA‐4	0.887	20.4	74.8	13.5	
P3HT:ZY‐4Cl	w/o	0.87	16.36	64	9.12	[34]
	DIO	0.80	12.41	63	6.26	
	SA4	0.90	17.00	67	10.24	
PBDB‐T‐2F:BTP‐4F	w/o	0.82	26.2	70.1	15.2	[28]
	INB‐5F	0.81	27.7	74.3	16.5	
PBDB‐T‐2F:IT‐4F	w/o	0.84	19.7	72.8	12.2	
	INB‐5F	0.83	21.5	73.8	13.4	
PM6:Y6‐BO	w/o	0.84	26.2	68.8	15.1	[37]
	INB‐F	0.82	26.5	76.7	16.7	
	INB‐Cl	0.82	27.0	74.2	16.3	
PM6/L8‐BO	w/o	0.882	26.56	78.9	18.4	[35]
	INMB‐F	0.883	26.94	81.3	19.4	
	INMB‐F	0.877	27.20	79.50	18.96^a^	
PM6/PY‐IT	w/o	0.958	25.5	63.1	15.2	[38]
	1‐CN&INMB‐F	0.947	26.1	77.7	19.1	
PM6/L8‐BO	w/o	0.881	26.9	78.3	18.5	[39]
	1NB‐1Br	0.883	27.3	80.7	19.4	
BTR‐Cl:N3	w/o	0.85	24.16	64.7	13.36	[36]
	IC‐FI	0.82	24.06	73.5	14.43	

^a^ Certified result.

In contrast to previous reports, in 2020, Chen et al. designed and synthesized a novel volatile solid additive IC‐FI to modulate the morphology of the active layer in BTR‐Cl:N3‐based all‐small‐molecule OSCs [36]. After additive IC‐FI treatment, an optimized microstructure with a dominant face‐on oriented molecular stacking is obtained in the blend films (Figure 5c), resulting in effectively improved charge transport and extraction process. Ultimately, the efficiency of the BTR‐Cl:N3‐based device has been significantly increased from 13.36% to 14.43% after incorporating additive IC‐FI.

As discussed above, the introduction of volatile solid additives with similar structure to the end‐group of the A‐D‐A or A‐DA'D‐A non‐fullerene acceptors can significantly optimize the molecular stacking (non‐fullerene acceptors or BDT‐based polymer donors), and thus facilitate charge carrier transport in the active layer, thereby improving the device performance (efficiency, stability and reproducibility). These findings offer valuable insights for the design and development of volatile solid additives.

#### 2.1.2 Monocyclic Aromatic Hydrocarbons (MAHs) Derivatives

Recently, some MAHs derivatives (e.g., halogenated benzenes and their derivatives) have been utilized as volatile solid and solvent additives to optimize the morphology of the active layer, which have attracted tremendous attention due to their high volatility and remarkable performance enhancement. Their molecular structures are shown in Figure 6, and the corresponding photovoltaic performance parameters are listed in Table 2. Additionally, its development history is discussed in detail as follows.

**FIGURE 6:**
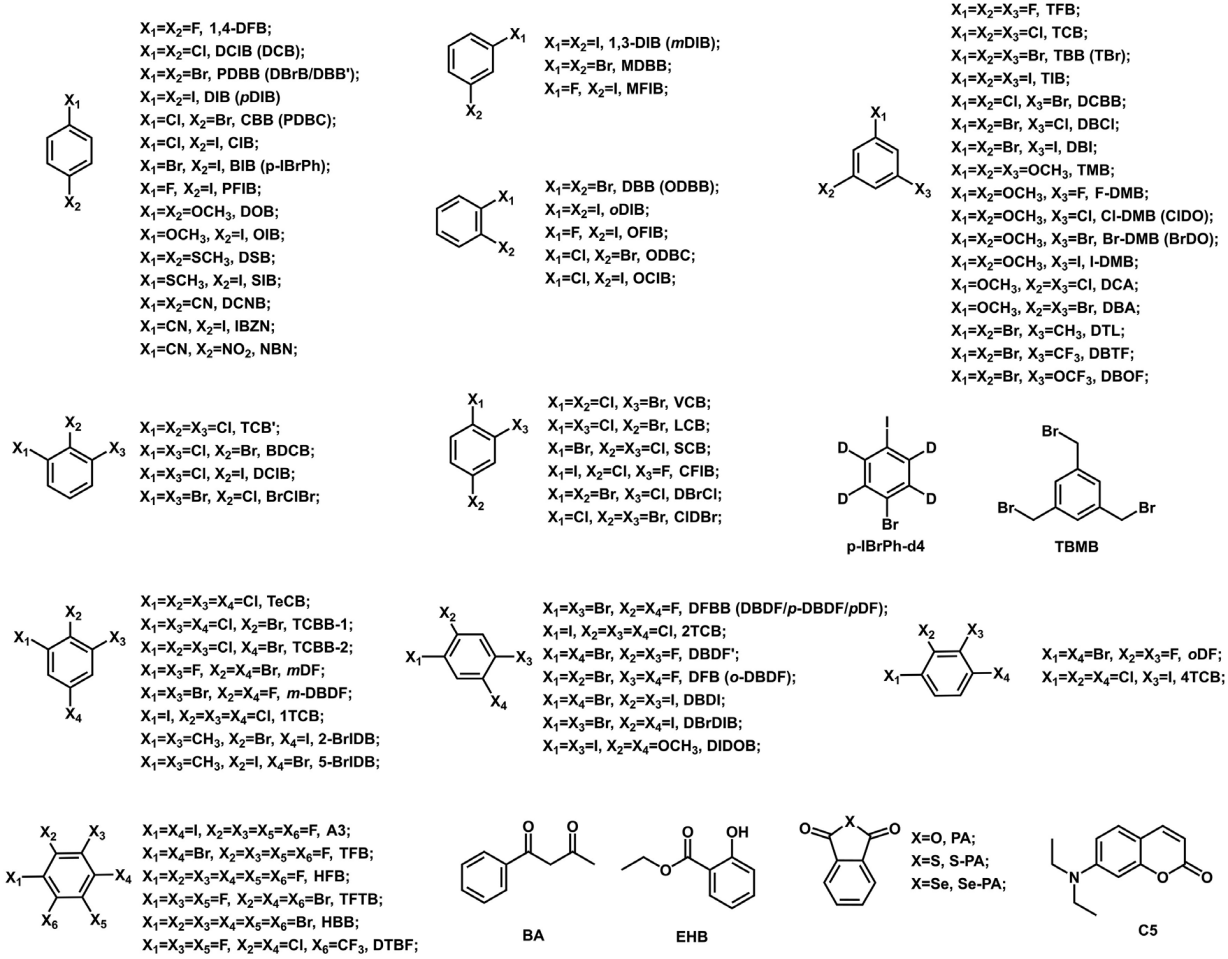
Chemical structures of volatile additives based on MAHs derivatives.

**TABLE 2:** Summary of photovoltaic parameters for devices processed with volatile additives based on MAHs derivatives.

Active layer	Additive	*V*_oc_ [V]	*J*_sc_ [mA cm^−2^]	FF [%]	PCE [%]	Refs.
PM6:Y6	w/o	0.86	25.29	68.40	14.8	[40]
	1‐CN	0.84	25.46	72.90	15.6	
	A3	0.82	26.50	76.05	16.5	
PM6:Y6	w/o	0.87	24.87	71.13	15.39	[41]
	DIB	0.84	26.33	78.22	17.36	
	1‐CN	0.85	26.07	71.71	15.97	
	DIO	0.82	26.20	75.72	16.27	
D18‐Cl/N3	w/o(BHJ)	0.871	26.74	75.1	17.49	[42]
	w/o(SD)	0.873	26.74	76.0	17.74	
	DIB(SD)	0.860	27.18	78.8	18.42	
PM6:Y6	w/o	0.87	25.08	71.22	15.36	[43]
	1,3‐DIB	0.85	26.00	76.46	16.90	
PM6:Y6:PC_61_BM	w/o	0.834	23.98	71.4	14.28	[44]
	1,4‐DFB	0.823	25.12	74.2	15.34	
D18‐Cl:N3	w/o	0.857	26.92	76.8	17.70	[45]
	TCB'	0.855	26.63	78.3	17.83	
	DBB	0.856	26.99	80.2	18.54	
PM6:L8‐BO	w/o	0.90	24.70	73.39	16.28	[27]
	ODBB	0.88	25.57	79.20	17.82	
	MDBB	0.89	25.72	79.38	18.17	
	PDBB	0.89	25.81	80.20	18.42	
PM6:Y6‐BO	w/o	0.82	24.4	74.7	15.1	[46]
	DIB	0.84	25.2	76.1	16.1	
	DBrB	0.84	26.2	76.8	17.0	
	DClB	0.84	25.0	75.5	15.9	
PM6:BTP‐eC9	w/o	0.853	25.7	74.7	16.5	[24]
	DIB	0.835	27.4	77.7	17.8	
	CIB	0.842	27.7	78.7	18.4	
	DCB	0.842	27.6	78.5	18.2	
PM6:D18‐Cl:L8‐BO	w/o	0.915	24.5	74.8	16.8	
	CIB	0.881	26.7	81.1	19.1	
PM6:BTP‐eC9	w/o	0.861	25.86	73.87	16.44	[47]
	DBB'	0.862	26.22	75.83	17.14	
	DIB	0.860	27.58	76.77	18.25	
	BIB	0.867	27.89	78.21	18.91	
D18:L8‐BO	w/o	0.900	25.64	78.49	18.11	[48]
	DCB	0.897	25.71	78.51	18.13	
	CBB	0.887	26.32	79.59	18.58	
	CIB	0.885	26.05	79.25	18.29	
PM6:L8‐BO (AM 1.5G)	w/o	0.91	24.87	71.45	16.17	[49]
	OCIB	0.89	26.15	80.01	18.30	
PM6:L8‐BO under indoor light	w/o	0.83	133.12	74.57	26.46	
	OCIB	0.81	138.54	80.97	29.52	
PM6:Y6	w/o	0.862	25.25	73.62	16.03	[50]
	p‐IBrPh‐d_4_	0.845	26.42	78.17	17.46	
	p‐IBrPh	0.847	25.74	77.52	16.89	
PM6:BTP‐eC9	w/o	0.857	27.77	76.11	18.12	
	p‐IBrPh‐d_4_	0.856	28.27	79.81	19.32	
PM6:Y6	w/o	0.860	25.23	70.78	15.36	[29]
	DCIB	0.849	26.49	75.69	17.02	
PM6:L8‐BO	w/o	0.90	23.1	72.0	15.0	[25]
	DBCl	0.89	25.9	80.2	18.5	
PM6:BTP‐eC9	DIO	0.836	27.48	78.26	17.98	[51]
	TCB	0.861	27.88	80.39	19.31	
	TCB	0.859	27.86	79.16	18.93^a^	
PM6:Y6	w/o	0.853	25.8	73.6	16.2	[52]
	TCB	0.820	26.9	78.6	16.9	
	TBB	0.837	27.3	78.3	17.9	
	TIB	0.836	26.5	77.6	17.2	
PBQx‐TF:eC9‐2Cl	w/o	0.890	25.9	73.1	16.9	[53]
	DIO	0.869	26.6	78.4	18.1	
	DCBB	0.879	27.2	80.4	19.2	
	DCBB	0.877	27.1	79.8	19.0^a^	
PM6:L8‐BO	BDCB (BHJ)	0.888	25.92	78.9	18.12	[54]
	BDCB (LbL)	0.891	26.80	79.7	19.03	
D18‐Cl:N3	w/o	0.862	27.24	75.8	17.80	[55]
	CFIB	0.848	27.53	79.4	18.54	
D18‐L8‐BO	w/o	0.899	26.08	73.75	17.29	[56]
	LCB	0.909	26.20	79.60	18.96	
	SCB	0.867	25.57	81.05	17.97	
	VCB	0.910	26.43	80.35	19.33	
PM6/L8‐BO	w/o	0.915	26.7	71.3	17.4	[57]
	BrClBr	0.897	27.8	79.5	19.9	
	DBrCl	0.885	27.8	78.3	19.3	
	ClDBr	0.892	27.6	77.0	19.0	
PM6:Y6	w/o	0.858	26.13	72.12	16.17	[58]
	TBB	0.831	27.18	76.12	17.20	
	TBMB	0.823	27.68	78.63	17.92	
D18:L8‐BO	w/o^b^	0.917	26.44	76.88	18.64	
	TBMB^b^	0.905	28.29	78.23	20.04	
PM6:Y6	w/o	0.852	24.13	73.49	15.12	[59]
	1‐CN	0.843	25.88	73.92	16.13	
	DFB	0.841	26.68	76.41	17.15	
	TFB	0.841	26.43	73.15	16.30	
PM6:BTP‐eC9	w/o	0.853	24.21	74.48	15.45	
	DFB	0.851	27.24	78.37	18.17	
PM6:L8‐BO	w/o	0.891	24.65	75.63	16.61	
	DFB	0.881	26.25	79.84	18.46	
PM6:Y6‐BO	w/o	0.83	25.2	74.4	15.6	[60]
	DBrDIB	0.84	26.8	78.5	17.9	
PM6:Y6‐HU	w/o	0.86	26.0	77.0	17.2	
	DBrDIB	0.87	27.2	80.4	19.1	
PM6:L8‐BO	w/o	0.900	25.6	73.8	17.0	[61]
	DBB'	0.894	25.9	78.5	18.2	
	DFBB	0.898	26.4	80.8	19.2	
D18:N3: Y6‐1O	w/o	0.878	26.8	78.1	18.4	[62]
	DBDF	0.876	28.5	80.9	20.2	
TPQ‐3F:Y6	w/o	0.838	26.09	69	15.16	[63]
	*p*‐DBDF	0.842	25.85	70	15.29	
	*o*‐DBDF	0.837	25.99	71	15.50	
	*m*‐DBDF	0.835	26.68	70	15.71	
PM6:L8‐BO	w/o	0.915	25.63	73.9	17.37	[64]
	DBDF'	0.910	25.72	78.2	18.34	
	DBDI	0.905	26.80	80.3	19.42	
D18:L8‐BO	w/o	0.881	26.29	79.29	18.36	[32]
	TeCB	0.870	27.29	80.67	19.16	
PM6:L8‐BO	w/o	0.901	25.02	71.37	16.09	[47]
	TCBB‐1	0.892	27.03	78.50	18.93	
	TCBB‐2	0.882	26.20	77.95	18.01	
PM6:L8‐BO	w/o	0.889	25.63	77.81	17.72	[65]
	*o*DF	0.867	25.69	77.59	17.28	
	*m*DF	0.885	27.01	80.66	19.28	
	*p*DF	0.883	26.91	78.04	18.54	
D18:L8‐BO	*m*DF	0.908	27.09	81.63	20.08	
D18:L8‐BO	w/o	0.910	26.52	81.60	19.70	[66]
	4TCB	0.904	26.98	81.63	19.91	
	2TCB	0.903	27.21	82.97	20.38	
	1TCB	0.901	27.69	83.93	20.94	
PBDB‐TF:eC9	DIO	0.837	26.44	79.38	17.57	[31]
	TMB	0.854	27.26	79.95	18.61	
PBDB‐TF:HDO‐4Cl:eC9	DIO	0.863	26.59	79.57	18.27	
	TMB	0.883	27.27	80.17	19.30	
PM6:L8‐BO	w/o	0.901	24.39	74.84	16.44	[67]
	F‐DMB	0.906	25.07	76.96	17.48	
	Cl‐DMB	0.909	26.18	78.12	18.59	
	Br‐DMB	0.915	26.82	79.58	19.53	
	I‐DMB	0.919	27.41	80.98	20.40	
PM6:L8‐BO	w/o	0.905	25.27	74.56	17.05	[68]
	DIO	0.874	26.11	78.48	17.92	
	BrDO	0.893	26.20	78.62	18.40	
	ClDO	0.888	26.34	79.12	18.51	
D18:L8‐BO:ZH1	w/o	0.936	26.26	77.70	19.10	[69]
	Cl‐DMB	0.932	27.30	79.25	20.16	
	Cl‐DMB	0.931	27.31	77.72	19.76^a^	
PM6:L8‐BO	w/o	0.907	24.77	73.36	16.49	[70]
	DOB	0.900	25.05	75.60	17.05	
	OIB	0.910	25.86	75.78	17.83	
	DIB	0.893	26.17	79.43	18.57	
	SIB	0.898	26.30	79.92	18.87	
	DSB	0.858	24.60	73.90	15.59	
PBTz‐F:L8‐BO	SIB	0.905	26.57	78.23	18.81	
PM6:PBTz‐F:L8‐BO	SIB	0.907	27.10	80.06	19.68	
PM6:L8‐BO	w/o	0.897	25.89	74.17	17.23	[71]
	OIB	0.886	26.75	78.40	18.58	
PM6:L8‐BO‐X	w/o	0.881	26.71	76.4	17.98	[72]
	DIDOB	0.878	27.46	81.9	19.75	
D18:PM6:L8‐BO	w/o	0.919	26.54	78.9	19.24	
	DIDOB	0.904	27.20	81.8	20.11	
	DIDOB	0.911	27.16	80.92	20.03^a^	
D18:BTP‐eC9‐4F	w/o	0.876	27.67	74.53	18.08	[73]
	CNB	0.874	28.22	77.01	19.00	
	DCNB	0.880	28.45	77.60	19.43	
D18:BTP‐eC9	w/o	0.856	28.68	78.00	19.16	[74]
	NBN	0.862	28.88	81.24	20.22	
D18:L8‐BO:BTP‐eC9	w/o	0.881	27.90	79.44	19.52	
	NBN	0.880	28.62	81.31	20.49	
PM6:L8‐BO	w/o	0.905	25.46	75.87	17.49	[75]
	IBZN	0.898	26.28	79.54	18.77	
PM6:L8‐BO	w/o	0.905	25.55	71.92	16.63	[76]
	DIO	0.890	26.69	76.71	18.23	
	DTL	0.906	27.06	76.92	18.87	
D18:Y6	w/o	0.869	26.87	75.14	17.54	[77]
	TBB	0.860	27.30	76.63	18.00	
	DBTF	0.871	27.35	76.75	18.28	
	DBOF	0.875	27.40	78.30	18.78	
D18‐Cl/BTP‐4F	w/o	0.878	26.28	76.04	17.55	[78]
	DTBF	0.887	27.21	78.60	19.00	
D18/L8‐BO	w/o	0.920	26.03	77.36	18.53	[79]
	2‐BrIDB	0.925	27.48	81.85	20.81	
	5‐BrIDB	0.922	26.52	79.47	19.43	
PTB7‐Th:PC_71_BM	w/o	0.78	13.83	48.84	5.27	[80]
	DIO	0.78	16.46	68.12	8.75	
	EHB	0.79	17.40	70.12	9.64	
PM6:Y6	w/o	0.87	25.2	69.3	15.2	[81]
	1‐CN	0.85	25.7	74.9	16.4	
	BA	0.84	26.3	78.9	17.4	
PM6:L8‐BO	w/o	0.911	25.15	75.12	17.20	[82]
	PA	0.905	26.51	75.84	18.20	
	S‐PA	0.887	26.36	79.19	18.51	
	Se‐PA	0.900	25.62	77.84	17.95	
D18:L8‐BO	w/o	0.929	25.84	78.55	18.85	[83]
	C5	0.930	26.39	79.63	19.55	
	C6	0.932	26.79	81.40	20.32	

^a^ Certified result;

^b^ Under the same conditions, self‐assembled monolayer (SAM) is used as the anode interfacial layer.

In 2020, Fu et al. employed a σ‐hole‐containing solid additive, 1, 4‐diiodotetrafluorobenzene (A3) to optimize the morphology of the active layer in non‐fullerene OSCs (Figure 7a) [40]. With the introduction of additive A3, the PM6:Y6 blend film exhibits preferential face‐on orientation with more ordered and condensed molecular stacking, which can facilitate charge transport and suppress charge recombination. Ultimately, the efficiency of the PM6:Y6 OSCs has been dramatically increased from 14.8% to 16.5%, which is higher than that of the 1‐CN‐treated devices (15.6%). Next, Fu et al. reported a highly efficient and simple volatile solid additive of 1,4‐diiodobenzene (DIB) for usage in non‐fullerene OSCs [41]. After the additive DIB treatment, an optimized morphology with highly dense and ordered molecular arrangement can be obtained due to the eutectic phase formed between the additive DIB and the non‐fullerene acceptor. Hence, compared with the 1‐CN (15.97%) and DIO (16.27%) counterparts, the DIB‐treated PM6:Y6 OSCs afford a higher PCE of 17.36%, which also exhibit superior stability. Additionally, DIB not only operates effectively in a wide concentration range but also shows remarkable versatility in various systems. Interestingly, Xie et al. found that the additive DIB is an alcohol‐washable solid additive, which can be removed through alcohol washing, thus eliminating the necessity for post‐thermal annealing treatment [84]. This finding indicates that the additive DIB has potential for large‐scale production of OSCs. In 2022, we developed a joint strategy combining solid additive DIB with sequential deposition method to fine‐tune the morphology of the active layer [42]. As shown in Figure 7b, the angle‐dependent GIWAXS measurement reveal that the combined strategy can effectively enhance molecular crystallinity in the vertical direction, leading to improved charge transport and extraction processes. Ultimately, the optimized D18‐Cl/N3(DIB) devices afford an outstanding PCE of 18.42% owing to the improved *J*_sc_ and FF. Henceforth, additive DIB has been extensively used in OSCs, proving its exceptional efficacy in morphology control and performance improvement of devices [6, 8, 85, 86, 87, 88].

**FIGURE 7:**
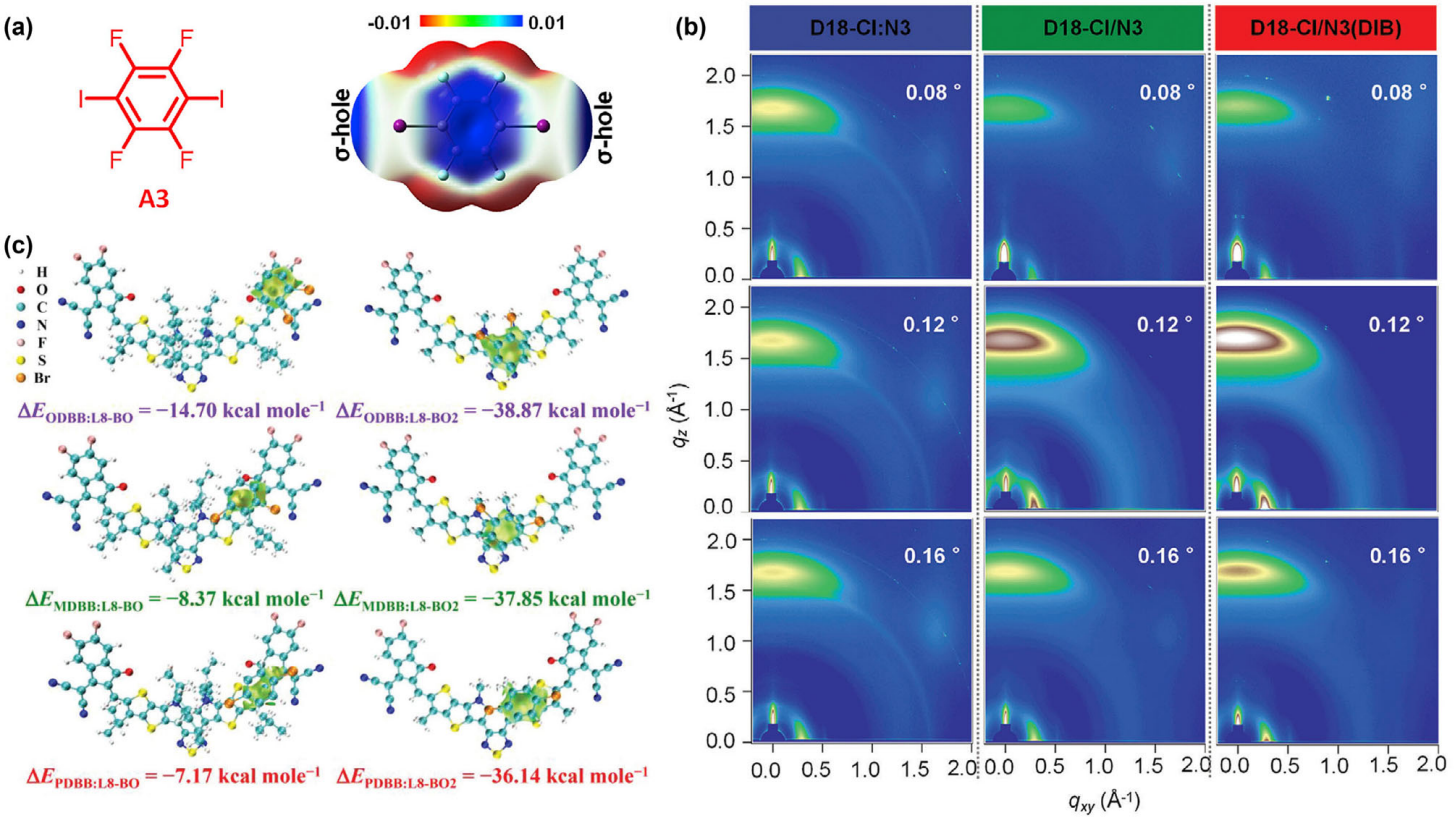
(a) Chemical structure and ESP distribution of additive A3. Reproduced with permission [40]. Copyright 2020, Elsevier. (b) 2D GIWAXS patterns of D18‐Cl:N3, D18‐Cl/N3, and D18‐Cl/N3(DIB) blend films measured at various incidence angles (0.08°, 0.12°, and 0.16°). Reproduced with permission [42]. Copyright 2022, Wiley‐VCH. (c) The non‐covalent interaction energies between non‐fullerene acceptor and three isomeric additives with the corresponding equilibrium molecular geometries. Reproduced with permission [27]. Copyright 2023, Wiley‐VCH.

In addition to the additive DIB, several structurally analogous materials have been successfully utilized as additives in OSCs. For example, in 2021, Yu et al. utilized 1,3‐diiodobenzene (1,3‐DIB) as an additive in the PM6:Y6 OSCs [43]. As expected, the devices treated with 1,3‐DIB exhibit excellent efficiency, stability and reproducibility. Subsequently, Xu et al. further demonstrated that *m*DIB (1,3‐DIB) is a promising volatile additive [89]. In 2023, Bai et al. used the low‐boiling solvent additive, 1,4‐difluorobenzene (1,4‐DFB), into the PM6:Y6:PC_61_BM OSCs fabricated via doctor blading under ambient conditions [44]. The 1,4‐DFB‐treated blend films show bicontinuous interpenetrating networks and reasonable small domains, yielding a higher PCE of 15.34% compared to the control device (14.28%). Furthermore, the 1,4‐DFB‐treated devices show superior stability, which can maintain over 90% of its initial efficiency following 55 days of storage in a glovebox. In 2023, He et al. employed the solvent additive 1,2‐dibromobenzene (DBB) to enhance the solid‐liquid phase separation, leading to ordered molecular orientation, favorable vertical distribution and enlarged domain size in blend films [45]. As a result, the DBB‐treated D18‐Cl:N3 OSCs exhibit a superior efficiency of 18.54%, outperforming the as‐cast device (17.70%) and the device processed with 1,2,3‐trichlorobenzene (17.83%). In 2023, Wang et al. proposed using the three isomers of dibromobenzene (i.e., solvent additives: ODBB (DBB) and MDBB; solid additive: PDBB) as additives to study the influence of the dibromo‐substituent positions on the morphology and device performance [27]. The results indicate that the symmetrically structured additive PDBB with the lowest steric hindrance and the weakest non‐covalent interaction with the acceptor can form superior molecular crystallinity and ideal phase separation among the three additives (Figure 7c). As a result, a champion PCE of 18.42% has been achieved in the PDBB‐treated PM6:L8‐BO device, which also provides superior stability. In 2024, Haris et al. employed a series of 1,4‐dihalobenzene solid additives (namely DClB, DBrB (PDBB) and DIB) to control the morphology of the active layer in OSCs prepared with non‐halogenated solvent (e.g., *o*‐xylene, *o*‐XY) [46]. The results demonstrate that additive DBrB can suppress the severe self‐aggregation of the acceptor during film formation and thus form optimized morphology with appropriate phase separation. Finally, the PCE of PM6:Y6‐BO OSCs has been increased from 15.1% to 15.9% (DClB), 17.0% (DBrB) and 16.1% (DIB). Meanwhile, the DBrB‐treated PM6:Y6‐HU OSCs afford an outstanding PCE of 18.6%.

Notably, heterohalogen‐substitution volatile solid and solvent additives have also demonstrated remarkable device performance. In 2023, Chen's group developed two low‐melting‐point solid additives, 1,4‐dichlorobenzene (DCB or DClB) and 1‐chloro‐4‐iodobenzene (CIB), which are fully removed during spin‐coating [24]. Notably, the DCB and CIB‐treated PM6:BTP‐eC9 OSCs exhibit a champion PCE of 18.2% and 18.4%, respectively, which are higher than that of the DIB‐treat OSCs (17.8%). Additionally, the CIB‐processed PM6:D18‐Cl:L8‐BO OSCs deliver an excellent PCE of 19.1%. Similarly, heterohalogen‐substitution solid additive 1‐bromo‐4‐iodobenzene (BIB) outperforms 1,4‐dibromobenzene (DBB' or PDBB/DBrB) and DIB [47]. Later, they further reported a new in situ removable (ISR) solid additive 1‐bromo‐4‐chlorobenzene (CBB) with a removing time between that of DCB and CIB, which enables the optimal active layer morphology, yielding a champion PCE of 18.58% in the D18:L8‐BO OSCs [48]. In 2023, Lei et al. developed an asymmetric heterohalogen‐substitution solid additive 1,2‐chloroiodobenzene (OCIB) to optimize molecular stacking of the active layer [49]. After additive OCIB treatment, the PM6:L8‐BO blend films show enhanced molecular crystallinity and more suitable phase separation, which can facilitate exciton dissociation and charge transport. Ultimately, the PCE of PM6:L8‐BO device has been substantially increased from 16.17% to 18.30% under AM 1.5G (100 mW cm^−2^) and from to 26.46% to 29.52% under indoor light (1000 lux) illumination. Moreover, similar volatile solvent additives 1‐fluoro‐2‐iodobenzene (OFIB) and 1‐chloro‐2‐bromobenzene (ODCB) can optimize the nucleation and drying kinetics of the photoactive materials, resulting in a well‐defined fibril structure in the active layer and effectively improving device performance [90, 91].

Recently, Li et al. proposed a deuterated solid additive strategy, replacing additive 4‐bromo‐iodobenzene (p‐IBrPh or BIB) with its deuterated analog (p‐IBrPh‐d_4_) in binary OSCs [50]. In this work, a systematic study was conducted to investigate the effects of additive p‐IBrPh‐d_4_ on the absorption, intermolecular interaction, morphology, exciton dynamics as well as charge transport characteristics. The findings reveal that this deuteration strategy achieves optimized morphology with compact molecular packing, resulting in enhanced charge transport and reduced energy loss. Therefore, the p‐IBrPh‐d_4_‐processed PM6:Y6 device affords a higher PCE of 17.46% compared to the control device (16.03%) and p‐IBrPh‐treated device (16.89%), which also demonstrate superior operational and thermal stability. Moreover, this deuteration strategy exhibits excellent universality in multiple binary systems, achieving an outstanding PCE of 19.32% in PM6:BTP‐eC9 OSCs.

Besides dihalobenzenes, trihalobenzenes and polyhalobenzenes have also been widely reported as volatile additives in OSCs. In 2022, Fu et al. used additive 2,6‐dichloroiodobenzene (DCIB) to optimize morphology in non‐fullerene OSCs [92]. The morphology optimization induced by additive DCIB can promote charge extraction and transport, and suppress charge recombination. Ultimately, the PCE of PM6:Y6 OSCs has been dramatically increased from 15.36% to 17.02%. In 2022, Song et al. proposed a process‐aid solid engineering by employing asymmetric configuration 1,3‐dibromo‐5‐chlorobenzene (DBCl) as additive, which can effectively fine‐tune the morphology and enhance the aggregation state of the blend films due to its asymmetric halogen distribution and high volatility [25]. Ultimately, the PM6:L8‐BO devices treated with DBCl afford a high PCE of 18.5%. Subsequently, they further applied volatile solid additive 1,3,5‐tribromobenzene (TBr) and 1,3‐dibromo‐5‐iodobenzene (DBI) to regulate the film‐forming kinetics of the photoactive layer [26, 93]. In 2023, Fu et al. reported a non‐monotonic intermediate state manipulation strategy by using additive 1,3,5‐trichlorobenzene (TCB) as crystallization regulator to optimize active layer morphology [51]. Unlike the DIO‐treated film with excessive molecular aggregation, the TCB‐treated blend film exhibits more ordered molecular packing and moderate molecular aggregation. Hence, the TCB‐treated PM6:BTP‐eC9 device offers a higher PCE of 19.31% (certified 18.93%) with a low non‐radiative recombination loss compared to the DIO‐treated devices (Figure 8a). Later, Zhao et al. employed three halogenated volatile solid additives (i.e., TCB, TBB (TBr) and 1,3,5‐triiodobenzene (TIB)) to investigate the influence of intermolecular interactions between the additive and the non‐fullerene acceptor in PM6:Y6 OSCs [52]. Their findings demonstrate that these additives can stay between two acceptor molecules and thus form halogen bonds, thereby regulating the molecular aggregation behavior of the acceptor Y6. Finally, a champion PCE of 17.9% is obtained in the TBB‐treated PM6:Y6 devices. In 2023, Wang et al. utilized highly volatile additive 3,5‐dichlorobromobenzene (DCBB) to enhance the device performance and stability of OSCs. The introduction of additive DCBB can effectively regulate the phase separation kinetics during the film‐forming process, thereby yielding an ideal morphology with a reinforced molecular packing and a favorable phase separation, which can facilitate charge transport and mitigate charge recombination. Eventually, the DCBB‐treated PBQx‐TF:eC9‐2Cl devices deliver a record PCE of 19.2% (certified 19.0%) with superior photostability (Figure 8b). More attractively, the DCBB‐processed PBQx‐TF:eC9‐2Cl module with an area of 23.6 cm^2^ fabricated by blade‐coating affords an excellent PCE of over 15.1%. Subsequently, Wu et al. used the isomer of DCBB, namely 2‐monobromo‐1,3‐dichloro‐bezene (BDCB), as an additive in devices fabricated via the LbL method [54]. Consequently, a high PCE of 19.03% is achieved in the LbL‐processed devices. In 2024, Park et al. reported a volatile solvent additive, 4‐chloro‐2‐fluoroiodobenzene (CFIB), in the D18‐Cl:N3 system, which can properly interact with the host materials, dominantly with the acceptor N3 [55]. The results indicate that additive CFIB can induce a favorable nanoscale morphology in the blend film, leading to improved charge dynamics and kinetics. Consequently, the CFIB‐treated D18‐Cl:N3 OSCs deliver a higher PCE of 18.54% compared to the CFIB‐free device (17.80%). Moreover, the CFIB‐treated devices show exceptional thermal stability (*T*_80_ = 120 h, at 85°C). Later, Xia et al. developed three isomeric additives (i.e., solvent additive: 4‐bromo‐1,2‐dichlorobenzene (LCB); solid additives: 1‐bromo‐2,4‐dichlorobenzene (SCB) and 2‐bromo‐1,4‐dichlorobenzene (VCB)) into the D18:L8‐BO blend films, which enable stepwise regulation of the acceptor's aggregation behavior during film formation [56]. Ultimately, the LCB‐, SCB‐ and VCB‐treated D18:L8‐BO devices deliver high PCE of 18.96%, 17.97% and 19.33%, respectively, significantly outperforming the control device (17.29%). In 2026, Zhao et al. applied three isomeric halogenated additives (namely 2‐chloro‐1,3‐dibromobenzene (BrClBr), 2‐chloro‐1,4‐dibromobenzene (DBrCl) and 1‐chloro‐2,4‐dibromobenzene (ClDBr)) on the aggregation behavior of acceptor L8‐BO in the PM6/L8‐BO OSCs [57]. Accordingly, the BrClBr‐, DBrCl‐ and ClDBr‐treated PM6/L8‐BO OSCs deliver high PCEs of 19.9%, 19.3% and 19.0%, respectively, far exceeding that of the as‐cast OSCs (17.4%). In 2025, Wu et al. proposed a novel solid additive 1,3,5‐tris(bromomethyl)benzene (TBMB), featuring longer side‐chain length compared to additive TBB [58]. Theoretical calculations suggest that TBMB exhibits stronger non‐covalent intermolecular interactions with the photoactive layer components when compared to TBB, thereby enhancing molecular aggregation and crystallization kinetics. Eventually, the TBMB‐treated PM6:Y6 device delivers a higher PCE of 17.92% than the TBB‐treated device (17.20%). Notably, the TBMB‐processed D18:L8‐BO device offers an exceptional PCE of 20.04%.

**FIGURE 8:**
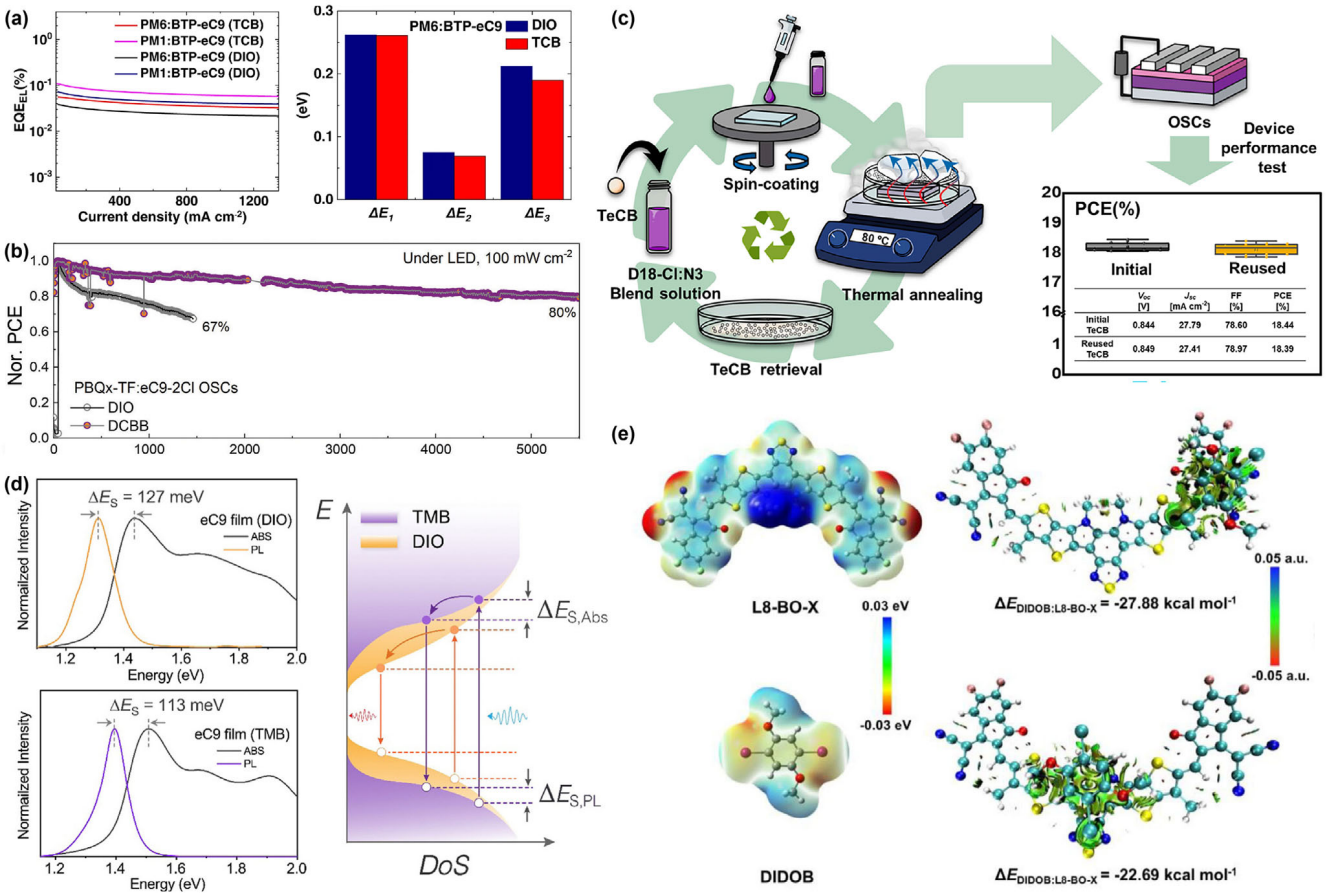
(a). EQE_EL_ of devices at various injected current densities and detailed energy loss in the DIO‐ and TCB‐treated PM6:BTP‐eC9 OSCs. Reproduced with permission [51]. Copyright 2023, Springer Nature. (b) Photostability of DIO‐ and DCBB‐processed PBQx‐TF:eC9‐2Cl OSCs. Reproduced with permission [53]. Copyright 2023, Wiley‐VCH. (c) Schematic diagram of the retrieval process of the additive TeCB in the D18‐Cl:N3 system. Reproduced with permission [32]. Copyright 2025, Elsevier. (d) Photoluminescence (PL) and absorption spectra of eC9 film treated with the additive TMB and DIO, respectively, and schematic diagram of the correlation between energetic disorder and Stokes shift in the corresponding films. Reproduced with permission [31]. Copyright 2023, Royal Society of Chemistry. (e) ESP distribution of L8‐BO‐X and DIDOB, and the non‐covalent interaction energies between DIDOB and L8‐BO‐X at different equilibrium molecular geometries. Reproduced with permission [72]. Copyright 2025, Wiley‐VCH.

In 2024, Xie et al. developed 1,2‐dibromo‐4,5‐difluorobenzene (DFB) and 1,4‐dibromotetrafluorobenzene (TFB) as additives in OSCs [59]. The two additives can simultaneously interact with both the donor and the acceptor, and thus regulate the crystallization kinetics of the blend films during the film formation process. Compared with the non‐polar TFB, polar DFB can facilitate more ordered molecular packing of the blend films owing to its stronger dipole‐dipole interactions with the photoactive layer components. Accordingly, the efficiency of PM6:Y6 device has been significantly increased from 15.12% to 17.15% after DFB treatment, which is higher than that of 1‐CN (16.13%) or TFB (16.30%). Moreover, the DFB‐treated PM6:BTP‐eC9 and PM6:L8‐BO devices also afford excellent PCE of 18.17% and 18.46%, respectively. In 2024, Haris et al. reported a solid additive 1,4‐dibromo‐2,5‐diiodobenzene (DBrDIB) in OSCs prepared with non‐halogenated solvent, which can inhibit the excessive aggregation of the non‐fullerene acceptor and optimize the phase separation due to the strong intermolecular interaction with acceptor [60]. Therefore, the efficiency of the PM6:Y6‐BO OSCs has been substantially increased from 15.6% to 17.9%. Notably, the DBrDIB‐processed PM6:Y6‐HU OSCs exhibit an excellent PCE of 19.1%. Recently, Xiong et al. introduced DBB' and 1,4‐difluoro‐2,5‐dibromobenzene (DFBB) as additives in OSCs [61]. Compared with DBB', additive DFBB exhibits higher ESP extremum and stronger σ‐hole interaction, which can form strong interactions with acceptor. As a result, the DFBB‐processed PM6:L8‐BO device offers a higher PCE of 19.2% compared to the control device (17.0%) and DBB'‐treated device (18.2%). Subsequently, Mao et al. pointed out that additive DBDF (DFBB) undergoes complete in situ volatilization during the spin‐coating process [62]. Finally, the D18:N3:Y6‐1O OSCs treated with additive DBDF afford a high PCE of 20.2 %. In 2024, Zou's group employed three isomeric additives (i.e., 1,2‐dibromo‐4,5‐difluorobenzene (*o*‐DBDF or DFB), 1,3‐dibromo‐2,5‐difluorobenzene (*m*‐DBDF) and 1,4‐dibromo‐2,5‐difluorobenzene (*p*‐DBDF or DFBB/DBDF)) to study the effect of intermolecular interactions between the polymer donor and additives on the morphology of the active layer [63]. The results show that the enhanced interaction between the additives and the electron‐withdrawing unit of polymer donor can facilitate the ordered packing of the blend film. Compared with *p*‐DBDF and *o*‐DBDF, additive *m*‐DBDF demonstrates the strongest interaction with the fluorinated quinoxaline unit of the polymer donor TPQ‐3F, leading to the best PCE for the TPQ‐3F:Y6:*m*‐DBDF OSCs. In 2025, they designed two hetero‐halogenated solid additives (i.e., 1,5‐dibromo‐2,4‐difluorobenzene (DBDF') and 1,5‐dibromo‐2,4‐diiodobenzene (DBDI)) to precisely control the intermolecular interactions between additive and active layer materials, thereby optimizing the growth kinetics of active layer [64]. In particular, the efficiency of PM6:L8‐BO OSCs has been significantly enhanced from 17.37% to 19.42% after DBDI treatment. In 2025, Kim et al. applied 1,2,3,5‐tetrachlorobenzene (TeCB) as additive to manipulate the evolution of active layer morphology [32]. The introduction of additive TeCB can simultaneously improve the molecular ordering in both the donor and acceptor phases. As a result, the D18:L8‐BO OSCs treated with TeCB show an excellent PCE of 19.16%. Interestingly, as shown in Figure 8c, they also developed a retrieval and reuse strategy for the volatile additive TeCB during OSCs fabrication. In 2025, Wang et al. employed two isomeric solid additives, 2‐bromo‐1,3,5‐trichlorobenzene (TCBB‐1) and 3,4,5‐trichlorobromobenzene (TCBB‐2), to control the morphology of the PM6:L8‐BO blend films via strong electrostatic interactions [94]. As a result, the PM6:L8‐BO OSCs treated with TCBB‐1 and TCBB‐2 afford high PCEs of 18.93% and 18.01%, respectively, which are significantly higher than that of the control device (16.09%). Subsequently, Zhang et al. compared three isomeric additives (i.e., *o*DF (liquid), *m*DF (quasi‐solid) and *p*DF (or DFBB/DBDF/*p*‐DBDF solid)) to study the influence of physical state of ISR additives on performance of OSCs [65]. Evidence suggests that the quasi‐solid *m*DF serves as a cold‐sublimating transient structural agent, which can widen the early‐stage solvent removal window and accelerate the intermediate crystallization. Finally, the PM6:L8‐BO and D18:L8‐BO devices based on *m*DF treatment afford high PCEs of 19.28% and 20.08%, respectively. Moreover, the *m*DF‐treated devices demonstrate superior operational stability (*T*_80_ = 477 h), which is higher than those of *o*DF‐ (58 h), *p*DF‐ (279 h) and as‐cast devices (103 h). The ranking of stability correlates with the physical state and the structural metrics. Specifically, additive *m*DF promotes tighter π‐π stacking and increased coherence length, and prevents issues triggered by additive residues, thereby suppressing morphological drift and nonradiative loss pathways during stress. In 2026, Yang et al. employed three isomeric molecules, namely 1‐iodo‐2,3,5‐trichlorobenzene (1TCB), 1‐iodo‐2,4,5‐trichlorobenzene (2TCB) and 3‐iodo‐1,2,4‐trichlorobenzene (4TCB), as additives to finetune the intermolecular interactions between the photoactive materials and additives [66]. These additives can effectively promote the *J*‐aggregation of the active layer components and broaden their absorption spectrum. Consequently, the 1TCB‐, 2TCB‐ and 4TCB‐processed D18:L8‐BO OSCs afford high PCEs of 20.94% (certified 20.42%), 20.38% and 19.91%, respectively. In 2023, Ran et al. conducted a comparison of five halogenated benzene‐based additives (i.e., hexabromobenzene (HBB), 1,3,5‐tribromobenzene (TBB), 1,3,5‐tribromo‐2,4,6‐trifluorobenzene (TFTB), hexafluorobenzene (HFB) and 1,3,5‐trifluorobenzene (TFB)) with different melting and boiling points [95]. Among them, additive TFTB can induce more ordered molecular stacking and optimized phase separation due to its optimal volatility and stronger intermolecular interactions with the host matrix. Ultimately, a champion PCE of 19.43% is achieved in the TFTB‐treated ternary PBTz‐F:PM6:L8‐BO OSCs. Additionally, several joint strategies based on the aforementioned additives (e.g., additive‐assisted LbL or ternary strategy [96, 97] and solid solvation assisted doping method [98]) have been reported to improve device performance.

Beyond halogenated benzenes, their derivatives substituted with alkyl, alkoxy, cyano, nitro and other groups have also been developed as volatile additives. In 2023, Chen et al. developed a novel volatile solid additive 1,3,5‐trimethoxybenzene (TMB) to restrict the energetic disorder (Figure 8d) [31]. The results indicate that the strong electrostatic interactions between TMB and the terminal groups of the non‐fullerene acceptor can facilitate the face‐to‐face packing, resulting in optimized phase separation and molecular stacking. The optimized morphology effectively reduces energetic disorder, thereby further enhancing exciton delocalization and suppressing non‐radiative recombination loss. Eventually, the TMB‐treated PBDB‐TF:eC9 and PBDB‐TF:eC9:HDO‐4Cl OSCs offer excellent PCEs of 18.61% and 19.30%, respectively, which are higher than that of the DIO‐treated OSCs. In 2025, Li et al. designed a series of halogenated volatile additives (namely 1‐fluoro(chloro/bromo/iodo)‐3,5‐dimethoxy‐benzene (F/Cl/Br/I‐DMB)) to fine‐tune the crystallization kinetics of the blend films [67]. The results demonstrate that with increasing halogen atomic weight, the promoting effect of additives on donor PM6 gradually increases, whereas that on acceptor L8‐BO gradually weakens. Ultimately, a champion PCE of 20.40% is obtained in I‐DMB‐treated OSCs with a low non‐radiative recombination energy loss of 0.189 eV owing to the well‐balanced crystallization rate and optimal morphology. Later, Li et al. employed 1‐bromo‐3,5‐dimethoxybenzene (BrDO or Br‐DMB) and 1‐chloro‐3,5‐dimethoxybenzene (ClDO or Cl‐DMB) as additives to modulate the active layer morphology, which led to markedly improved device performance [68]. In 2025, Deng et al. used additives F‐DMB and Cl‐DMB to selectively modulate donor and acceptor molecular aggregation in solution, which can effectively address crystallization discrepancies and inhibit localized defect formation [69]. As a result, the resultant D18:L8‐BO:ZH1 devices treated with Cl‐DMB afford a high PCE of 20.16% (certified 19.76%). Moreover, volatile solid additives 3,5‐dichloroanisole (DCA) and 3,5‐dibromoanisole (DBA) have also been applied to optimize the morphology of active layer [99, 100].

In 2024, Zhou et al. employed a series of additives (i.e., DIB, 1,4‐dimethoxybenzene (DOB), 1,4‐dimethylthiobenzene (DSB), 1‐iodo‐4‐methoxybenzene (OIB) and 1‐iodo‐4‐methylthiobenzene (SIB)) to optimize the blend morphology [70]. Among them, additive SIB with sulphuration‐iodination synergy enable more favorable morphology with highly ordered molecular packing. As a result, the SIB‐treated PM6:L8‐BO, PBTz‐F:L8‐BO and PM6:PBTz‐F:L8‐BO OSCs deliver remarkable PCE of 18.87%, 18.81% and 19.68%, respectively. Subsequently, Gao et al. utilized the solid additive OIB to optimize the crystallization kinetics of the active layer during the film‐forming process [71]. As a result, the efficiency of the PM6:L8‐BO devices has been increased from 17.23% to 18.58% after the introduction of additive OIB. In 2025, Guo et al. reported a solid additive 1,4‐diiodo‐2,5‐dimethoxybenzene (DIDOB) to enhance the photovoltaic performance of OSCs [72]. The study reveals that additive DIDOB can induce the formation of a more compact and highly ordered molecular packing for the blend films due to its strong intermolecular interactions with L8‐BO‐X (Figure 8e). Hence, the DIDOB‐treated PM6:L8‐BO‐X and D18:PM6:L8‐BO devices offer outstanding PCEs of 19.75% and 20.11% (certified 20.03%), respectively.

In 2024, Cui et al. developed cyanobenzene (CNB) and 1,4‐dicyanobenzene (DCNB) as additives to fine‐tune the growth process of the blend films [73]. The results reveal that both CNB and DCNB can prolong the nucleation and growth stages of the active layer, leading to enhanced molecular packing and improved domain purity. Ultimately, the CNB‐ and DCNB‐processed D18:BTP‐eC9‐4F exhibit higher PCE of 19.00% and 19.43%, respectively, compared to the control device. Notably, a PCE of 14.35% is obtained for a large‐area module with an active area of 15.84 cm^2^ prepared by blade‐coating. In 2025, Liu et al. utilized solid additive 4‐nitro‐benzonitrile (NBN) with non‐halogenated strong electron‐withdrawing groups to boost photovoltaic performance of devices [74]. The results suggest that the introduction of additive NBN can significantly enhance the optical absorption and molecular crystallinity of the blend films, resulting in superior charge transport and reduced charge recombination. As a result, the NBN‐processed D18:BTP‐eC9 and D18:L8‐BO:BTP‐eC9 OSCs fabricated by non‐halogenated solvent show excellent PCEs of 20.22% and 20.49%, respectively. Recently, Li et al. introduced 4‐iodobenzonitrile (IBZN) as additive to modulate the molecular packing and crystallization behavior of the active layer [75]. Typically, after additive IBZN treatment, the efficiency of the PM6:L8‐BO device has been increased from 17.49% to 18.77%.

In 2024, Tao's group developed a volatile solid additive 3,5‐dibromotoluene (DTL) in OSCs [76]. The findings reveal that additive DTL, which selectively interacts with the acceptor, can effectively regulate the crystallization kinetics and molecular stacking of the acceptor. Ultimately, the DTL‐processed PM6:L8‐BO OSCs show higher PCE of 18.87% and better stability compared to the DIO‐treated OSCs. Meanwhile, they also replaced a bromine atom in solid additive TBB with highly electronegative groups of trifluoromethyl or trifluoromethoxy, yielding the solvent additives 3,5‐dibromobenzotrifluoride (DBTF) and 3,5‐dibromo‐triffluoromethoxybenzene (DBOF) [77]. All additives can form π–π interactions with the acceptor Y6, among which additive DBOF exhibits the strongest interaction, thereby effectively modulating molecular packing. Ultimately, the DBOF‐treated D18:Y6 OSCs afford a higher PCE of 18.78% compared to the as‐cast device (17.54%) and the devices processed with TBB (18.00%) or DBTF (18.28%). Furthermore, they combined volatile additive 3,5‐dichloro‐2,4,6‐trifluorobenzotrifluoride (DTBF) with the LbL processing method to achieve high‐performance OSCs [78]. As a result, a champion PCE of 19.00% is obtained in the DTBF‐treated D18‐Cl/BTP‐4F OSCs. In 2025, Chen et al. employed two volatile isomerization additives (namely, solvent additive: 2‐bromo‐5‐iodo‐1,3‐dimethylbenzene (2‐BrIDB), and solid additive: 5‐bromo‐2‐iodo‐1,3‐dimethylbenzene (5‐BrIDB)) in LbL‐assembled OSCs to fine‐tune blend morphology [79]. Both 2‐BrIDB and 5‐BrIDB can facilitate ordered molecular stacking of acceptor L8‐BO. Notably, solvent additive 2‐BrIDB show stronger non‐covalent interaction with acceptor L8‐BO, and can partially dissolve the underlying D18 films. Therefore, the introduction of additive 2‐BrIDB can prolong the film‐forming time and promote intimate interdiffusion between donor D18 and acceptor L8‐BO, leading to optimal vertical phase separation. Ultimately, the D18/L8‐BO OSCs processed with 2‐BrIDB and 5‐BrIDB deliver high PCEs of 20.81% and 19.43%, respectively, far surpassing the as‐cast devices (18.53%).

In addition, compounds containing carbonyl and ester groups have been developed as volatile additives to improve device performance in OSCs. As early as 2021, Liu et al. developed a volatile and halogen‐free solvent additive, ethyl 2‐hydroxybenzoate (EHB), into the PTB7‐Th:PC_71_BM OSCs [80]. The results demonstrate that the introduction of additive EHB can induce improved molecular packing and phase separation in the photoactive layer, thereby facilitating charge extraction and suppressing bimolecular recombination. As a result, the EHB‐treated device affords a higher PCE of 9.64%, outperforming the DIO‐treated device (8.75%) and control device (5.27%). In 2023, Chen et al. employed volatilizable benzoylacetate (BA) as additive to enhance the performance of OSCs [81]. The results indicate that the introduction of additive BA leads to optimized morphology of the active layer with suitable phase separation and enhanced molecular crystallinity. Consequently, the PCE of PM6:Y6 OSCs has been significantly increased from 15.2% to 17.4%, which is higher than that of the 1‐CN‐treated OSCs (16.4%). In 2025, Li et al. developed three non‐halogenated solid additives (i.e., phthalic anhydride (PA), benzo[*c*]thiophene‐1,3‐dione (S‐PA) and benzo[*c*]selenophene‐1,3‐dione (Se‐PA)) in PM6:L8‐BO OSCs [82]. The results indicate that the three additives with strong volatility and high crystallinity exhibit different dipole moments, which further influences the intermolecular interactions between the additives and host materials, thereby altering their aggregation behavior. Among them, additive S‐PA shows a moderate dipole moment, leading to more appropriate intermolecular interactions with the active layer materials and more favorable morphology. Consequently, the S‐PA‐treated PM6:L8‐BO devices afford a champion PCE of 18.51%, which is higher than those of the devices processed with PA or Se‐PA (18.20% and 17.95%, respectively) and the as‐cast devices (17.20%). In 2025, Jiang et al. designed and synthesized two coumarin‐derived additives, namely 7‐(diethylamino)‐2*H*‐chromen‐2‐one (C5) and 3‐(benzo[*d*]thiazol‐2‐yl)‐7‐(diethylamino)‐2*H*‐chromen‐2‐one (C6, non‐volatile additive), which can optimize the active layer morphology, improve charge transfer, and reduce energy loss [83]. As a result, the C5‐ and C6‐processed D18:L8‐BO OSCs deliver outstanding PCEs of 19.55% and 20.32%, respectively, outperforming the additive‐free device (18.85%).

In summary, structurally simple MAHs derivatives (i.e., dihalobenzenes, trihalobenzenes, polyhalobenzenes and their derivatives substituted with alkyl, alkoxy, cyano and nitro groups) have been extensively reported as volatile additives in non‐fullerene OSCs. In these studies, the additive structure, active layer morphology and charge carrier dynamic processes have been systematically investigated. The results indicate that the MAHs derivatives additives exhibit strong non‐covalent intermolecular interactions (such as halogen bonds, π‐π stacking and dipole‐dipole interactions) with the photoactive layer components, thereby effectively improving the molecular packing and crystallization kinetics of the active layer, and consequently enhancing the charge carrier transport characteristics and carrier dynamic processes. Ultimately, the introduction of MAHs derivatives as additives leads to a significant improvement in the performance of OSCs. Notably, the development of MAHs derivatives as additives has also made a monumental contribution to the creation of high‐efficiency OSCs.

#### 2.1.3 Polycyclic Aromatic Hydrocarbons (PAHs) Derivatives

Recent studies indicate that certain PAHs and their derivatives can act as volatile additives in OSCs. As shown in Figure 9, they can be further classified into fused ring PAHs derivatives and non‐fused ring PAHs derivatives according to structural differences.

**FIGURE 9:**
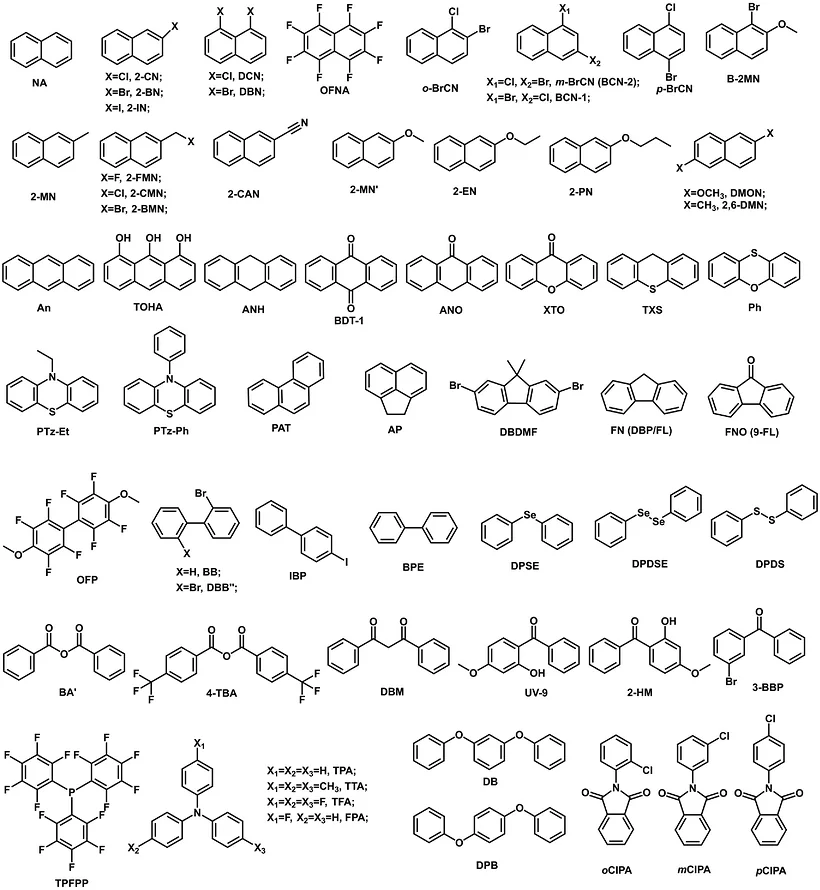
Chemical structures of volatile additives based on PAHs derivatives.

##### 2.1.3.1 Fused Ring PAHs Derivatives

Among them, commercially available, low‐cost fused ring PAHs and their derivatives (e.g., anthracene, phenanthrene, naphthalene, and fluorene) are good candidates as volatile additives for optimizing molecular aggregation behavior and film formation kinetics of the photoactive layer, due to their unique crystallinity and high volatility [22, 101]. As listed in Table 3, such additives can effectively improve the photovoltaic performance of OSCs.

**TABLE 3:** Summary of photovoltaic parameters for devices processed with volatile additives based on fused ring PAHs derivatives.

Active layer	Additive	*V*_oc_ [V]	*J*_sc_ [mA cm^−2^]	FF [%]	PCE [%]	Refs.
PM6:Y6	w/o	0.863	24.93	70.9	15.30	[21]
	1‐CN	0.832	25.60	73.5	15.60	
	An	0.844	25.91	77.8	17.02	
D18‐Cl:N3	w/o	0.854	26.58	75.77	17.15	[102]
	DPE	0.848	27.05	75.45	17.39	
	TOHA	0.862	27.29	76.20	17.91	
PM6:Y6 (CB)	w/o	0.800	24.09	70.8	13.65	[101]
	PAT	0.829	27.24	78.5	17.71	
PM6:Y6 (TOL)	w/o	0.780	19.30	61.0	9.18	
	PAT	0.822	27.66	79.2	17.99	
PM6:Y6 (*o*‐XY)	w/o	0.778	22.63	69.0	12.16	
	PAT	0.837	28.28	80.5	19.04	
PM6:Y6	w/o	0.752	16.2	68.8	8.36	[103]
	PAT	0.802	25.6	78.2	16.1	
PM6:Y6:PC_71_BM	PAT	0.814	25.5	78.9	16.4	
PM1/L8‐BO‐X	w/o	0.92	26.9	77.1	19.2	[104]
	AP	0.91	27.9	82.3	21.0	
	AP	0.91	27.8	80.9	20.5^a^	
PM6:Y6	w/o	0.848	25.96	70.0	15.41	[105]
	FN	0.836	26.64	70.5	15.71	
	ANH	0.832	26.91	71.7	16.05	
	XTO	0.828	27.36	75.6	17.12	
	FNO	0.823	27.62	75.8	17.23	
	TXS	0.839	27.75	75.5	17.56	
D18:N3:AQx‐2F	w/o	0.869	27.85	78.5	19.00	
	TXS	0.862	28.61	81.3	20.04	
D18‐Cl:N3	w/o	0.866	27.09	74.73	17.54	[106]
	Ph	0.863	27.50	77.82	18.47	
PM6:Y6	1‐CN	0.84	25.25	75.97	16.07	[22]
	NA	0.83	25.31	78.42	16.52	
D18‐Cl:N3	w/o	0.865	26.86	74.34	17.27	[107]
	OFNA	0.857	27.53	78.85	18.59	
Tz6T:eC9‐4F	w/o	0.860	25.75	67.2	14.9	[108]
	DCN	0.8632	26.12	71.0	16.0	
D18:L8‐BO	w/o	0.916	24.51	78.57	17.63	[109]
	DBN	0.908	26.45	79.94	19.20	
D18‐Fu:L8‐BO	w/o	0.887	24.9	78.5	17.4	[110]
	1‐CN	0.861	25.9	79.6	17.8	
	2‐CN	0.886	26.0	79.8	18.4	
D18/3‐ClTh	w/o	0.851	26.33	78.58	17.61	[111]
	2‐CN	0.852	27.54	79.10	18.56	
D18/3‐ClTh:BTP‐Th	2‐CN	0.860	28.16	79.05	19.15	
PM6:L8‐BO	w/o	0.893	25.89	74.3	17.17	[112]
	1‐CN	0.872	25.77	77.0	17.30	
	*o*‐BrCN	0.879	26.12	77.9	17.89	
	*m*‐BrCN	0.879	25.55	77.4	17.39	
	*p*‐BrCN	0.881	26.35	78.3	18.18	
PM6:L8‐BO	w/o	0.893	26.31	74.49	17.46	[113]
	BCN‐1	0.873	27.74	79.11	19.10	
	BCN‐2	0.878	27.93	80.42	19.72	
D18:BTP‐eC9	w/o	0.858	28.11	79.11	19.09	[114]
	2‐MN	0.864	28.27	80.24	19.60	
	2‐FMN	0.857	28.60	80.10	19.63	
	2‐CMN	0.853	28.63	80.92	19.78	
	2‐BMN	0.861	28.71	81.01	20.02	
PBDB‐T:TTC8‐O1‐4F	w/o	0.842	21.93	58.2	10.75	[115]
	DIO	0.814	20.73	71.4	12.05	
	DMON	0.834	22.63	59.3	11.19	
	DIO&DMON	0.816	23.04	70.3	13.22	
PM6:PY‐DT	DIO	0.955	23.17	65.4	14.47	[116]
	1‐CN	0.951	23.54	74.2	16.61	
	2‐MN'	0.951	23.84	76.4	17.32	
	2‐MN'	0.951	23.47	75.8	16.9^a^	
PM6:PY‐DT‐X	w/o	0.958	24.83	65.6	15.60	[117]
	2‐MN'	0.951	25.45	77.3	18.71	
	2‐EN	0.953	25.87	79.1	19.50	
	2‐PN	0.952	25.70	78.9	19.30	
	2‐EN	0.951	25.69	78.1	19.1^a^	
PM6:L8‐BO	w/o	0.908	26.06	74.54	17.43	[118]
	NA	0.886	26.12	78.55	18.18	
	2‐CAN	0.892	27.00	79.21	19.08	
PM6:L8‐BO	DIO	0.893	25.88	79.75	18.43	[119]
	DCBB	0.881	26.58	79.71	18.67	
	DBCl	0.884	26.43	80.38	18.78	
	DBT	0.889	26.52	80.67	19.02	
	3‐BBP	0.878	27.05	80.72	19.17	
	B‐2MN	0.881	27.14	81.68	19.53	
	DBM	0.878	27.17	81.07	19.34	
PM6:D18:L8‐BO	B‐2MN	0.900	27.77	82.04	20.50	
PM6:BTP‐BO‐4F	w/o	0.81	26.71	71.35	15.43	[120]
	DBDMF	0.83	26.92	77.11	17.20	
PM6:BTP‐eC9 (spin‐coating)	w/o	0.847	26.79	73.5	16.7	[121]
	9‐FL	0.851	27.65	79.0	18.6	
PM6:BTP‐eC9 (blade‐coating)	w/o	0.843	25.89	74.6	16.3	
	9‐FL	0.845	26.57	79.6	17.9	
PM6:TPT10	DIO	0.877	22.40	68.60	13.55	[122]
	BDT‐1	0.899	24.80	73.00	16.26	
	BDT‐2	0.877	24.90	69.50	15.18	
PM6:Y6	w/o	0.83	25.16	70.0	14.57	[123]
	PTz‐Ph	0.81	25.29	72.2	15.07	
	PTz‐Et	0.81	26.05	74.6	15.75	

^a^ Certified result.

In 2021, Fan et al. volatilizable anthracene (An) as solid additive to enhance the photovoltaic performance of OSCs [21]. As shown in Figure 10a, additive An can blend well with acceptor Y6 due to their high miscibility, consequently preventing excessive molecular aggregation of Y6 during film deposition. During thermal treatment, the evolving removal kinetics of an molecules create voids in the Y6 matrix that are subsequently occupied by PM6 chains, leading to optimal phase separation and well‐defined bicontinuous interpenetrating networks that ensure highly efficient exciton dissociation and charge transport. Consequently, the An‐processed PM6:Y6 devices afford a remarkable PCE of 17.02%, which is obviously higher than that of the control device (15.30%) or 1‐CN‐treated device (15.60%). In 2023, Kang et al. employed a novel solid additive 1,8,9‐Trihydroxyanthracene (TOHA) to improve the photovoltaic performance and stability of OSCs [102]. The introduction of additive TOHA induced favorable morphology with enhanced molecular packing and more refined phase separation in D18‐Cl:N3 blend films, yielding a higher PCE of 17.91% compared to the as‐cast device (17.15%) and the diphenyl ether (DPE)‐treated device (17.39%). In 2025, Fan et al. further used phenanthrene (PAT) as additive to modulate the aggregation of non‐fullerene acceptor Y6, which can effectively suppress excessive self‐aggregation of this acceptor during film formation in high‐boiling‐point solvents [101]. Notably, the PAT‐treated PM6:Y6 OSCs prepared with chlorobenzene, toluene and *o*‐XY exhibit remarkable PCEs of 17.71%, 17.99% and 19.04%, respectively. Furthermore, Deng et al. developed a spontaneous spreading (SS) process that employed solid additive PAT to regulate interfacial tension, facilitating the formation of uniform blend film on the water surface (Figure 10b) [103]. Ultimately, the as‐prepared devices show a champion PCE of 16.4%. In 2025, Fu et al. controlled the crystallization kinetics of non‐fullerene acceptor by using the crystallization‐regulating agent of acenaphthene (AP) [104]. They point out that AP optimizes the molecular packing of the acceptor through a two‐step crystallization process, wherein it first fixes the packing motif of the non‐fullerene acceptor and then refines the crystallized framework, yielding highly oriented acceptors in the blend films. In this study, in situ GIWAXS measurement was employed to track the spin‐coating and thermal annealing processes of the active layer, with and without the additive AP. Results reveal that, in contrast to the control film (virtually unchanged), the AP‐treated blend film exhibits a weakening of the diffraction rings of the additive AP during spin‑coating, indicating that the additive AP was partially removed during this process. Additionally, the blend film shows an interesting crystalline transition during thermal annealing, which can be mainly attributed to the rearrangement of the non‐fullerene acceptor. Consequently, the AP‐treated PM1/L8‐BO‐X OSCs deliver a remarkable PCE of 21.0% (certified 20.5%). Recently, Xie et al. further demonstrated the powerful capability of additive AP in morphology control and performance enhancement [124].

**FIGURE 10:**
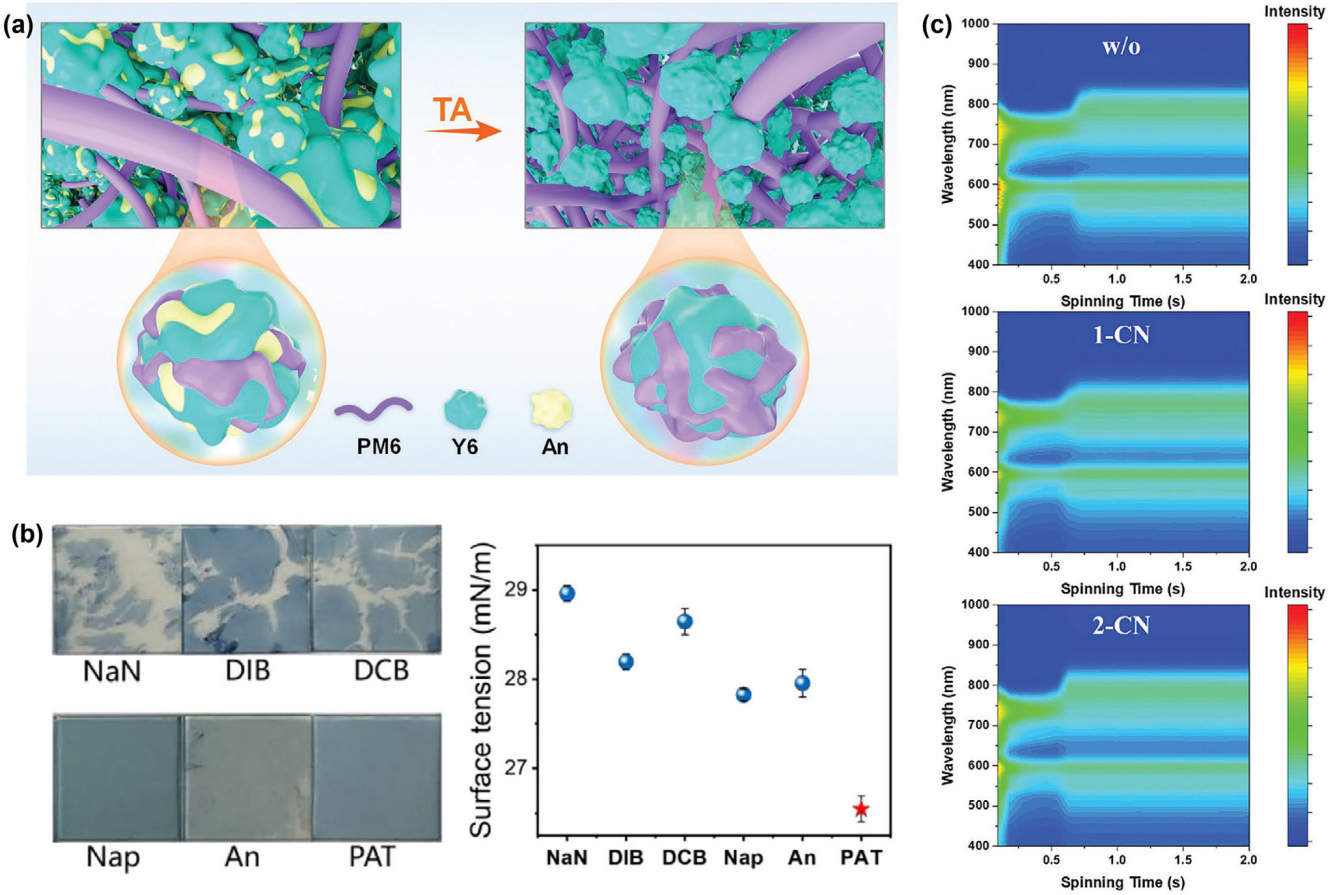
(a) Diagram depicting the impact of additive An on the morphology of PM6:Y6 blend film. Reproduced with permission [21]. Copyright 2021, Wiley‐VCH. (b) Photographs of transferred SS‐PM6:Y6 films treated with different solid additives, and surface tension of the solid additive solutions in *o*‐XY. Reproduced with permission [103]. Copyright 2025, Wiley‐VCH. (c). In situ UV–vis absorption spectra of the D18‐Fu:L8‐BO blend films with and without additive (1‐CN or 2‐CN) treatment. Reproduced with permission [110]. Copyright 2023, Wiley‐VCH.

In 2025, Li et al. used a series of solid additives (fluorene (FN), 9,10‐dihydroanthracene (ANH), 9‐fluorenone (FNO), xanthone (XTO), anthrone (ANO) and thioxanthene (TXS)) to elucidate the role of intermolecular interactions in controlling the molecular packing of acceptor [105]. Among them, the additives (i.e., FNO, XTO, ANO and TXS) containing heteroatom (oxygen or sulfur atom) exhibit strong intermolecular interactions with acceptor Y6, thereby inducing acceptor Y6 to form compact and ordered molecular stacking. In contrast, additives FN and ANH show little effect on regulating molecular stacking of Y6 due to weak intermolecular interactions with Y6. Consequently, the FNO‐, XTO‐, ANO‐ or TXS‐treated PM6:Y6 OSCs afford significantly improved PCEs. Particularly, the TXS‐processed D18:N3:AQx‐2F devices deliver an excellent PCE of 20.04%. Previously, Zhong et al. reported a novel solid additive phenoxathiin (Ph) in D18‐Cl:N3 OSCs to investigate its influence on morphology formation [106]. The results reveal that additive Ph exhibits high miscibility with the acceptor N3, effectively controlling aggregation of acceptor N3 during film formation. After additive Ph treatment, the efficiency of D18‐Cl:N3 OSCs is significantly improved from 17.54% to 18.47%.

In addition, naphthalene and its derivatives are also effective volatile additives [125]. In 2022, Zhong et al. used commercial material naphthalene (NA) as a thermal‐annealing‐free volatile solid additive in PM6:Y6 OSCs, which can restrict over‐aggregation of both donor and acceptor [22]. As a result, compared with the 1‐CN‐treated devices, the NA‐treated device shows a higher PCE of 16.52% and better photostability. Subsequently, they introduced a fluorinated NA analogy (namely octafluoronaphthalene (OFNA)) as a solid additive in D18‐Cl:N3 OSCs [107]. Ultimately, the OFNA‐treated devices without thermal annealing afford a champion PCE of 18.59%, outperforming the as‐cast device (17.27%). In 2022, Hu et al. applied 1,8‐dichloronaphthalene (DCN) as additive to improve efficiency and stability of oligothiophene‐based all‐small‐molecule OSCs [108]. Obviously, the DCN‐treated blend films show enhanced molecular crystallinity, leading to improved exciton dissociation and suppressed non‐geminate recombination. Consequently, the DCN‐processed Tz6T:eC9‐4F device exhibits a high PCE of 16.0% and remarkable long‐term stability, retaining over 90% of its initial efficiency after 3000 h of storage. In 2025, Lin et al. employed volatile solid additive 1,8‐dibromonaphthalene (DBN) to the control morphology of D18:L8‐BO blend film [109]. As a result, the DBN‐treated blend film shows more ordered molecular packing and suitable phase separation, yielding an improved PCE from 17.63% to 19.20%. In 2023, Chen et al. reported a solid additive 2‐chloronaphthalene (2‐CN), which is the isomer of additive 1‐CN, to regulate the film morphology in D18‐Fu:L8‐BO OSCs [110]. The results suggest that both 1‐CN and 2‐CN can increase the dipole moment of acceptor L8‐BO and enhance molecular aggregation. Moreover, compared with 1‐CN, additive 2‐CN can induce more rapid nucleation and crystallization of acceptor L8‐BO (Figure 10c). The 2‐CN‐treated blend film exhibits more favorable phase separation and vertical phase distribution compared to the 1‐CN‐treated and untreated counterparts. Ultimately, the 2‐CN‐treated device exhibits a higher PCE of 18.4% compared to the as‐cast device (17.4%) and 1‐CN‐treated device (17.8%). Meanwhile, Fan et al. further pointed out that additive 2‐CN melts into a liquid state before complete volatilization, which further promotes the vertical distribution of components during evaporation [111]. As a result, the 2‐CN‐processed OSCs with reduced acceptor accumulation at the bottom afford a high PCE 19.2%. In 2024, Liu et al. reported a brominated isomerization engineering by employing 1‐CN derivatives as solid additives, namely 2‐bromo‐1‐chloronaphthalene (*o*‐BrCN), 3‐bromo‐1‐chloronaphthalene (*m*‐BrCN) and 4‐bromo‐1‐chloronaphthalene (*p*‐BrCN) [112]. Among them, *p*‐BrCN has a small dipole moment that facilitates extraordinary non‐covalent interactions with both donor and acceptor. Consequently, the *p*‐BrCN‐processed PM6:L8‐BO blend film exhibits better molecular stacking and phase separation, leading to enhanced exciton dissociation and charge transport. Ultimately, the *p*‐BrCN‐treated PM6:L8‐BO device affords a higher PCE of 18.18% than those treated with *o*‐BrCN (17.89%) and *m*‐BrCN (17.39%). Subsequently, Abbasi et al. demonstrated that additive 1‐chloro‐3‐bromonaphthalene (BCN‐2 or *m*‐BrCN) could suppress high‐frequency lattice vibrations, thereby minimizing electron‐phonon scattering and promoting exciton diffusion [113]. As a result, the PM6:L8‐BO OSCs treated with BCN‐2 deliver a high PCE of 19.72%, outperforming the as‐cast device (17.46%) and the device treated with additive 1‐bromo‐3‐chloronaphthalene (BCN‐1) (19.10%). In 2025, Li et al. reported three side‐chain halogenated solid additives (namely, 2‐(fluoromethyl)naphthalene (2‐FMN), 2‐(chloromethyl)naphthalene (2‐CMN) and 2‐(bromomethyl)naphthalene (2‐BMN)) via halogen substitution on 2‐methylnaphthalene (2‐MN) in OSCs [114]. Compared with 2‐MN, the halogenated additives display enhanced non‐covalent interactions with acceptor BTP‐eC9, acting as a ‘temporary molecular bridge’ to improve molecular packing. Consequently, the 2‐BMN‐treated D18:BTP‐eC9 device delivers a remarkable PCE of 20.02%, outperforming both the as‐cast device (19.09%) and 2‐MN‐treated counterpart (19.60%).

Apart from the halogenated naphthalene derivatives discussed above, alkoxy‐, cyano‐, and methyl‐substituted naphthalene have been successfully developed as solid additives. As early as 2020, Ding et al. adopted a dual‐additive strategy by combining additives DIO and 2,6‐dimethoxynaphthalene (DMON) to fine‐tune the morphology in PBDB‐T:TTC8‐O1‐4F OSCs [115]. The results suggest that additive DIO can induce ordered molecular packing of the acceptor, whereas additive DMON can suppress over‐aggregation of both donor and acceptor. Consequently, the dual‐additive‐treated OSCs deliver a higher PCE of 13.22% compared to the single‐additive systems (DIO: 12.05% and DMON: 11.19%). In 2022, Song et al. reported a volatile solid additive 2‐methoxynaphthalene (2‐MN') in PM6:PY‐DT‐based all‐PSCs [116]. The results reveal that additive 2‐MN' can regulate the aggregation of donor PM6 and acceptor PY‐DT during the film‐forming and thermal annealing stages, leading to optimized morphology with favorable phase separation and highly ordered molecular packing. Ultimately, the PM6:PY‐DT OSCs processed with 2‐MN' offer an excellent PCE of 17.32% (certified 16.9%), which is higher than those of the control device (14.47%) and 1‐CN‐treated device (16.61%). Subsequently, they developed two novel additives 2‐ethoxynaphthalene (2‐EN) and 2‐propoxynaphthalene (2‐PN) by modifying the alkoxy chains of the additive 2‐MN' [117]. Thus, both intermolecular interactions and molecular miscibility between the additives and host materials can be controlled, resulting in optimized molecular aggregation and improved molecular crystallinity. Finally, the 2‐EN‐treated PM6:PY‐DT‐X device shows an impressive PCE of 19.50% (certified 19.1%) and outstanding photostability (*T*_80_ = 1750 h). In 2024, Xu et al. utilized the 2‐naphthonitrile (2‐CAN) as an additive to regulate morphological evolution in blend films during the drying process [118]. The findings indicate that additive 2‐CAN can facilitate the formation of highly ordered molecular packing in the blend film due to its interactions with the acceptor. In contrast, the highly volatile additive NA shows a relatively weaker regulatory capacity. As a result, the 2‐CAN‐treated PM6:L8‐BO device offers a higher PCE of 19.08% compared to the as‐cast device (17.43%) and NA‐treated device (18.18%). In 2025, Xu et al. proposed a physical selection framework developed by adding different volatile solid additives (DCBB, DBCl, 3‐bromobenzophenone (3‐BBP) and 1‐bromo‐2‐methoxynapthalene (B‐2MN), *etc*.) into sequentially deposited OSCs [119]. The findings demonstrate that the introduction of additive B‐2MN can effectively increase donor‐acceptor interfacial contacts and optimize dual‐fibril network morphology, resulting in increased exciton dissociation, suppressed exciton‐vibration coupling and extended exciton diffusion length. Consequently, the PM6:L8‐BO and PM6:D18:L8‐BO OSCs treated with B‐2MN deliver enhanced PCEs of 19.53% and 20.50%, respectively. In 2026, Song et al. employed 2,6‐dimethylnaphthalene (2,6‐DMN) as a volatile solid additive to regulate the blend morphology, thereby mitigating the dependence of device performance on the choice of solvent [126]. The results demonstrate that 2,6‐DMN, serving as a stage‐specific processing regulator, modulates the acceptor by suppressing molecular aggregation during spin‐coating and subsequently facilitating its ordered self‐assembly in the donor matrix during thermal annealing. As a result, an impressive efficiency of 20.86% is obtained in the 2,6‐DMN‐treated PM6:D18:L8‐BO‐X OSCs prepared using *o*‐XY, breaking the efficiency record for OSCs fabricated using non‐halogenated solvents.

Additionally, numerous studies have shown that fluorene, as a volatile solid additive, performs well in morphology control and performance enhancement [23, 105, 127]. Moreover, Huang et al. discovered a novel volatile solid additive 2,7‐dibromo‐9,9‐dimethylfluorene (DBDMF) for non‐fullerene OSCs [120]. The findings confirm that additive DBDMF can inhibit over‐aggregation of the non‐fullerene acceptor and enhance molecular rearrangement after thermal annealing, owing to its excellent miscibility with the acceptor. Finally, the efficiency of PM6:BTP‐BO‐4F OSCs is dramatically improved from 15.43% to 17.20% after DBDMF treatment. In 2024, Xu et al. employed 9‐fluorenone (9‐FL or FNO) as solid additive in OSCs fabricated with *o*‐XY, which could regulate the molecular assembly of both donor PM6 and acceptor BTP‐eC9 throughout both film deposition and thermal annealing processes [121]. Ultimately, the PM6:BTP‐eC9 devices afford superior PCE of 18.6% and 17.9% via the spin‐coating and blade‐coating method, respectively. Importantly, additive 9‐FL displays excellent universality in multiple systems.

Previously, Zhang et al. reported two quinone‐based solid additives, 9,10‐anthracenedione (BDT‐1) and benzo[1,2‐*b*:4,5‐*b*']dithiophene‐4,8‐dione (BDT‐2), to optimize the blend morphology in non‐fullerene OSCs [122]. The introduction of BDT‐1 or BDT‐2 leads to optimized blend morphology, increased absorption intensity, improved charge mobility and reduced bimolecular recombination. Finally, the PM6:TPT10 OSCs processed with BDT‐1 and BDT‐2 exhibit high PCEs of 16.26% and 15.18%, respectively, which is significantly higher than that of the DIO‐treated device (13.55%). Furthermore, solid additives BDT‐1 and BDT‐2 contribute to enhancing the thermal and photo‐induced stability of the devices. In 2025, Lin et al. employed phenothiazine (PTz) derivatives (i.e., ethyl (PTz‐Et) and phenyl (PTz‐Ph)) as solid additives into the PM6:Y6 system [123]. Among them, additive PTz‐Et with stronger crystallinity can induce enhanced intermolecular interactions and suppressed aggregation in the related blend films. Consequently, the PM6:Y6 OSCs treated with PTz‐Et afford a higher PCE of 15.75% compared to the as‐cast device (14.57%) and PTz‐Ph‐treated device (15.07%).

##### 2.1.3.2 Non‐Fused Ring PAHs Derivatives

Besides fused ring PAHs derivatives, non‐fused ring PAHs derivatives can also be used as volatile solid and solvent additives to optimize the morphology of photoactive layer, leading to greatly improved photovoltaic performance (Table 4). In 2021, Fan et al. applied volatile solid additive 4,4'‐dimethoxyoctafluorobiphenyl (OFP) to fine‐tune the morphology of active layer, which can effectively restrict the excessive aggregation of non‐fullerene acceptors during film deposition [128]. As exhibited in Figure 11a, the synergistic effect of OFP and 1‐CN dual‐additives can more effectively optimize morphology of the active layer compared to the conventional solvent additive. Ultimately, the PTQ10:*m*‐BTP‐PhC6 devices treated with the dual‐additives (OFP and 1‐CN) afford a higher PCE of 17.74% compared to the 1‐CN‐treated devices (16.45%). In 2025, Yu et al. employed 2‐bromobiphenyl (BB) and 2,2'‐dibromobiphenyl (DBB'') as additives [129]. The results suggest that additives BB and DBB'' with 3D structures can promote molecular packing and provide sufficient room for the self‐reassembly of the photoactive layer components during thermal annealing, yielding an optimized morphology with favorable phase separation and enhanced molecular crystallinity. Ultimately, the BB‐ and DBB''‐processed PM6:BTP‐eC9 OSCs exhibit improved PCE of 18.07% and 19.03%, respectively, compared to the as‐cast device (17.06%). In 2025, Zhang et al. developed 4‐iodobiphenyl (IBP) as additive to optimize the morphology of the active layer and improve device efficiency [130]. The results indicate that the introduction of IBP can promote the recrystallization and ordered molecular alignment of the acceptor. Hence, the IBP‐processed PM6:A4T‐16 OSCs show a PCE of 14.98%, outperforming the DIB‐treated (11.97%) and additive‐free (10.87%) devices. Moreover, the IBP‐treated PM6:BTP‐eC9 OSCs afford a high PCE of 18.81%, which can still maintain at around 17% even with a thickness greater than 360 nm. Recently, Cui and Kan et al. have demonstrated that the solid additive biphenyl (BPE) can effectively regulate the phase separation of the active layer and enhance device performance [131, 132, 133].

**TABLE 4:** Summary of photovoltaic parameters for devices processed with volatile additives based on non‐fused ring PAHs derivatives.

Active layer	Additive	*V*_oc_ [V]	*J*_sc_ [mA cm^−2^]	FF [%]	PCE [%]	Refs.
PTQ10:*m*‐BTP‐PhC6	w/o	0.870	23.72	75.9	15.66	[128]
	1‐CN	0.856	25.66	74.9	16.45	
	1‐CN&OFP	0.841	26.95	78.3	17.74	
PM6: BTP‐eC9	w/o	0.871	26.57	73.70	17.06	[129]
	BB	0.855	27.23	77.57	18.07	
	DBB''	0.861	27.87	79.32	19.03	
	DBB''^b^	0.860	28.78	79.20	19.57	
PM6:A4T‐16	w/o	0.904	20.74	58.0	10.87	[130]
	DIB	0.908	20.70	63.7	11.97	
	IBP	0.905	21.81	75.9	14.98	
PM6:BTP‐eC9	w/o	0.859	27.19	74.7	17.45	
	IBP	0.853	27.81	79.3	18.81	
PM6:Y6	w/o	0.838	26.12	73.30	16.00	[134]
	1‐CN	0.839	26.17	75.37	16.52	
	uv‐9	0.813	27.68	76.26	17.18	
PM6:L8‐BO	w/o	0.889	24.63	74.7	16.34	[135]
	1‐CN	0.850	26.06	77.6	17.19	
	2‐HM	0.875	27.13	79.4	18.85	
PM6:BTP‐eC9	w/o	0.82	24.2	74	15.10	[136]
	TPFPP	0.80	25.7	80	16.90	
PM6:BTP‐eC9:L8‐BO	w/o	0.889	26.24	76.43	17.8	[137]
	FPA	0.884	26.96	80.45	19.2	
PM6:Y6	w/o	0.856	25.9	70.0	15.5	[138]
	TA	0.846	26.1	74.8	16.5	
	DBM	0.845	27.2	74.7	17.2	
	DBM&TA	0.830	28.7	78.1	18.7	
D18:L8‐BO:BTP‐S10	w/o	0.933	25.7	77.0	18.4	[139]
	DIO&DBM	0.921	27.9	81.5	20.9	
	DIO&DBM	0.917	28.22	79.40	20.56^a^	
D18:L8‐BO (blend casting)	DBM& DCBB	0.927	26.2	78.2	18.9	[140]
D18/L8‐BO (LbL)	D(DBM)/ A(DCBB)	0.927	27.2	80.1	20.2	
PM6:Y6	w/o	0.86	26.70	72.45	16.57	[141]
	1‐CN&BA'	0.85	28.61	75.49	18.27	
PM6:L8‐BO	w/o	0.92	25.72	71.90	17.01	
	1‐CN&BA'	0.91	27.70	78.14	19.59	
PM6/L8‐BO	w/o	0.900	24.71	76.65	17.05	[142]
	4‐TBA (D+/A+)	0.907	26.65	76.51	18.49	
PM6:Y6	w/o	0.886	25.32	69.8	15.54	[143]
	DPB	0.836	27.42	76.1	17.45	
D18‐Cl:N3	w/o	0.875	26.66	73.8	17.23	
	DPB	0.859	27.52	76.8	18.15	
	DPB^b^	0.861	28.48	77.6	19.04	
PM6/L8‐BO	w/o	0.896	25.9	74.7	17.3	[144]
	DBrS	0.888	26.6	78.7	18.6	
	DPSE	0.869	26.7	79.0	18.4	
	DPDSE	0.855	26.8	79.1	18.1	
	DBrS^b^	0.885	27.3	80.8	19.5	
	DPSE^b^	0.865	27.6	80.7	19.3	
	DPDSE^b^	0.856	27.2	79.1	18.4	
PM6/L8‐BO:BTP‐eC9 (*o*‐XY)	w/o^b^	0.885	27.0	76.2	18.2	
	DPSE^b^	0.864	28.5	80.3	19.8	
PM6/L8‐BO:BTP‐eC9	w/o	0.892	26.1	77.0	17.9	[145]
	DPDS	0.862	27.9	80.3	19.3	
PM6:BTP‐eC9	w/o	0.862	26.22	71.39	16.13	[146]
	*o*ClPA	0.853	26.49	75.21	17.00	
	*m*ClPA	0.851	26.61	75.88	17.18	
	*p*ClPA	0.851	27.87	78.34	18.58	
D18:L8‐BO	w/o	0.914	25.04	80.18	18.36	
	*p*ClPA	0.903	26.02	81.01	19.04	

^a^ Certified result.

^b^ Under the same conditions, SAM is used as anode interfacial layer.

**FIGURE 11:**
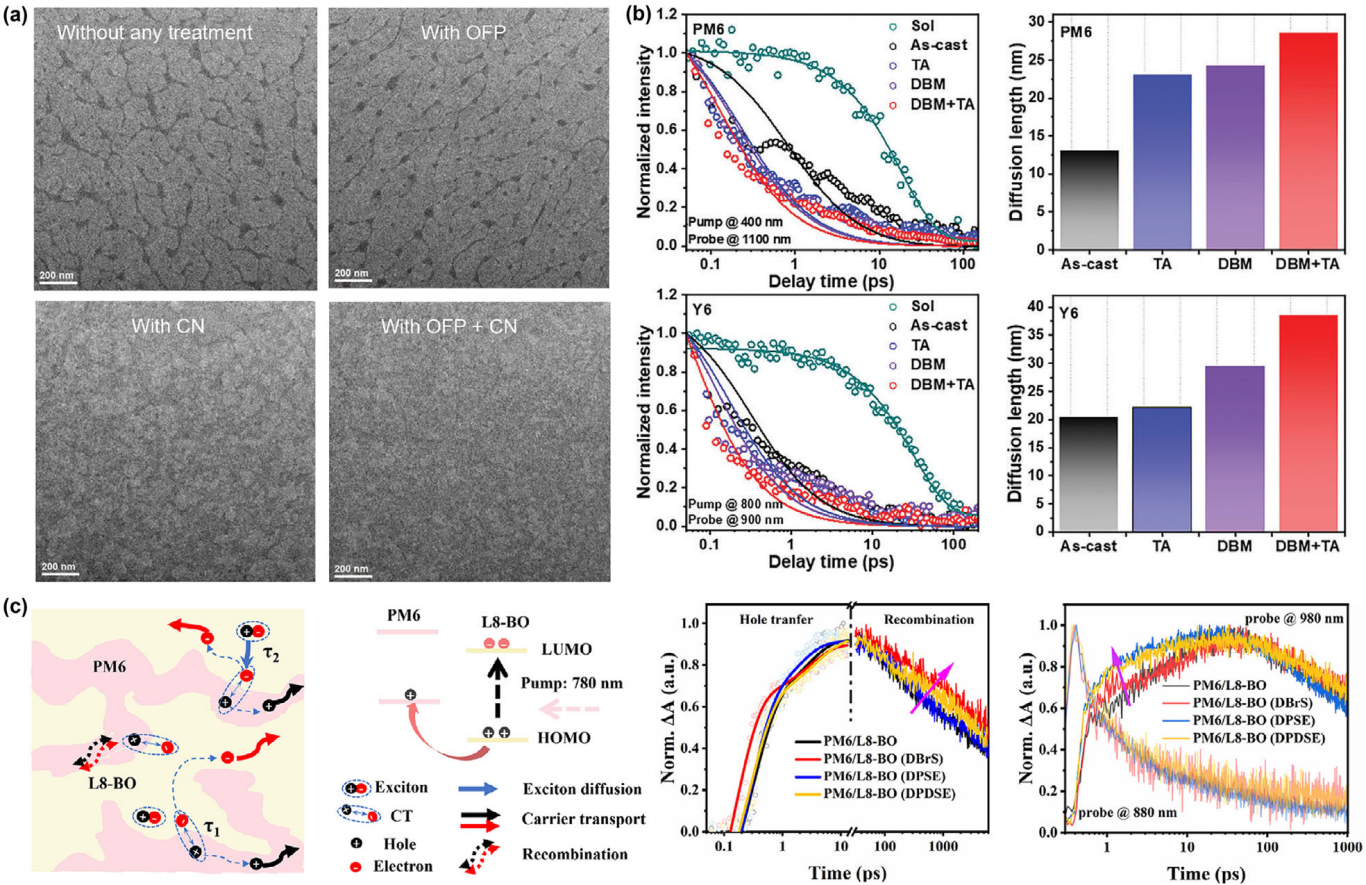
(a) Transmission electron microscopy (TEM) images of PM6:Y6 blend films treated with different additives. Reproduced with permission. Reproduced with permission [128]. Copyright 2021, Springer Nature. (b) Dynamics of singlet excitons and the exciton diffusion lengths in the PM6 and Y6 films under various processing conditions. Reproduced with permission [138]. Copyright 2024, Wiley‐VCH. (c) Schematic diagram of the charge carrier generation process in the active layer, and dynamic curves of the PM6/L8‐BO blend films with and without additives treatment. Reproduced with permission [144]. Copyright 2025, Wiley‐VCH.

In 2021, Chen et al. employed 2‐hydroxy‐4‐methoxybenzophenone (uv‐9) as a solid additive to optimize the film morphology in PM6:Y6 OSCs [134]. The results indicate that the incorporation of additive uv‐9 can lead to stronger molecular packing and more refined phase separation, resulting in improved exciton dissociation and charge transport. After uv‐9 treatment, the PCE of PM6:Y6 device has been increased from 16.00% to 17.18%, which is higher than that of 1‐CN‐treated device (16.52%). Subsequently, Yang et al. further demonstrated that the additive 2‐HM is a volatile solid additive through TGA and FTIR measurements [135]. Moreover, the in situ and ex situ characterizations reveal that additive 2‐HM regulate crystalline molecular alignment through molecular interactions between functional groups (hydroxyl, carbonyl and alkoxy) and host materials. Ultimately, a champion PCE of 18.85% is obtained in the 2‐HM‐treated PM6:L8‐BO OSCs.

In 2023, Subbiah et al. developed a novel solid additive tris(pentafluorophenyl)phosphine (TPFPP) to fine‐tune morphology of active layer in PM6:BTP‐eC9 OSCs [136]. The results suggest that additive TPFPP can induce the molecular rearrangement of active layer, leading to larger crystalline sizes and improved carrier dynamics. Consequently, the TPFPP‐treated PM6:BTP‐eC9 OSCs show a higher PCE of 16.90%, outperforming the untreated devices (15.10%). Subsequently, Yu et al. designed a series of 3D solid additives (i.e., triphenylamine (TPA), 4‐fluoro‐*N*,*N*‐diphenylaniline (FPA), tri‐p‐tolylamine (TTA) and tris(4‐fluorophenyl)amine (TFA)) to modulate morphology of active layer [137]. Among them, additive FPA exhibits excellent volatility and the strongest non‐covalent interaction with the non‐fullerene acceptor, which can induce ordered molecular stacking, resulting in enhanced charge transport and reduced energy disorder. Hence, the FPA‐treated PM6:BTP‐eC9:L8‐BO device delivers an impressive PCE of 19.2%.

In 2024, Zhang's team reported a halogen‐free solid additive dibenzoylmethane (DBM) in PM6:Y6 OSCs [138]. The findings reveal that the addition of DBM can promote ordered molecular packing of both donor and acceptor, resulting in extended exciton diffusion length and reduced charge recombination (Figure 11b). Notably, an impressive PCE of 18.7% is achieved in DBM‐treated PM6:Y6 OSCs. Additionally, DBM shows excellent universality in different non‐fullerene systems. Recently, they further reported a dual‐additive strategy to modulate the molecular aggregation of donor and acceptor in the blend film by combining solid additive DBM and solvent additive DIO [139]. Ultimately, an impressive PCE of 20.9% (certified 20.6%) is obtained in the D18:L8‐BO:BTP‐S10 system. In addition, Chen and collaborators fabricated high‐performance OSCs by combining the LbL method with solid additive DBM, which was selectively incorporated into the donor [140]. The twisted DBM molecule preferentially interacts with the A unit of donor D18, which can delay the aggregation kinetics of donor D18, resulting in highly ordered and uniform fibrilization of donor D18 in film. Eventually, the LbL‐processed OSCs exhibit an incredible PCE of 20.2%.

In 2024, Bai et al. reported a dual‐additive strategy combining a novel solid additive benzoic anhydride (BA') and solvent additive 1‐CN to modulate the aggregation behavior of non‐fullerene acceptor, resulting in favorable phase separation and more ordered molecular stacking [141]. Consequently, the as‐prepared PM6:Y6 and PM6:L8‐BO devices afford an outstanding PCE of 18.27% and 19.59%, respectively. Recently, Li et al. added the solid additive 4‐trifluoromethyl benzoic anhydride (4‐TBA) into the LbL‐based OSCs [142]. The results show that doping an appropriate amount of 4‐TBA in both the donor PM6 and acceptor L8‐BO layers yields optimal performance, with a higher PCE of 18.49% compared to the as‐cast device (17.05%).

In 2024, Wu et al. reported a novel solid additive 1,4‐diphenoxybenzene (DPB) to control the morphology of blend film [143]. The results reveal that additive DPB with a twisted configuration tends to interact with acceptor and offers adequate room for the rearrangement of active layer components after thermal annealing treatment. After DPB treatment, the PM6:Y6 blend films exhibit optimized phase separation, tighter molecular stacking and increased molecular crystallinity. Consequently, the PCE of PM6:Y6 device is remarkably improved from 15.54% to 17.45%. Moreover, the DPB‐processed D18‐Cl:N3 OSCs afford an impressive PCE of 19.04%. Moreover, Duan et al. employed 1,3‐diphenoxybenzene (DB) to modulate the donor/acceptor molecular aggregation and packing in the semi‐transparent OSCs, leading to broadened spectral response into the near‐infrared region [147]. In 2025, Liu et al. developed three selenium‐based additives (solvent additives: 2,5‐dibromoselenophene (DBrS) and diphenyl selenide (DPSE); and solid additive: diphenyl diselenide (DPDSE)) to induce fibrillation of acceptor in the OSCs fabricated via sequential deposition method, resulting in a favorable match between the blend morphology (molecular crystallinity and vertical phase distribution) and the optoelectronic properties (longer exciton diffusion length as well as improved exciton dissociation and charge transport) (Figure 11c) [144]. As a result, the DBrS‐, DPSE‐ and DPDSE‐treated PM6/L8‐BO OSCs exhibit higher PCEs of 18.6%, 18.4% and 18.1%, respectively, compared to the as‐cast device (17.3%). Moreover, the DPSE‐treated device processed with non‐halogenated *o*‐XY shows an impressive PCE of 19.8%. Recently, Zheng et al. developed a non‐halogenated solid additive, diphenyl disulfide (DPDS), to study its effect on film‐forming kinetics of the active layer [145]. Benefitting from the optimized morphology, the DPDS‐processed PM6/L8‐BO:BTP‐eC9 ternary OSCs afford a high PCE of 19.3%, significantly outperforming the as‐cast device (17.9%).

In 2025, Xia et al. reported three imide‐based additives (i.e., *N*‐(*o*‐chlorophenyl)phthalimide (*o*ClPA), *N*‐(*m*‐chlorophenyl)phthalimide (*m*ClPA) and *N*‐(*p*‐chlorophenyl)phthalimide (*p*ClPA)) with large dipole moments to improve the photovoltaic performance of OSCs [146]. The results demonstrate that additives CIPA can induce the non‐fullerene acceptor to first nucleate and then grow, leading to high‐quality acceptor domains with improved crystallinity. As a result, the *o*ClPA‐, *m*ClPA‐ and *p*ClPA‐treated PM6:BTP‐eC9 devices exhibit improved PCEs of 17.00%, 17.18% and 18.58%, respectively, compared to the as‐cast device (16.13%). Moreover, an impressive PCE of 19.04% is obtained in the *p*ClPA‐processed D18:L8‐BO OSCs.

As discussed above, volatile PAHs and their derivatives can also be used as additives to optimize device performance, which can be divided into fused‐ring structures (such as naphthalene, anthracene, and fluorene) and non‐fused‐ring structures (such as biphenyl, triphenylamine, and benzoic anhydride). In the reported work, the effects of the alkyl chain length of the additives, as well as the position, type, and number of halogen substituents on the additives, on the device performance have been studied. In addition, the additive‐induced morphology optimization, charge carrier dynamics and the corresponding device performance have been systematically investigated, thereby deepening the understanding of the mechanism of volatile additives.

#### 2.1.4 Heterocyclic Aromatic Hydrocarbons (HAHs) Derivatives

As shown in Figure 12 and Table 5, HAHs derivatives (such as thiophene, pyridine, pyrazine, triazine, pyrimidine, and thiazole) exhibit extraordinary capabilities in morphology regulation and device performance enhancement. In 2022, Guo et al. employed thiophene derivatives, 2,5‐dibromothiophene (HBrT) and 2,5‐dibromo‐3,4‐difluoro‐thiophene (FBrT), as solvent additives in OSCs [148]. The results suggest that the solvent additive FBrT can readily dissolve both donor and acceptor owing to the “like dissolve like” principle (Figure 13a), thereby prolonging the crystallization and phase separation kinetics of the blend film. The FBrT‐treated blend film exhibits favorable phase separation, increased molecular crystallinity and P‐i‐N‐like structure, yielding a high PCE of 17.9% in the PM6:Y6 system. Subsequently, Zhang et al. reported a series of volatile additives based on thiophene units with different halogen substitutions (solid additive: 2,5‐diiodothiophene (DIT); solvent additives: 2,5‐dibromothiophene (DBT or HBrT) and 2,5‐dichlorothiophene (DCT)) in PM6:Y6 OSCs [149]. As a result, the DCT‐, DBT‐ and DIT‐treated devices exhibit higher PCEs of 16.80%, 17.29% and 17.48%, respectively, compared to the control devices (15.99%) and DIB‐treated OSCs (16.63%). In 2023, Cui et al. employed thiophene derivatives containing different numbers of halogen atoms (namely, 2,5‐dichlorothiophene (Th2Cl or DCT), 2,3,5‐trichlorothiophene (Th3Cl) and tetrachlorothiophene (Th4Cl)) as additives to regulate the aggregation behavior of conjugated molecules in the D18:BTP‐eC9‐4F system [150]. The intermolecular interaction between acceptor BTP‐eC9‐4F and additive Th4Cl enables Th4Cl to modulate the growth dynamics of BTP‐eC9‐4F, leading to improved crystallization quality. Ultimately, the Th4Cl‐treated D18:BTP‐eC9‐4F OSCs exhibit a high PCE of 19.2%. In 2024, Wang et al. used a series of thiophene derivatives (i.e., solvent additives: DBT and 2‐bromo‐5‐iodothiophene (BIT); solid additive: DIT) as additives in non‐fullerene OSCs [151]. The results indicate that the asymmetric effect of BIT allows it to mix well with acceptor Y6 and form dimers, leading to enhanced molecular crystallinity. As a result, the devices treated with BIT exhibit a higher efficiency of 17.43% compared to those treated with DBT (16.36%) and DIT (16.69%). Moreover, the BIT‐treated PM6:L8‐BO OSCs deliver an impressive PCE of 18.53%. In 2025, Liao et al. systematically investigated the influence of nine halogen‐functionalized additives, encompassing three categories of methyl halides, thiophene halides and benzene halides, on the performance of LbL‐based devices [152]. Among them, additive BIT shows the most favorable performance, yielding an excellent PCE of 20.12% in the PM6/L8‐BO OSCs. Moreover, Chen and co‐workers developed several perhalogenated thiophenes‐based solid additives (i.e., 3,4‐dibromo‐2,5‐diiodothiophene (SA‐T1), 2,5‐dibromo‐3,4‐diiodothiophene (SA‐T2), 2,3‐dibromo‐4,5‐diiodothiophene (SA‐T3) and 2,5‐dichloro‐3,4‐diiodothiophene (SA‐T5)) in all‐PSCs, which exhibited extraordinary performance enhancement and good universality [30, 153].

**FIGURE 12:**
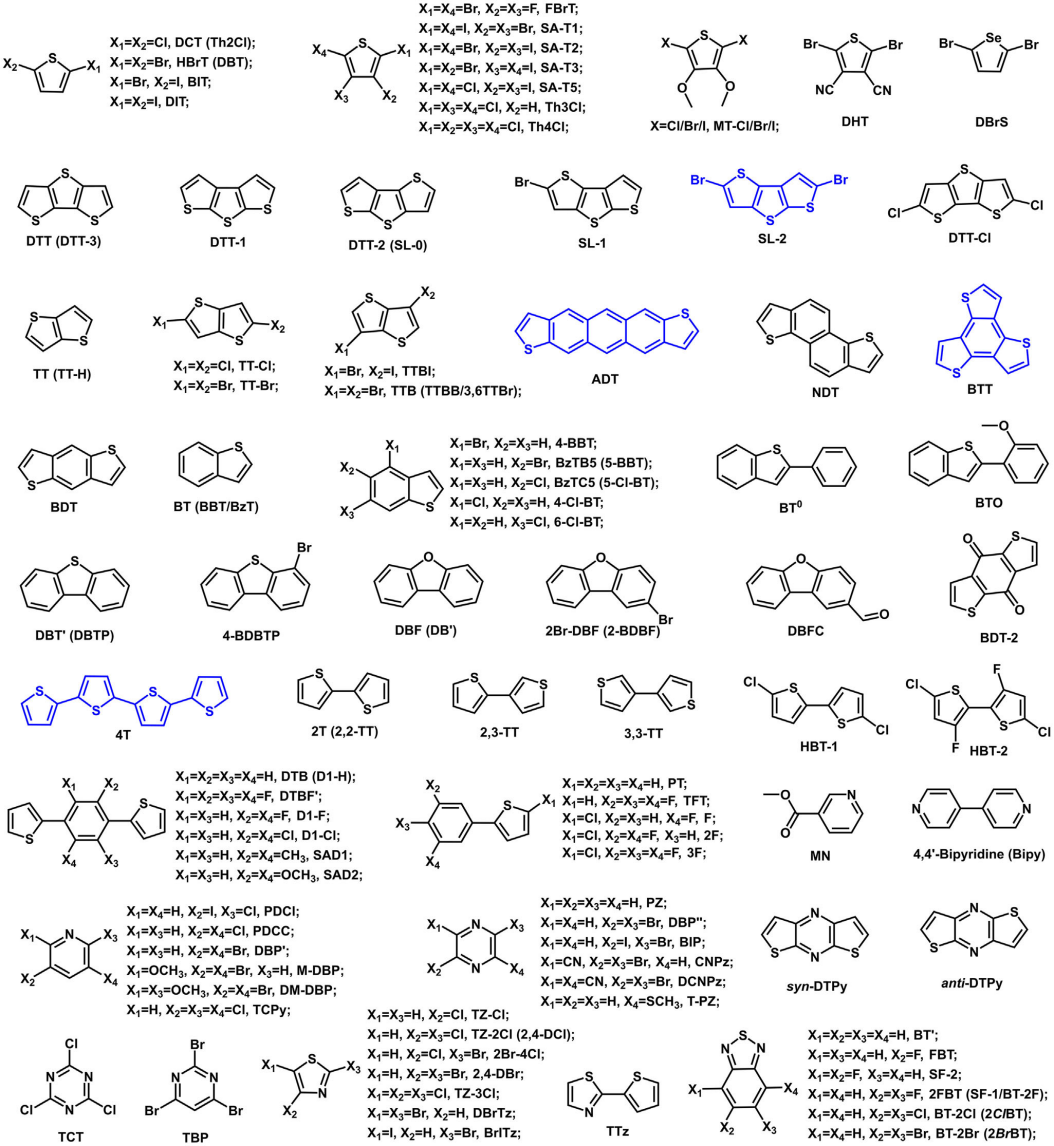
Chemical structures of volatile additives based on HAHs Derivatives (Blue: non‐volatile additives / additives that do not fully volatilize at the appropriate temperature).

**TABLE 5:** Summary of photovoltaic parameters for devices processed with volatile additives based on HAHs derivatives.

Active layer	Additive	*V*_oc_ [V]	*J*_sc_ [mA cm^−2^]	FF [%]	PCE [%]	Refs.
PM6:Y6	1‐CN	0.85	25.93	74.8	16.5	[148]
	HBrT	0.85	26.37	75.9	17.0	
	FBrT	0.85	26.78	78.6	17.9	
PM6:Y6	w/o	0.864	26.64	70.9	15.99	[149]
	DIB	0.842	27.46	73.5	16.63	
	DCT	0.851	27.12	74.5	16.80	
	DBT	0.848	27.32	75.0	17.29	
	DIT	0.852	27.59	75.4	17.48	
D18:BTP‐eC9‐4F	w/o	0.867	27.6	73.6	17.6	[150]
	Th2Cl	0.853	27.3	74.3	17.3	
	Th3Cl	0.856	27.3	77.5	18.2	
	Th4Cl	0.875	27.5	79.7	19.2	
PM6:Y6	w/o	0.856	24.60	71.95	15.15	[151]
	BIT	0.846	26.43	77.90	17.43	
	DBT	0.851	25.79	74.56	16.36	
	DIT	0.844	25.96	76.18	16.69	
PM6/L8‐BO	w/o	0.925	25.75	75.10	17.89	[152]
	BIT	0.910	27.12	80.97	20.12	
PM6:Y6	w/o	0.853	27.02	70.47	16.24	[154]
	MT‐I	0.825	28.60	75.95	17.93	
PM6:Y6	w/o	0.844	24.95	74.40	15.67	[155]
	1‐CN	0.854	25.70	74.80	16.42	
	DHT	0.849	26.12	76.58	16.83	
	1‐CN&DHT	0.859	26.64	78.38	17.93	
PBDB‐TF:BO4Cl	w/o	0.855	24.5	70.6	14.8	[156]
	DTB	0.852	25.0	73.5	15.6	
	DTBF'	0.846	26.2	77.0	17.1	
PM6:L8‐BO	w/o	0.883	24.36	77.39	16.65	[157]
	SAD1	0.880	26.40	78.12	18.15	
	SAD2	0.889	26.73	79.32	18.85	
	SAD2	0.881	27.07	78.3	18.7^a^	
PM6:L8‐BO	w/o	0.907	25.79	74.3	17.37	[158]
	D1‐H	0.867	26.71	76.1	17.62	
	D1‐F	0.873	26.76	75.9	17.73	
	D1‐Cl	0.886	26.62	78.8	18.59	
D18:L8‐BO	w/o	0.925	25.46	75.08	17.70	[159]
	PT	0.919	26.00	76.25	18.28	
	TFT	0.912	26.20	79.69	19.19	
PM6:Y6	w/o	0.846	26.7	71.2	16.1	[160]
	2T	0.854	26.9	78.6	18.1	
	4T	0.834	26.8	76.8	17.2	
D18:L8‐BO:PY‐C11	w/o	0.934	26.57	71.83	17.83	[161]
	HBT‐1	0.916	26.48	77.42	18.76	
	HBT‐2	0.910	27.41	80.23	20.01	
PM6:L8‐BO	w/o	0.937	25.09	71.83	16.89	[162]
	2,2‐TT	0.895	26.49	79.32	18.80	
	2,3‐TT	0.913	26.54	79.33	19.22	
	3,3‐TT	0.916	26.52	79.61	19.34	
D18:L8‐BO	3,3‐TT	0.913	26.98	82.47	20.32	
PM6:Y6	w/o	0.843	26.45	73.16	16.30	[29]
	BDT	0.841	27.61	77.11	17.91	
PTVT‐T:BTP‐eC9	w/o	0.850	22.17	71.13	13.41	[163]
	BT	0.823	27.35	78.83	17.75	
	BDT	0.828	20.16	60.19	10.05	
	BTT	0.874	18.88	62.98	10.39	
D18‐Cl:N3	w/o	0.872	26.31	74.9	17.18	[164]
	BBT	0.912	24.43	79.6	17.74	
	BBT/SVA	0.870	26.71	80.1	18.60	
	BBT/SVA^b^	0.875	27.63	80.8	19.53	
PMZ‐10:Y6	1‐CN	0.869	27.40	77.2	18.38	[165]
	1‐CN&BT	0.870	27.48	79.6	19.03	
D18:Y6	w/o	0.864	25.8	69.0	15.4	[166]
	BzT	0.851	26.4	78.9	17.7	
PM6:L8‐BO	w/o	0.913	25.22	71.89	16.55	[167]
	4‐Cl‐BT	0.894	26.73	80.41	19.21	
	5‐Cl‐BT	0.890	26.92	80.72	19.34	
	6‐Cl‐BT	0.884	26.65	80.28	18.92	
D18:L8‐BO:BTP‐eC9‐4F	5‐Cl‐BT^b^	0.891	28.41	82.56	20.89	
PM6:L8‐BO	w/o	0.922	24.55	72.97	16.51	[168]
	4‐BBT	0.910	25.14	77.21	17.67	
	5‐BBT	0.902	25.85	77.04	17.96	
	4‐BBT&5‐BBT	0.891	27.32	79.39	19.32	
D18/L8‐BO	w/o	0.919	25.80	76.5	18.15	[169]
	D(BTO)/A	0.911	26.13	78.1	18.60	
	D/A(BTO)	0.912	26.20	80.2	19.16	
PM6:L8‐BO	w/o	0.893	24.26	75.7	16.41	[170]
	NDT	0.912	26.46	78.1	18.85	
PM6:Y6	w/o	0.858	25.24	71.85	15.56	[23]
	DPS	0.825	26.61	76.81	16.86	
	DBT'	0.853	26.68	78.44	17.85	
PM6:L8‐BO	w/o	0.902	25.61	71.70	16.60	[127]
	FL	0.885	26.64	76.80	18.10	
	DBT'	0.877	26.52	75.84	17.63	
	DBF	0.893	27.01	77.36	18.62	
PM6:L8‐BO:BTP‐eC9	DBF	0.881	27.42	79.08	19.11	
PM6:PY‐DT	w/o	0.972	24.56	65.9	15.73	[171]
	DBTP	0.952	25.21	76.5	18.36	
	4‐BDBTP	0.951	25.85	78.5	19.30	
	4‐BDBTP	0.946	25.47	78.1	18.82^a^	
PM6/L8‐BO	w/o	0.919	26.88	71.58	17.67	[172]
	DB'	0.904	27.38	74.02	18.32	
	1‐CN	0.900	27.31	76.31	18.74	
	1‐CN&DB'	0.898	27.56	79.15	19.58	
PM6/L8‐BO	w/o	0.897	27.64	75.43	18.71	[173]
	2Br‐DBF	0.899	27.99	79.48	20.02	
PTQ10:*m*‐BTP‐PhC6:PC_71_BM	1‐CN	0.860	25.66	78.6	17.35	[174]
	1‐CN&DTT	0.869	26.99	80.6	18.89	
PM6:BTP‐eC9	w/o	0.853	26.25	75.71	16.96	[175]
	DTT	0.858	27.56	78.14	18.50	
PM6:BTP‐eC9:L8‐BO	w/o	0.876	27.57	77.12	18.62	
	DTT	0.883	28.37	79.86	20.01	
PM6:L15	w/o	0.96	23.23	64.74	14.39	[176]
	DTT‐1	0.95	23.98	73.71	16.72	
	DTT‐2	0.95	24.86	76.25	18.03	
	DTT‐3	0.94	21.53	73.60	14.82	
	DTT‐2 (SVA/CS_2_)	0.93	25.95	77.26	18.72	
PM6:PY‐IT	w/o	0.935	24.78	63.9	14.82	[177]
	SL‐0	0.939	25.57	76.8	18.37	
	SL‐1	0.935	26.10	78.6	19.19	
	SL‐2	0.929	25.09	71.5	16.60	
	SL‐1	0.935	26.04	78.3	19.08^a^	
PM6:Y6	w/o	0.861	26.14	71.30	16.05	[178]
	TT‐Cl	0.861	26.86	78.69	18.20	
PM6:D18:Y6	w/o	0.846	26.84	74.05	16.81	
	TT‐Cl	0.865	27.73	79.01	18.95	
PM6:Y6	w/o	0.854	26.7	67.7	15.4	[179]
	TT‐Cl	0.866	26.5	76.1	17.5	
D18:BTP‐eC9‐4F	w/o	0.866	27.5	73.1	17.4	
	TT‐Cl	0.868	27.7	79.8	19.3	
PM6:Y6	w/o	0.849	26.60	70.55	15.94	[180]
	1‐CN	0.846	26.91	74.51	17.17	
	TT	0.847	27.91	75.10	17.75	
	TTB	0.838	27.88	73.58	17.33	
PM6:L8‐BO	w/o	0.895	25.0	75.6	16.9	[181]
	TT	0.886	26.3	78.3	18.2	
	TTBB	0.893	26.8	79.7	19.1	
	TTBI	0.889	26.7	78.7	18.7	
D18/L8‐BO	D(TTBB)/A(TTBI)	0.919	27.2	79.8	19.9	
PM6:L8‐BO	w/o	0.91	25.2	75.7	17.5	[182]
	3,6TTBr	0.89	26.8	80.4	19.1	
PM6:D18:L8‐BO	3,6TTBr	0.90	27.4	81.2	20.1	
J51:N2200	w/o	0.81	15.05	60.35	7.37	[183]
	Bipy	0.85	14.74	69.44	8.73	
PM6/BTP‐eC9	w/o	0.830	26.56	76.79	16.93	[184]
	MN	0.850	27.64	78.44	18.42	
	MN (28.82 cm^2^)	13.15	1.659	73.50	16.04	
PM6:Y6	w/o	0.8394	27.38	70.16	16.12	[185]
	PDCl	0.8369	27.51	78.69	18.12	
PM6:BTP‐eC9	w/o	0.8563	27.67	72.38	17.15	
	PDCl	0.8528	28.27	78.91	19.02	
PM6:L8‐BO	w/o	0.9128	27.26	73.25	18.23	[186]
	PDCC	0.9068	27.48	82.16	20.47	
D18:L8‐BO	w/o	0.915	25.86	77.8	18.41	[187]
	DBP	0.913	25.31	79.2	18.31	
	M‐DBP	0.911	26.38	79.8	19.18	
	DM‐DBP	0.883	25.05	80.8	17.87	
D18:L8‐BO	w/o	0.927	25.81	77.29	18.49	[188]
	TCPy	0.923	27.19	80.21	20.13	
PTQ10:*m*‐BTP‐PhC6	w/o	0.883	26.55	79.9	18.73	[189]
	CNPz	0.884	27.20	81.8	19.67	
	DCNPz	0.884	26.19	79.2	18.34	
PM1:BTP‐eC9	w/o	0.850	28.73	75.27	18.38	[190]
	BIP	0.851	28.64	78.53	19.14	
PM1/BTP‐eC9	BIP	0.851	28.76	80.17	19.63	
PM1/BTP‐eC9:eC9‐2Cl	BIP	0.873	28.70	80.24	20.11	
PM6:Y6	w/o	0.837	27.23	73.89	16.85	[191]
	PZ	0.845	27.22	76.82	17.67	
	T‐PZ	0.863	27.90	80.85	19.47	
PM6:BTP‐TO2:AT‐β2O	w/o	0.867	27.47	80.49	19.16	
	T‐PZ	0.894	27.75	81.20	20.14	
D18:L8‐BO	w/o	0.924	26.06	75.51	18.13	[192]
	TCT	0.925	26.50	79.55	19.50	
PM6:BTP‐eC9	w/o	0.864	25.18	75.6	16.43	[193]
	TCT	0.854	27.13	78.5	18.18	
D18:L8‐BO	w/o	0.912	25.98	76.36	18.25	[194]
	TBP	0.908	27.91	82.28	20.87	
	TBP	0.893	28.00	81.47	20.37^a^	
D18:L8‐BO	w/o	0.92	26.4	78.3	18.9	[195]
	*syn*‐DTPy	0.91	26.6	80.8	19.6	
	*anti*‐DTPy	0.92	27.2	81.9	20.5	
PM6:L8‐BO	w/o	0.896	25.5	76.2	17.4	[196]
	DBrTz	0.886	26.8	80.0	19.0	
	BrITz	0.886	26.3	78.3	18.2	
PM6:L8‐BO	w/o	0.893	26.10	75.86	17.7	[197]
	TZ‐Cl	0.899	26.71	76.31	18.3	
	TZ‐2Cl	0.901	27.01	75.93	18.5	
	TZ‐3Cl	0.903	27.29	80.19	19.8	
PM6:BTP‐eC9‐4F:DM‐F	w/o	0.897	27.32	77.50	19.0	
	TZ‐3Cl	0.902	28.01	79.83	20.2	
PM6/Y6	w/o	0.852	25.45	68.02	14.75	[198]
	D(BT')/A	0.849	25.70	71.02	15.49	
	D/A(BT')	0.835	25.13	70.78	14.85	
	D(FBT)/A	0.842	26.14	75.57	16.63	
	D/A(FBT)	0.831	25.48	72.38	15.40	
	D(2FBT)/A	0.839	25.83	72.88	15.79	
	D/A(2FBT)	0.830	25.31	70.98	14.91	
PM6:PY‐DT	w/o	0.972	24.81	62.7	15.17	[199]
	SF‐1	0.964	25.36	72.5	17.83	
	SF‐2	0.970	25.99	74.5	18.83	
PM6:L8‐BO	w/o	0.907	26.45	76.28	18.31	[200]
	BT‐2F	0.895	26.84	77.86	18.72	
	BT‐2Cl	0.876	26.73	75.73	17.74	
	BT‐2Br	0.884	26.97	80.15	19.11	
D18/L8‐BO	w/o	0.900	25.97	78.96	18.45	[201]
	2*F*BT	0.888	26.47	79.90	18.78	
	2*Cl*BT	0.889	26.49	79.12	18.63	
	2*Br*BT	0.884	26.86	80.66	19.15	
D18/L8‐BO:eC9‐4ClO	2*Br*BT	0.885	27.70	82.07	20.12	

^a^ Certified result.

^b^ Under the same conditions, SAM is used as anode interfacial layer.

**FIGURE 13:**
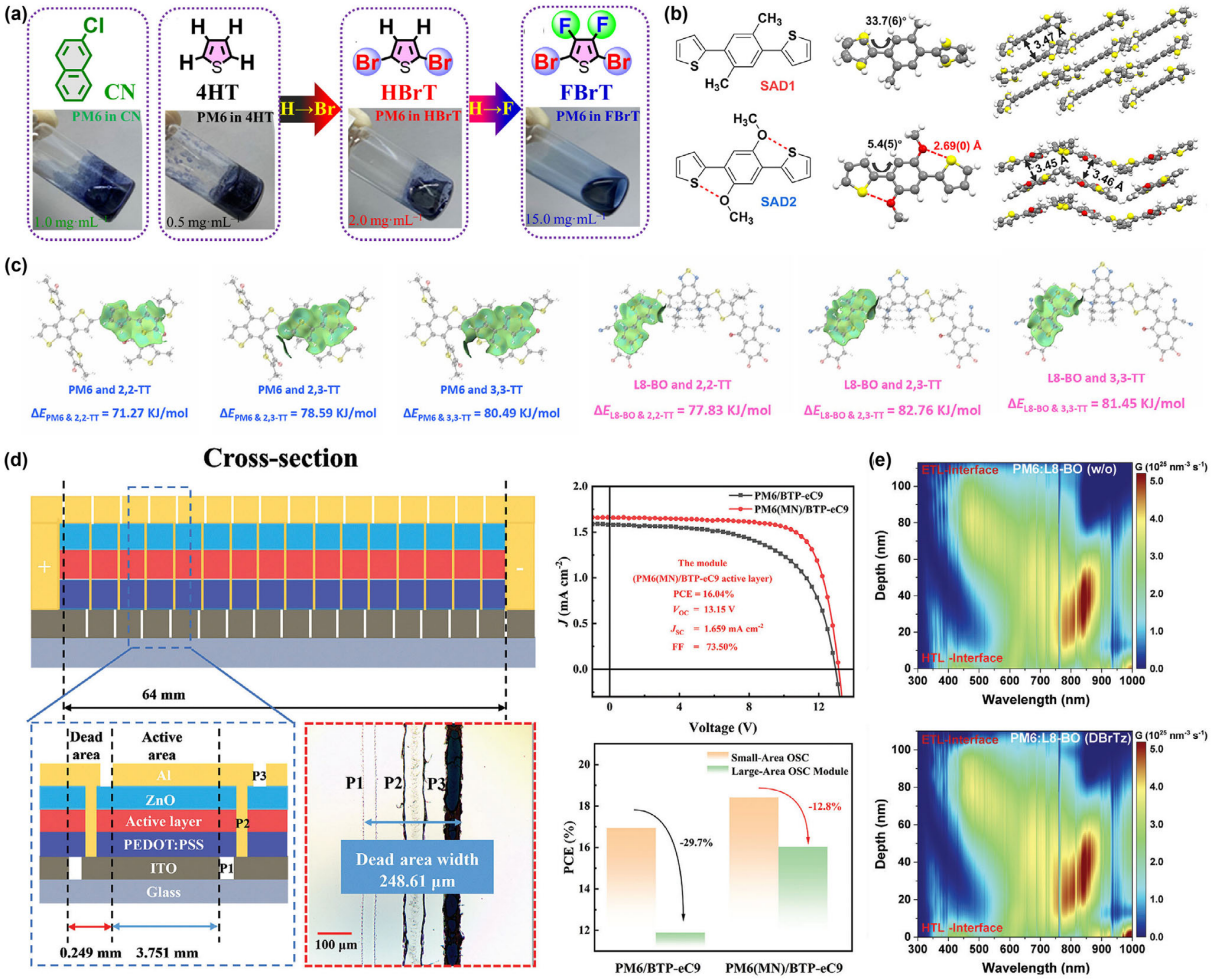
(a) Solubility test of donor PM6 in different solvent additives. Reproduced with permission [148]. Copyright 2022, Royal Society of Chemistry. (b) Molecular structure, single‐crystal structures, and molecular packing of additives SAD1 and SAD2. Reproduced with permission [157]. Copyright 2022, American Chemical Society. (c) Binding energies between PM6 or L8‐BO and different additives. Reproduced with permission [162]. Copyright 2025, Wiley‐VCH. (d) Schematic diagram of the large‐area OSCs module and its corresponding *J*‐*V* curves and PCE loss from small‐area devices to large‐area modules. Reproduced with permission [184]. Copyright 2024, Wiley‐VCH. (e) Calculated exciton generation contour of the PM6:L8‐BO blend film processed with and without additive DBrTz. Reproduced with permission [196]. Copyright 2024, Wiley‐VCH.

In addition, methoxy and cyano groups can also be introduced into thiophenes‐based volatile solid and solvent additives. In 2025, Jiang et al. designed and synthesized halogenated methoxylated thiophenes‐based additives (i.e., solid additve: 2,5‐diiodo‐3,4‐dimethoxythiophene (MT‐I); solvent additive: 2,5‐dibromo‐3,4‐dimethoxythiophene (MT‐Br) and 2,5‐dichloro‐3,4‐dimethoxythiophene (MT‐Cl)) in PM6:Y6 OSCs [154]. Compared with the as‐cast device (16.24%), the PM6:Y6 OSCs processed with additives MT‐I, MT‐Br and MT‐Cl afford remarkably enhanced PCEs of 17.93%, 17.38% and 17.06%, respectively. In 2025, Liu et al. introduced a solid‐solvent hybrid additives strategy by utilizing solvent additive 1‐CN and solid additive 2,5‐dibromo‐3,4‐thiophenedinitrile (DHT) in PM6:Y6 OSCs, which could induce more ordered molecular packing and improved charge transport pathways [155]. Eventually, the dual‐additive‐treated PM6:Y6 devices deliver a high PCE of 17.93%, surpassing those of 1‐CN‐treated OSCs (16.42%) and DHT‐treated OSCs (16.83%).

In 2021, Yu et al. applied 1,4‐di(thiophen‐2‐yl)benzene (DTB) and its analog 2,2'‐(perfluoro‐1,4‐phenylene)dithiophene (DTBF') as additive to control the morphology in non‐fullerene OSCs [156]. Benefitting from the stronger charge‐quadrupole interaction, the introduction of DTBF' can induce more ordered and compact molecular packing in the active layer, resulting in broadened light absorption and improved charge transport. Eventually, the DTBF'‐treated PBDB‐TF:BO4Cl OSCs show an excellent PCE of 17.1%, which is higher than those of the as‐cast device (14.8%) and DTB‐processed device (15.6%). In 2022, Li et al. designed and synthesized two solid additives, namely the twisted SAD1 and the planar SAD2 with S···O noncovalent intramolecular interactions (Figure 13b), to study the effect of the conformation of volatile solid additives on the photovoltaic performance of devices [157]. Compared with SAD1, additive SAD2 with smaller spatial occupation can be more readily inserted into acceptor Y6 molecules, leading to tighter π–π stacking of Y6. Ultimately, the SAD2‐processed PM6:L8‐BO OSCs deliver a record PCE of 18.85% (certified 18.7%). In 2024, Su et al. developed two novel DTB (D1‐H)‐based additives with different halogen substituents (i.e., fluorinated D1‐F and chlorinated D1‐Cl) [158]. Compared with D1‐H, both D1‐F and D1‐Cl exhibit stronger non‐covalent interactions with the host materials. Consequently, D1‐Cl‐treated blend film displays an enhanced molecular stacking and optimized phase separation, yielding an outstanding PCE of 18.59% in PM6:L8‐BO devices. In 2024, Liu et al. designed and synthesized two highly molecules 2‐phenylthiophene (PT) and 2‐(3,4,5‐trifluorophenyl)thiopheneb (TFT) as solid additives to regulate the aggregation behavior of D18:L8‐BO blend film [159]. The results reveal that additive TFT shows stronger interactions with the non‐fullerene acceptor than PT owing to the high electronegativity of fluorine atom, leading to more ordered molecular packing. As a result, the TFT‐processed D18:L8‐BO OSCs exhibit a higher PCE of 19.19% compared to the as‐cast device (17.70%) and PT‐treated device (18.28%). Recently, Song et al. designed a series of fluorinated additives with different numbers of fluorine atoms (F, 2F, and 3F) to precisely tune the strength of their interactions with the host materials, thereby affecting the crystallization kinetics, phase separation, and molecular packing of the active layer [202]. Among them, the moderately fluorinated 2F provides optimal regulation, which can suitably extend crystallization time, facilitate acceptor L8‑BO fibril formation, and generate a refined dual‐fibril network in the blend film. Eventually, the 2F‐treated D18:L8‐BO device delivers a higher PCE of 20.01% than the as‐cast device (17.99%) and the device treated with F (19.22%) and 3F (19.42%).

In 2024, Liang et al. introduced two oligothiophene additives (namely 2T and 4T) in PM6:Y6 OSCs to investigate their effects on the film formation process and nanoscale morphology of the active layer [160]. Upon thermal annealing treatment, 2T is entirely eliminated, whereas 4T is retained in the blend film. Moreover, additive 2T can effectively regulate the aggregation behavior of both donor and acceptor during the solution‐to‐film transition process, leading to more ordered molecular stacking and more favorable vertical phase separation. As a result, the 2T‐treated PM6:Y6 OSCs afford a champion PCE of 18.1%, outperforming both the control device (16.1%) and 4T‐processed device (17.2%). Subsequently, Wang et al. designed and synthesized two oligothiophene additives (i.e., 5,5'‐dichloro‐2,2'‐bithiophene (HBT‐1) and 3,3'‐difluoro‐5,5'‐dichloro‐2,2'‐bithiophene (HBT‐2)) to further explore the influence of additive conformation on the photovoltaic performance of device [161]. Compared with HBT‐1, additive HBT‐2 with dual S···F noncovalent conformational locks displays enhanced molecular planarity and crystallinity, thereby inducing more ordered molecular packing in blend film. Ultimately, the HBT‐2‐treated D18:L8‐BO:PY‐C11 OSCs show a much higher PCE of 20.01% compared to the HBT‐1‐processed device (18.76%). In 2025, Shi et al. designed a series of bithiophene isomers (i.e., 2,2‐TT (2T), 2,3‐TT and 3,3‐TT) as additives, which exhibit distinct ESP distributions, leading to varying binding energy values with the host materials (Figure 13c) [162]. Systematic analysis indicates that the addition of 2,2‐TT can significantly enhance the π‐π stacking of acceptor L8‐BO but has little influence on the aggregation of donor PM6. In contrast, 3,3‐TT induces not only highly ordered molecular stacking in L8‐BO but also denser π‐π stacking in PM6. Ultimately, the PM6:L8‐BO OSCs processed with 2,2‐TT, 2,3‐TT and 3,3‐TT afford higher PCEs of 18.80%, 19.22% and 19.34%, respectively, compared to the as‐cast device (16.89%). Moreover, the 3,3‐TT‐treated D18:L8‐BO OSCs exhibit an excellent PCE of 20.32%.

In 2024, Dong et al. proposed BDT as solid additive in Y‐series OSCs, which is a commonly used building block of donors [29]. The findings demonstrate that the intermolecular interaction between additive BDT and acceptor Y6 (particularly for the dithienothiophen[3,2‐*b*]‐pyrrolobenzothiadiazole (BTP) unit) can fine‐tune the arrangement of acceptor, leading to ideal phase separation and improved carrier dynamics. Finally, the efficiency of PM6:Y6 OSCs has been increased from 16.30% to 17.91% after treatment with additive BDT. Moreover, the universal applicability of additive BDT has been further validated in other Y‐series systems. Subsequently, Liu et al. reported a novel in situ volatile additive benzothiophene (BT), which could be completely removed during the spin‐coating process [163]. Comparative studies with the anneal‐volatile BDT and nonvolatile benzotrithiophene (BTT), further demonstrate the advantages of BT additive in refining molecular interactions with the host materials and optimizing the morphology in PTVT‐T:BTP‐eC9 blend films. Consequently, the BT‐treated PTVT‐T:BTP‐eC9 OSCs show a champion PCE of 17.75%, significantly outperforming both BDT‐ and BTT‐processed counterparts. Interestingly, we proposed a novel combined strategy to sequentially regulate the active layer morphology by integrating additive BBT (BT) and SVA treatment [164]. Notably, the as‐prepared D18‐Cl:N3 binary OSCs afford an impressive PCE of 19.53%. Subsequently, Shi et al. employed a dual‐additive strategy (BT and 1‐CN) to optimize the morphology of the active layer [165]. Impressively, the corresponding PMZ‐10:Y6 devices offer an excellent PCE of 19.03%. In 2025, Shan et al. further proved that the volatile solid additives BzT (BT/BBT) and its analogs (i.e., 5‐bromobenzo[*b*]thiophene (BzTB5) and 5‐chlorobenzo[*b*]thiophene (BzTC5)) display great potential in morphology regulation and performance enhancement [166]. Ultimately, a high PCE of 17.7% is achieved in BzT‐treated D18:Y6 OSCs. In 2026, Shi et al. used chlorine‐substituted BT, namely 4‐Cl‐BT, 5‐Cl‐BT and 6‐Cl‐BT, to modulate crystallization kinetics of the active layer [167]. Due to the favorable spatial ESP distribution, additive 5‐Cl‐BT shows more synergistic dipole‐dipole interactions and dispersion forces, which in turn enhance its intermolecular interactions with both donor PM6 and acceptor L8‐BO. As a result, the PM6:L8‐BO and D18:L8‐BO:BTP‐eC9‐4F OSCs treated with 5‐Cl‐BT deliver high PCEs of 19.34% and 20.89%, respectively. Additionally, Zhao et al. found that the mixed additives (4‐bromobenzothiadiazole (4‐BBT) and 5‐bromobenzothiadiazole (5‐BBT)) could effectively slow down film formation, thereby extending crystallization time and enhancing π‐π stacking, while reducing recombination losses [168]. Consequently, the PM6:L8‐BO OSCs treated with the mixed additives (4‐BBT and 5‐BBT) exhibit a higher PCE of 19.32% compared to those treated with 4‐BBT (17.67%) or 5‐BBT (17.96%). Moreover, Xiao et al. synthesized a novel solid additive 2‐(2‐methoxyphenyl)benzo[*b*]thiophene (BTO), which was separately introduced into the donor and acceptor layers to control the morphology of the active layer, thereby improving the PCE of devices [169]. Finally, an excellent PCE of 19.16% is obtained in the D18/L8‐BO‐based binary OSCs. In 2023, Zhang et al. developed two solid additives (i.e., anthra[2,3‐*b*:6,7‐*b*']dithiophene (ADT) and naphtho[1,2‐*b*:5,6‐*b*']dithiophene (NDT)) to explore their influence on morphology of the photoactive layer [170]. Unlike non‐volatile ADT, volatile additive NDT can induce more compact and ordered molecular stacking in blend film, thus yielding an improved PCE of 18.85% in PM6:L8‐BO OSCs.

In 2023, Xiao et al. developed a solidification of solvent additive (SSA) strategy by introducing a covalent bond in solvent diphenyl sulfide (DPS) to form solid additive dibenzothiophene (DBT') [23]. The results suggest that the DBT'‐treated blend films exhibit ordered molecular stacking and appropriate phase separation due to the high planarity and strong volatility of DBT'. Accordingly, the DBT'‐treated PM6:Y6 OSCs afford a high PCE of 17.85%, outperforming the as‐cast device (15.56%) and DPS‐processed device (16.86%). Moreover, the general applicability of the SSA approach was also assessed by using different additive systems, including DPE vs. dibenzofuran (DBF), diphenyl methane (DPM) vs. dibenzopentacyclic (DBP or FN), and 4‐phenoxy benzaldehyde (POB) vs. dibenzofuran‐2‐carboxaldehyde (DBFC). Interestingly, Xu et al. compared three solid additives (i.e., FL (FN/DBP), DBT' and DBF) with similar structures in OSCs [127]. As a result, compared with the as‐cast device, the FL‐, DBT'‐ and DBF‐treated PM6:L8‐BO devices show improved PCEs of 18.10%, 17.63% and 18.62%, respectively. Moreover, an impressive PCE of 19.11% is achieved in DBF‐processed PM6:L8‐BO:BTP‐eC9 OSCs. In 2024, Ma et al. used dibenzothiophene (DBTP or DBT') and its bromine substituted derivatives 4‐bromodibenzothiophene (4‐BDBTP) as additives in all‐PSCs [171]. The bromine substitution strategy can form enhanced intermolecular interactions with the active layer materials, which is beneficial for controlling molecular aggregation and crystallization, leading to more ordered molecular packing and well‐defined fibril networks in blend films. Consequently, the PM6:PY‐DT OSCs processed with 4‐BDBTP exhibit an impressive PCE of 19.30% (certified 18.82%), outperforming the as‐cast device (15.73%) and DBTP‐treated device (18.36%). In 2025, Kan's team reported a dual additive strategy by combining solid additive dibenzofuran (DB' or DBF) and solvent additive 1‐CN to synergistically control morphology of the active layer [172]. The results reveal that additive DB' plays a crucial role in enhancing the molecular crystallinity, whereas additive 1‐CN serves to regulate molecular orientation. Finally, the PM6/L8‐BO(DB' and 1‐CN) OSCs exhibit an excellent PCE of 19.58%, outperforming the devices treated with DB' (18.32%) or 1‐CN (18.74%) alone and the as‐cast device (17.67%). Moreover, they utilized DBF and 2‐bromodibenzofuran (2Br‐DBF) as solid additives to regulate the aggregation behavior of Y6 derivatives [173]. Ultimately, the 2Br‐DBF‐treated PM6/L8‐BO OSCs afford an impressive PCE of 20.02%. Recently, Li et al. further applied the solid additives DBF and 2‐BDBF (namely 2Br‐DBF) to all‐PSCs PM6/PY‐IT, demonstrating their powerful capability for morphological control [203].

In 2021, Bao et al. reported a dual‐additive strategy by employing volatile solid additive dithieno[3,2‐*b*:2',3'‐*d*]thiophene (DTT) and solvent additive 1‐CN to optimize morphology of the photoactive layer [174]. The findings suggest that the highly crystalline DTT can suppress excessive self‐assembly of the acceptors during film casting, enabling refined molecular packing and phase separation upon volatilization of DTT. The blend films processed with dual‐additive exhibit more ordered face‐on orientation and better phase separation compared to the 1‐CN‐treated blend films. Particularly, the as‐prepared PTQ10:*m*‐BTP‐PhC6:PC_71_BM ternary OSCs afford an excellent PCE of 18.89%. Recently, Zhang et al. have once again demonstrated that additive DTT can optimize morphology of active layer in the solid state, yielding high PCEs of 18.50% and 20.01% for the binary PM6:BTP‐eC9 and ternary PM6:BTP‐eC9:L8‐BO OSCs, respectively [175]. More importantly, additive DTT can serve as a kinetic stabilizer in the solution state, which can prolong the shelf‐life of the precursor ink from 5 to 10 days while preserving 90% of its initial device efficiency. In 2023, Sun and co‐workers utilized dithienothiophene isomers (i.e., dithieno[2,3‐*b*:3',2'‐*d*]thiophene (DTT‐1), dithieno[2,3‐*b*:2',3'‐*d*]thiophene (DTT‐2) and DTT‐3 (DTT)) as volatile solid additives in PM6:L15‐based all‐PSCs fabricated with *o*‐XY [176]. The results reveal that additive DTT‐2 with asymmetric structure and the weakest molecular interaction with the acceptor enables a more compact molecular stacking and superior crystallinity in the blend films. Consequently, the DTT‐2‐treated PM6:L15 devices offer a champion PCE of 18.72%. Subsequently, they further employed SL‐0 (DTT‐2) and its analogues (i.e., SL‐1 and SL‐2) as solid additives in all‐PSCs [177]. Finally, the SL‐1‐treated PM6:PY‐IT devices deliver a higher PCE of 19.19% compared to the SL‐0 (18.37%) and SL‐2‐treated (16.60%) OSCs.

In addition, thieno[3,2‐*b*]thiophene (TT) and its halogenated derivatives have been reported as additives. In 2023, Cao et al. reported a novel solid additive 2,5‐dichlorothieno[3,2‐*b*]thiophene (TT‐Cl) in various non‐fullerene OSCs [178]. Additive TT‐Cl exhibits a high crystallinity and strong interactions with the host materials, resulting in optimized molecular crystalline and vertical phase distributions in the blend films. Consequently, the TT‐Cl‐processed PM6:Y6 and PM6:D18:Y6 devices afford excellent PCEs of 18.20% and 18.95%, respectively. Interestingly, Zhang et al. also designed a series of additives (namely, TT‐H (TT), TT‐Cl, TT‐Br and DTT‐Cl) to regulate the crystallization behavior of blend films [179]. After different solid additives treatment, the corresponding PM6:Y6 devices show an improved PCE. Notably, the TT‐Cl‐treated PM6:Y6 devices exhibit a champion PCE of 17.5%. Additionally, an impressive PCE of 19.3% is obtained in TT‐Cl‐processed D18:BTP‐eC9‐4F OSCs. Later, Chen et al. employed two solid additives of TT and 3,6‐dibromothieno[3,2‐*b*]thiophene (TTB) to modulate the blend film morphology [180]. As a result, the TT‐ and TTB‐treated PM6:Y6 devices afford high PCEs of 17.75% and 17.33%, respectively, which are higher than those of the as‐cast device (15.94%) and 1‐CN‐treated OSC (17.17%). In 2024, Zhou et al. applied TT, TTBB (TTB) and 3‐bromo‐6‐iodio‐thieno[3,2‐*b*]thiophene (TTBI) as solid additives in non‐fullerene OSCs [181]. Ultimately, compared with the as‐cast devices, the TT‐, TTBB‐ and TTBI‐processed PM6:L8‐BO OSCs show enhanced PCEs of 18.2%, 19.1% and 18.7%, respectively. Moreover, the D18(TTBB)/L8‐BO(TTBI) device prepared with LbL method shows an excellent PCE of 19.9 %. In 2025, Song et al. used 3,6TTBr (TTB/TTBB) as additive to elucidate its working mechanism [182]. Finally, a high PCE of 20.1% is achieved in the PM6:D18:L8‐BO OSCs treated with additive 3,6TTBr. In 2024, both Liu et al. and Wang et al. reported a solvent‐solid hybrid additive strategy by combining additives TT and 1‐CN to optimize molecular packing and phase separation [204, 205], which underscores the critical role of the solvent‐solid hybrid additive strategy in achieving precise modulation of active layer morphology.

Moreover, six‐membered nitrogen‐containing heterocycles (e.g., pyridine‐, pyrazine‐, triazine‐ and pyrimidine‐based derivatives) have been reported as effective volatile additives in recent studies. In 2021, Yan et al. employed volatile solid additive Bipy to modulate the vertical phase separation of the J51:N2200 blend, resulting in a vertical compositional gradient with donor J51 enrichment at the bottom and acceptor N2200 accumulation at the top [183]. Ultimately, compared with the as‐cast device (7.37%), the Bipy‐treated J51:N2200 device delivers a higher PCE of 8.73% due to its increased open‐circuit voltage (*V*_oc_) and FF. In 2024, Liu et al. developed a novel solid additive methyl nicotinate (MN) in PM6/BTP‐eC9 OSCs fabricated with LbL blade‐coating method, which was exclusively added to the non‐halogenated solution of donor PM6 [184]. The findings suggest that additive MN can effectively modulate the molecular aggregation and crystalline orientation of donor PM6, leading to enhanced and balanced charge transport within the blend films. After introducing additive MN, the efficiency has been increased from 16.93% to 18.42% for the small‐area devices. More importantly, the as‐prepared large‐area module with an area of 28.82 cm^2^ delivers an excellent efficiency of 16.04% (Figure 13d). Subsequently, Hu et al. employed 2‐chloro‐5‐iodopyridine (PDCI) as a solid additive to control preaggregation behavior of Y‐series non‐fullerene acceptor in solution [185]. The results demonstrate that additive PDCI primarily interacts with the acceptor, thereby enabling enhanced and more ordered phase‐aggregation in solution. Ultimately, the PCE of PM6:Y6 OSCs has been dramatically improved from 16.12% to 18.12% after treatment with additive PDCI. Additionally, a champion PCE of 19.02% is obtained in PM6:BTP‐eC9 OSCs. Subsequently, they further used 3,5‐dichloropyridine (PDCC) as an additive to optimize the self‐assembly behavior of the non‐fullerene acceptors [186]. As a result, the PM6:L8‐BO OSCs prepared with PDCC show a high PCE of 20.47%. Additionally, the PDCC additive strategy enables high‐performance OSCs module (19.3 cm^2^) with a PCE of 15.79%. In 2025, Chen et al. applied 3,5‐dibromopyridine (DBP') and its derivatives (i.e., monomethoxy substituted 2‐methoxy‐3,5‐dibromopyridine (M‐DBP) and dimethoxy substituted 2,6‐dimethoxy‐3,5‐dibromopyridine (DM‐DBP)) as solid additives to regulate the molecular aggregation and stacking in D18:L8‐BO system [187]. Notably, methoxylated functionalization of DBP' significantly alters their ESP, dipole moment and non‐covalent interactions with the host materials. As a result, a champion PCE of 19.18% is obtained in the M‐DBP‐treated D18:L8‐BO binary OSCs, significantly outperforming other counterparts. In 2026, Li et al. demonstrated that the additive 2,3,5‐trichloropyridine (TCPy) could effectively regulate the aggregation behavior of the donor D18 and simultaneously guide the nucleation and crystallization process of the acceptor L8‐BO [188]. Consequently, the TCPy‐treated D18:L8‐BO OSCs show a higher PCE of 20.13% compared to the control device (18.49%).

Meanwhile, in 2024, Tu et al. developed two cyano‐functionalized pyrazine derivatives (3,6‐dibromopyrazine‐2‐carbonitrile (CNPz) and 3,6‐dibromopyrazine‐2,5‐dicarbonitrile (DCNPz)) as solid additives to improve the photovoltaic performance of OSCs [189]. The results indicate that additive CNPz can predominantly interact with the backbone of donor PTQ10, resulting in a favorable morphology with compact molecular stacking and enhanced molecular crystallinity in the blend film. Consequently, the CNPz‐treated PTQ10:*m*‐BTP‐PhC6 OSCs offer a higher PCE of 19.67% compared to the as‐cast device (18.73%) and DCNPz‐processed device (18.34%). In 2025, Li et al. reported solid additives of 2,5‐dibromoprazine (DBP'') and 2‐bromine‐5‐iodopyrazine (BIP) and incorporated them into the active layers prepared via LbL method, which can enhance the molecular stacking and crystallinity of acceptor [190]. Consequently, the BIP‐treated PM1/BTP‐eC9 device shows a higher PCE of 19.63% compared to the control device (18.38%). Moreover, an impressive PCE of 20.11% is obtained in PM1/BTP‐eC9:eC9‐2Cl fabricated with the non‐halogenated solvent toluene. In 2025, Wang et al. proposed a halogen‐free solid additive 2‐(methylthio)pyrazine (T‐PZ) in non‐fullerene OSCs [191]. The sulfur atom in T‐PZ can interact with the cyano group in acceptor Y6, which not only enhances the crystallinity of acceptor but also simultaneously optimizes the molecular aggregation of both donor and acceptor. As a result, the efficiency of PM6:Y6 OSCs has been substantially increased from 16.85% to 19.47% after treatment with additive T‐PZ. In particular, a remarkable PCE of 20.14% is obtained in the PM6:BTP‐TO2:AT‐β2O device processed with T‐PZ. In 2024, Cheng et al. reported a triazine‐based volatile solid additive, namely 2,4,6‐trichloro‐1,3,5‐triazine (TCT), to regulate the morphology of the active layer [192]. The results indicate that TCT shows strong interactions with both donor and acceptor, thereby optimizing molecular packing and arrangement. Ultimately, the TCT‐processed D18:L8‐BO OSCs deliver an excellent PCE of 19.50%, which is higher than that of the control device (18.13%). Moreover, the TCT‐treated devices exhibit outstanding stability, which can maintain 90% of its initial PCE after 960 h of storage in the glovebox. Intriguingly, Li et al. introduced additive TCT into the PM6:BTP‐eC9 system fabricated with the non‐halogenated solvent *o*‐XY [193]. As a result, the TCT‐treated OSCs without thermal annealing afford a higher PCE of 18.18% compared to the as‐cast device (16.43%). In 2025, Wang et al. employed 2,4,6‐tribromopyrimidine (TBP) as solid additive to elevate relative dielectric constant values of organic semiconducting materials [194]. The TBP treatment can effectively optimize molecular stacking, induce vertical phase separation and extend the exciton diffusion length in thick films, leading to significantly improved photovoltaic performances of OSCs. As a result, the efficiency of D18:L8‐BO OSCs has been significantly enhanced from 18.25% to 20.87% (certified 20.37%), which can still maintain 19.23% when the thickness is increased to 300 nm. Recently, Wu et al. developed two isomeric solid additives, *syn*‐dithieno[2,3‐*b*:3',2'‐*e*]pyrazine (*syn*‐DTPy) and *anti*‐dithieno[2,3‐*b*:3',2'‐*e*]pyrazine (*anti*‐DTPy), to regulate molecular aggregation of the host materials [195]. Notablty, additive *anti*‐DTPy not only facilitates the formation of dense and well‐ordered molecular packing within the acceptor L8‐BO but also induces tighter π‐π stacking in the D18:L8‐BO blend film, thereby leading to optimized charge transport pathways. As a result, the D18:L8‐BO OSCs treated with *anti*‐DTPy deliver a higher PCE of 20.5%, which is higher than that of the *syn*‐DTPy‐processed device (19.6%) and the as‐cast device (18.9%).

Notably, thiazole derivatives can also serve as volatile solid additives to fine‐tune the blend film morphology and thus improve the device performance. In 2024, Zhang et al. utilized volatile solid additives of 2,5‐dibromothiazole (DBrTz) and 2‐bromo‐5‐iodothiazole (BrITz) to modulate the film‐forming kinetics of the PM6:L8‐BO blend films [196]. Compared to BrITz, DBrTz exhibits higher volatility and can be removed during the film‐forming process. Specifically, during film deposition, chloroform evaporates initially, confining DBrTz within the forming film (pseudo‐dry film state). Subsequently, DBrTz gradually volatilizes, persistently inducing the molecular aggregation and stacking of acceptor L8‐BO. The resulting film shows tighter π‐π stacking and more ordered long‐range aggregation of L8‐BO, increased face‐on orientations, and favorable vertical phase distribution, which facilitates charge generation and charge transport (Figure 13e). Ultimately, the DBrTz‐treated PM6:L8‐BO OSCs afford a higher PCE of 19.0% compared to the as‐cast device (17.4%) and BrITz‐treated device (18.2%). Later, Wei et al. developed a series of chlorinated thiazole solid additives (i.e., TZ‐Cl, TZ‐2Cl and TZ‐3Cl) with varying numbers of chlorine atoms [197]. The results suggest that the intermolecular interactions between additives and host materials gradually strengthen with increasing chlorine substitution, thereby allowing effective regulation of the crystallinity and aggregation states of both donor and acceptor. As a result, the PM6:L8‐BO OSCs processed with additives of TZ‐Cl, TZ‐2Cl and TZ‐3Cl deliver high PCEs of 18.3%, 18.5% and 19.8%, respectively, surpassing the as‐cast device (17.7%). Particularly, an excellent PCE of 20.2% is achieved in the PM6:BTP‐eC9‐4F:DM‐F ternary OSCs. Recently, Mou et al. developed a series of halogenated thiazole units (i.e., 2,4‐DCl (TZ‐2Cl), 2Br‐4Cl, and 2,4‐DBr) as additives to precisely control the morphology of blend films [206]. Compared to 2,4‐DCl and 2Br‐4Cl, additive 2,4‐DBr shows moderate intermolecular interactions with both donor and acceptor, which prevent over‐aggregation while promoting ordered molecular packing and suitable phase separation. Consequently, the 2,4‐DBr‐processed D18:L8‑BO:eC9‐4F ternary device affords a high efficiency of 21.10% (certified 20.93%), which is one of the highest efficiencies reported so far. This work reconfirms that rational halogenation strategies for solid additives play a vital role in optimizing film morphology.

Moreover, benzothiadiazole can also be used as volatile solid additive in PSCs and all‐PSCs systems. In 2022, Oh et al. introduced solid additives of benzothiadiazole (BT') and its fluorinated derivatives (FBT and 2FBT) into PM6/Y6 blend films fabricated with LbL method to study their impacts on film morphology and device performance [198]. The results confirm that incorporating additive FBT into the PM6 layer induces optimal morphology, enabling efficient charge transport while mitigating charge recombination. Consequently, the as‐prepared PM6(FBT)/Y6 OSCs show a high PCE of 16.63%, outperforming the control devices. Furthermore, the FBT‐treated large‐area OSCs with areas of 0.92 cm^2^ and 2.50 cm^2^ afford high PCEs of 16.10% and 10.93%, respectively. Following this, Chen et al. designed and synthesized two isomers (i.e., 4,5‐difluorobenzo‐*c*‐1,2,5‐thiadiazole (SF‐1 or 2FBT) and 5,6‐difluorobenzo‐*c*‐1,2,5‐thiadiazole (SF‐2)) as solid additives in the PM6:PY‐DT all‐PSCs [199]. The findings reveal that the introduction of additives SF‐1 and SF‐2 into the PM6:PY‐DT blend film facilitates the formation of a continuous interpenetrating network with optimized phase separation and vertical phase distribution. Compared with SF‐1, the asymmetrical SF‐2 exhibit a larger dipole moment, leading to stronger interactions with the host materials, which promotes optimal phase separation in the active layer. Eventually, the PM6:PY‐DT devices processed with SF‐2 offer a high PCE of 18.83%, which is significantly higher than those of the as‐cast devices (15.17%) and SF‐1‐treated devices (17.83%). In 2025, Sun et al. reported three benzothiadiazole‐based solid additives with different halogen substitutions (namely difluoro‐BT' (BT‐2F or 2FBT/SF‐1), dichloro‐BT' (BT‐2Cl) and dibromo‐BT' (BT‐2Br)) in PM6:L8‐BO OSCs [200]. Compared with BT‐2F and BT‐2Cl, BT‐2Br exhibits superior donor/acceptor interactions, which enables a well‐ordered morphology by controlling the crystallization kinetics of the active layer. Ultimately, the BT‐2Br‐treated PM6:L8‐BO OSCs afford a higher PCE of 19.11% than the control device (18.31%). In 2026, Maqsood et al. further employed three halogenated benzothiadiazole derivatives in the LbL‐processed devices [201]. As a result, the 2*Br*BT(BT‐2Br)‐treated D18/L8‐BO:eC9‐4ClO OSCs deliver an impressive efficiency of 20.12%.

As mentioned above, volatile additives based on HAHs derivative have shown remarkable efficacy in both morphology control and device performance improvement. In the above reported work, the effects of the volatile additives on the active layer morphology and its film formation kinetics, as well as on carrier physical processes and device performance, have been thoroughly investigated. It is noteworthy that these additives feature a diversity of structures and types, which not only enriches the family of additives but also facilitates the investigation of their structure‐property relationships.

#### 2.1.5 Non‐aromatic Compounds

Apart from the above‐mentioned aromatic compounds, the organic transition metal compound ferrocene has been proven to be an effective volatile solid additive [207]. Furthermore, some non‑aromatic compounds have been successfully applied as volatile additives in non‐fullerene OSCs. Their chemical structures are shown in Figure 14, and the corresponding photovoltaic parameters are summarized in Table 6, demonstrating their outstanding capability in device performance enhancement.

**FIGURE 14:**
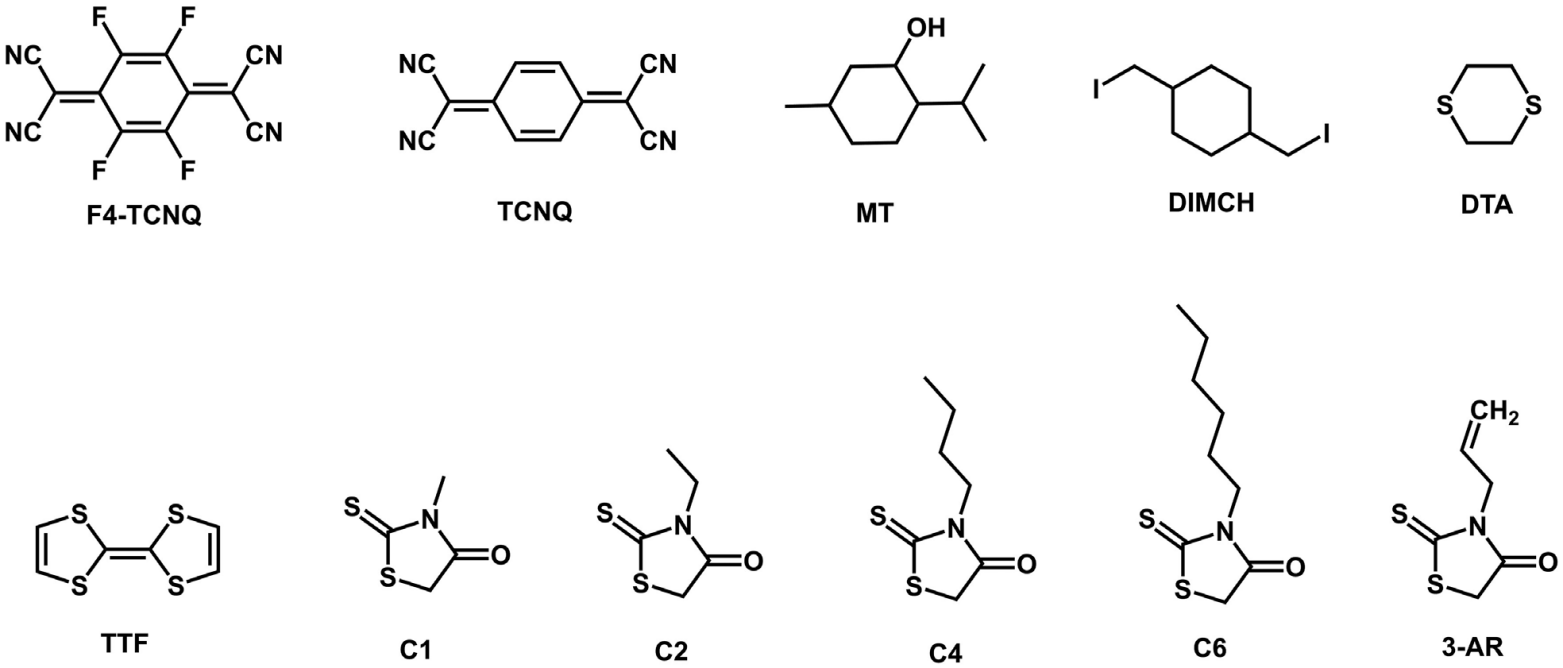
Chemical structures of volatile additives based on non‐aromatic compounds.

**TABLE 6:** Summary of photovoltaic parameters for devices processed with volatile additives based on non‐aromatic compounds.

Active layer	Additive	*V*_oc_ [V]	*J*_sc_ [mA cm^−2^]	FF [%]	PCE [%]	Refs.
FTAZ:IT‐M	w/o	0.965	17.80	68.69	11.89	[208]
	F4‐TCNQ	0.954	18.19	70.41	12.22	
J52:IEICO	w/o	0.866	13.18	55.2	6.30	[209]
	TCNQ	0.827	18.57	53.4	8.20	
PM6:L8‐BO	DIO	0.866	26.70	78.4	18.1	[210]
	DIMCH	0.906	26.35	78.9	18.8	
PM6:BO‐4Cl	w/o	0.853	27.55	76.95	18.09	[211]
	MT	0.853	28.35	77.76	18.81	
PBDB‐TF:eC9	DIO	0.835	27.2	79.9	18.1	[212]
	TTF	0.860	27.7	80.7	19.2	
PBDB‐TF:AITO‐2F:eC9	TTF	0.878	28.4	81.0	20.2	
PM6:L8‐BO	w/o	0.896	26.21	78.8	18.48	[213]
	DTA	0.900	26.89	81.1	19.64	
BTR‐Cl:N3	w/o	0.846	26.30	71.81	16.00	[214]
	C2	0.846	26.90	75.16	17.12	
MPhS‐C2:N3	w/o	0.863	27.03	74.85	17.46	[215]
	3‐AR	0.863	27.32	78.19	18.43	
	3‐AR	0.844	27.89	77.17	18.16^a^	

^a^ Certified result.

In 2018, Xiong et al. introduced 2,3,5,6‐tetrafluoro‐7,7,8,8‐tetracyanoquinodimethane (F4‐TCNQ) into the FTAZ:IT‐M blend films to study the effect of F4‐TCNQ on the blend film morphology and device performance [208]. The results indicate that F4‐TCNQ can effectively affect the mean‐square composition variation of the bulk rather than molecular packing and domain spacing, resulting in a PCE of over 12% for the FTAZ:IT‐M OSCs. However, the volatility of F4‐TCNQ was not extensively discussed in this work. In 2019, Yu et al. incorporated a volatile solid additive tetracyanoquinodimethane (TCNQ) into the J52:IEICO‐based OSCs [209]. A systematic investigation reveals that the introduction and volatilization of additive TCNQ can facilitate the intermolecular interactions of acceptors, yielding an optimized film morphology with better phase separation. Consequently, after treatment with TCNQ, the PCE of J52:IEICO OSCs has been substantially increased from 6.30% to 8.20%.

In 2024, Xiang et al. utilized a novel volatile solid additive, 1,4‐bis(iodomethyl)cyclohexane (DIMCH), to reduce energy loss in OSCs [210]. Compared with the additive DIO with a straight alkyl group, DIMCH with its unique cyclohexyl group exhibits a more concentrated ESP distribution, which can promote a more ordered molecular packing and restrain the excessive aggregation of acceptor L8‐BO. As described in Figure 15a, the DIMCH‐treated devices exhibit superior performance and lower energy loss than the DIO‐treated counterparts in various non‐fullerene OSCs systems (PM6:Y6/BTP‐eC9/L8‐BO). Particularly, the as‐prepared PM6:L8‐BO(DIMCH) devices deliver an impressive PCE of 18.8%. Meanwhile, Jia et al. employed an eco‐friendly and volatile additive, menthol (MT), in OSCs fabricated with non‐halogenated solvent [211]. The additive MT can effectively suppress excessive aggregation of acceptor and enhance crystallization during film formation. As a result, the MT‐treated PM6:BO‐4Cl device fabricated using the non‐halogenated solvent *o*‐XY affords a high PCE of 18.81%. Moreover, the large‐area module with an illumination area of 19.31 cm^2^ shows a remarkable PCE of 15.74%.

**FIGURE 15:**
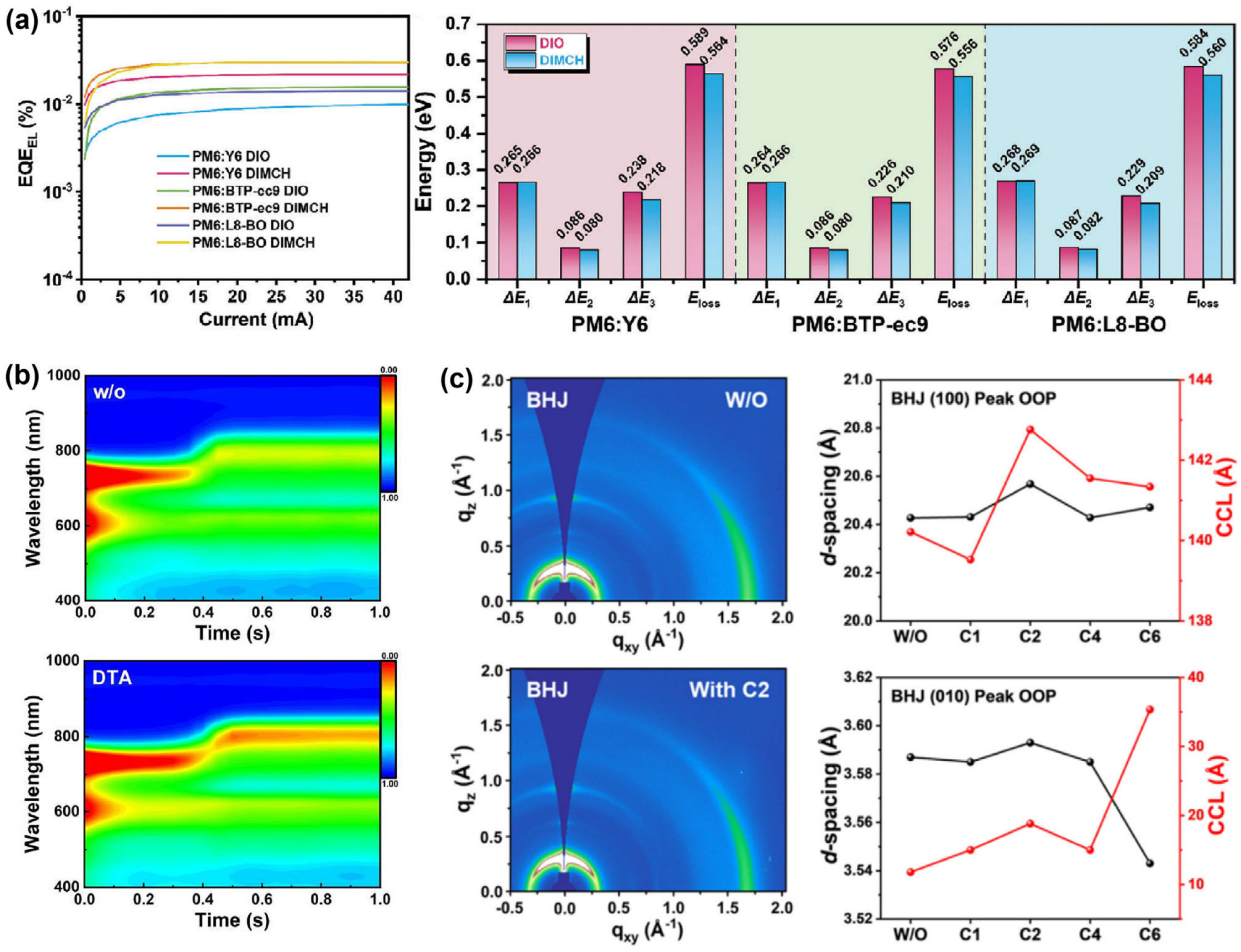
(a) EQE_EL_ curves and *E*_loss_ of the optimal devices. Reproduced with permission [210]. Copyright 2024, Wiley‐VCH. (b) In situ absorption spectra of the PM6:L8‐BO blend films with and without DTA treatment. Reproduced with permission [213]. Copyright 2026, Springer Nature. (c) 2D GIWAXS patterns of the BTR‐Cl:N3 blend films with and without C2 treatment, and the obtained *d*‐spacing and crystal coherence length (CCL) values of the (100) peak and (010) peak in the out‐of‐plane (OOP) direction. Reproduced with permission [214]. Copyright 2026, Elsevier.

In 2025, Chen et al. empolyed tetrathiafulvalene (TTF) as solid additive to optimize the aggregation of acceptor and reduce energy loss [212]. Analysis reveals that TTF exhibits strong interactions with the non‐fullerene acceptor, leading to optimized molecular stacking, enhanced exciton delocalization and reduced non‐radiative recombination. As a result, the TTF‐processed PBDB‐TF:eC9 OSCs show a higher PCE of 19.2% compared to the DIO‐treated device (18.1%). Additionally, the PBDB‐TF:AITO‐2F:eC9(TTF) OSCs afford an excellent PCE of 20.2%. In 2026, Guo et al. introduced a flavoring compound 1,4‐dithiane (DTA) as an additive to regulate the molecular crystallinity of the photoactive layer (Figure 15b) [213]. Studies have shown that the additive DTA first induces the donor to construct a polymer network framework, which then guides the subsequent crystallization process of the acceptor, leading to optimized blend morphology. As a result, the DTA‐processed PM6:L8‐BO OSCs afford a higher PCE of 19.64% compared to the control device (18.48%).

In 2025, Cao et al. developed rhodanine‐based compounds (i.e., 3‐methylrhodanine (C1), 3‐ethylrhodanine (C2), 3‐butylrhodanine (C4) and 3‐hexylrhodanine (C6)) to regulate the film formation kinetics in the BTR‐Cl:N3 system [214]. Among them, C2 can finely tune the donor‐acceptor miscibility and prolong the film‐formation time, leading to optimized morphology with appropriate phase separation, uniform vertical distribution, and enhanced molecular stacking (Figure 15c). Ultimately, the C2‐processed BTR‐Cl:N3 OSCs deliver a higher PCE of 17.12% compared to the as‐cast device (16.00%). Moreover, they further employed 3‐AR as solid additive in the MPhS‐C2:N3 system [215]. Notably, the resultant MPhS‐C2:N3 device exhibits a remarkable efficiency of 18.43% (certified 18.16%).

In conclusion, aromatic compounds are not the only choice for volatile additives. Non‐aromatic compounds can also serve as volatile additives to effectively fine‐tune film morphology of photoactive layer and enhance device performance. Although the continuous emergence of non‐aromatic compound additives has accelerated the diversification of volatile additives, their variety and quantity still lag far behind those of the aromatic compounds mentioned above, which warrants further attention.

### 2.2 Working Mechanism of the Volatile Additives

As aforementioned, a variety of volatile additives have been successfully developed for morphology control and performance enhancement in OSCs. As shown in Figure 16a, most volatile additives are predominantly derived from the fragments of polymer donor or non‐fullerene acceptor materials and their derivatives, which also provides guidance for the development of novle volatile additives.

**FIGURE 16:**
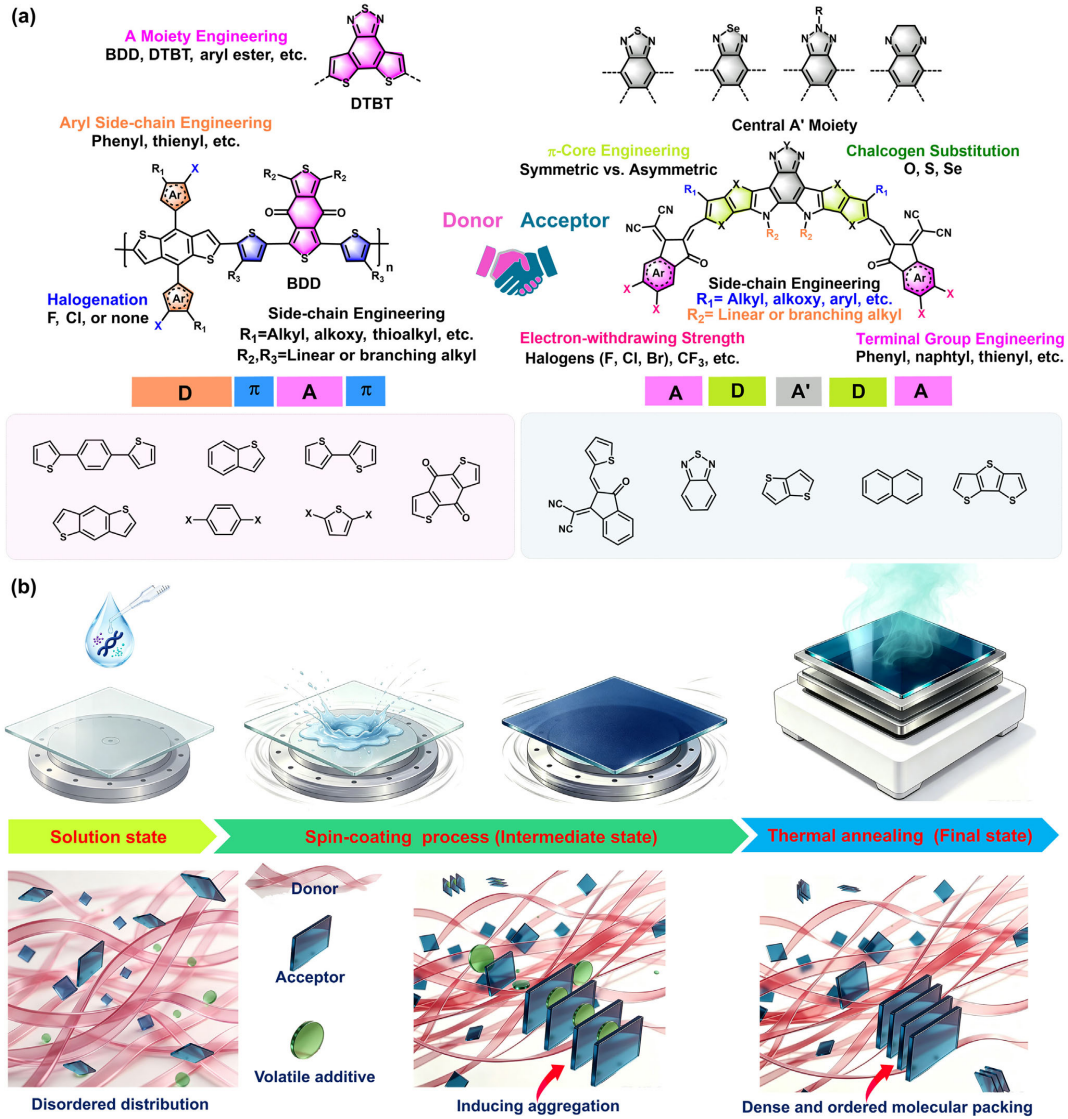
(a) Relationship between volatile additives and D‐A copolymer donor/A‐DA'D‐A type non‐fullerene acceptor materials. Reproduced with permission [216]. Copyright 2022, American Chemical Society. (b) Schematic illustration of volatile additive‐induced film formation and morphological evolution of the active layer.

Furthermore, the working mechanism of the volatile additives is discussed, which is divided into three stages based on the film formation process (Figure 16b). (1) The first stage is the solution state, in which the active layer molecules tend to adopt a disordered arrangement, and the introduction of volatile additives can change the miscibility of the active layer components. (2) The second stage is the film formation process via spin‐coating, where the active layer undergoes the liquid‐to‐solid transition driven by centrifugal force. The introduction of volatile additives can regulate the film formation kinetics of the active layer. During this process, the additives selectively interact with the donor, the acceptor, or both via non‐covalent interactions, such as halogen bonding, π‐π stacking, and dipole‐dipole interactions [31, 40, 52, 59], thereby inducing ordered molecular aggregation of the active layer. (3) The third stage is the thermal annealing stage. Under thermal treatment, the residual additives are completely removed (some additives may have completely evaporated during spin‐coating); and this provides the driving force for the self‐assembly and rearrangement of the donor and acceptor, thereby leading to dense and ordered molecular packing and suitable phase separation within the active layer [33, 162]. Finally, the optimized morphology can facilitate enhanced light absorption and spectral response, excellent charge generation, transport, extraction, and collection, as well as suppressed charge recombination, leading to improved photovoltaic performance [40, 196].

Consequently, the following conditions are essential for a high‐performance volatile additive: (1) strong intermolecular interactions with the active layer components (donor, acceptor, or both of them) via non‐covalent bonds; (2) good volatility (either self‐volatilization during film deposition or through post‐annealing at appropriate temperatures) [77, 178].

## 3 Effects of Volatile Additives on the Interfacial Layer

Notably, volatile additives can not only improve the molecular aggregation and packing of the photoactive layer, but also are applicable to the cathode or anode interfacial layers. As discussed in Section 2.1.2 above, volatile solid additives DCB, CBB and CIB were demonstrated to effectively optimize the blend morphology and device performance [48]. Given their unique in situ removable ability, these additives were further employed to refine the aggregation of the cathode interlayer PDINN. The DFT calculations indicate that the addition of CBB during PDINN interlayer fabrication primarily enhances the interactions between the PDINN and L8‐BO. Moreover, the CBB‐ and CIB‐processed PDINN interlayers can effectively reduce the non‐radiative recombination of devices, leading to higher *V*_oc_. As a result, the D18:L8‐BO OSCs fabricated with DCB‐, CBB‐ and CIB‐treated PDINN interlayers afford impressive PCEs of 18.98%, 19.38% and 19.26%, respectively, outperforming the control device (18.58%). Intriguingly, they also introduced volatile solid additives of DIB and DTT into the interfacial layer, yielding low PCEs of 3.75% and 0.19%, respectively. After thermal annealing treatment, the resulting DIB‐ and DTT‐treated devices exhibit poor PCEs of 10.96% and 8.59%, respectively, which remain lower than that of the untreated PDINN‐based device. These results demonstrate that the use of an in situ removable solid additive for cathode interlayer optimization holds a clear advantage over traditional volatile solid additive approaches. Interestingly, Zhang et al. used the solid additive DIB in the ZnO system to control nanoparticle stacking [217]. As shown in Figure 17a, solid additive DIB can firmly adhere to the surfaces of ZnO nanoparticles during the film‐forming process due to strong intermolecular interactions, which provides steric hindrance, suppressing aggregation and promoting uniform film coverage. After thermal annealing, the sublimation of the additive DIB promotes the oriented attachment of ZnO nanoparticles driven by dipolar interactions, while simultaneously enhancing interparticle Zn‐O bonding, thus effectively passivating oxygen vacancies. Consequently, the device based on DIB‐processed ZnO exhibits a high PCE of 20.1% (certified 19.8%), as well as exceptional thickness tolerance and operational stability.

**FIGURE 17:**
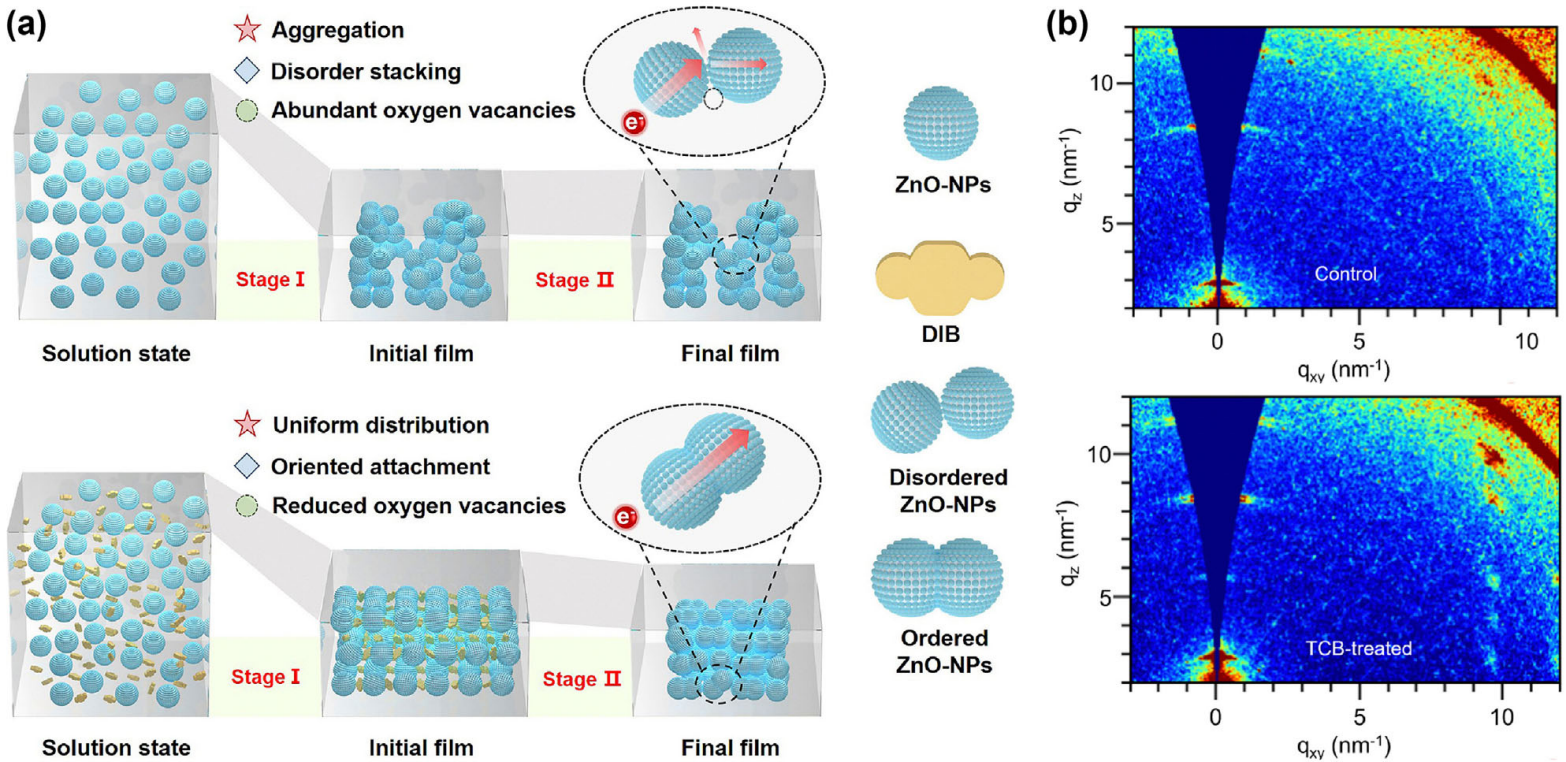
(a) Schematic diagram of the structural and electronic modulation of DIB‐processed ZnO nanoparticle. Reproduced with permission [217]. Copyright 2025, Royal Society of Chemistry. (b) 2D GIWAXS patterns of the 4PADCB‐based SAM layer with and without TCB treatment. Reproduced with permission [218]. Copyright 2025, Royal Society of Chemistry.

Conversely, Sun et al. introduced the volatile solid additive TCB into the anode interlayer constructed from self‐assembled molecule (SAM) 4PADCB [218]. The results reveal that the interaction between the conjugated backbone of 4PADCB and additive TCB induces a highly ordered molecular stacking with improved face‐on orientation in the SAM layer after TCB evaporation (Figure 17b). Meanwhile, this modification can also optimize the film formation kinetics of the photoactive layer, leading to enhanced molecular crystallinity and favorable vertical phase separation in blend film. Consequently, the PM6:BTP‐eC9 OSCs fabricated with TCB‐treated SAMs afford a higher PCE of 20.06% (certified 19.24%) compared to the control device (19.35%). Notably, the corresponding perovskite‐organic tandem solar cells deliver an excellent PCE of 26.09%.

## 4 Summary and Outlook

In summary, volatile additive is a simple and effective strategy for morphology control in OSCs. Moreover, significant advances have been made in the varieties, mechanisms of action and application methods of volatile additives. However, some issues remain to be further studied and are summarized as follows:

(1) Developing novel volatile additives

In recent years, a large number of studies on volatile additives have been reported. As aforementioned, most of them are based on benzene or thiophene derivatives, which suggests that the development of volatile additives is becoming increasingly homogeneous. Therefore, the development of novel volatile additives is urgently needed, especially low‐toxicity, halogen‐free volatile additives, recyclable additives, and volatile additives suitable for all‐small‐molecule systems. Moreover, the design of volatile additives must transition from empirical screening to rational molecular engineering. The integration of machine learning with high‑throughput experimentation to build structure‐property‐performance relationships accelerates the discovery of optimal additives without relying on laborious trial‐and‐error methods.

(2) Deepening the action mechanism of volatile additives

In previous work, the effects of additives on the film formation process of the active layer, molecular aggregation and packing behavior, as well as charge carrier dynamics, were investigated in detail. However, the structure‐activity relationship between additive structure and device performance remains to be studied. For example, the influence of molecular planarity of additives on device performance remains to be further explored. Planar molecules, with low steric hindrance, can readily embed within the molecular matrix of the active layer [23, 27, 157]. In contrast, the twisted architectures may provide sufficient space to facilitate molecular reorganization [67, 219, 220]. Additionally, the underlying mechanisms of voltage loss induced by certain additive treatments needs to be further investigated.

(3) Developing novel joint strategy techniques

In fact, the use of the joint strategies, such as volatile additive‐assisted LbL deposition method, volatile additive‐assisted SVA method, and dual‐additives (combining solid additives and solvent additives), can synergistically optimize the active layer morphology and enhance device performance, which can effectively overcome the limitations of single strategy. Thus, developing novel joint strategies is a highly promising research direction.

(4) Matching large‐scale fabrication techniques

It is widely recognized that volatile additives exhibit excellent morphology control capability in small‐area devices fabricated via spin‐coating method, and they have been extensively reported and systematically studied. Obviously, the film‐formation kinetics of the active layer processed with volatile additives differ greatly between spin‑coating and blade‑coating fabrication processes. Although some works have indicated that volatile additives also perform well in blade‑coating large‑area fabrication processes, the effect of additives on the film‐formation kinetics of the active layer fabricated by blade‐coating is still poorly understood. Moreover, the behavior of the volatile additives may be highly dependent on the preparation process. For example, in situ removable additives can be removed by centrifugal force during spin‑coating, but the case is markedly different when devices are fabricated by blade‐coating. Overall, the influence of volatile additives on the film formation process of the active layer in large‐area fabrication processes still needs to be further studied.

## Author Contributions

**Jianqiang Qin**: conceptualization, data curation, formal analysis, funding acquisition, investigation, validation, Writing – original draft, methodology. **Zhiqin Lai**: methodology, data curation, formal analysis, funding acquisition. **Jiang‐Ling Shi**: methodology, data curation, formal analysis, funding acquisition. **Zheng Xiao**: methodology, data curation, formal analysis, funding acquisition. **Yuwei Duan**: methodology, data curation, formal analysis, funding acquisition. **Zhiping Hu**: methodology, data curation, formal analysis, funding acquisition. **Juan Du**: methodology, data curation, formal analysis, funding acquisition, supervision. **Qiang Peng**: resources, project administration, supervision, writing – review and editing, validation, conceptualization.

## Conflicts of Interest

The authors declare no conflicts of interest.

## Data Availability

The data that support the findings of this study are available from the corresponding author upon reasonable request.
